# Study protocol for a quasi-experimental evaluation of the whole school approach FREI DAY

**DOI:** 10.1186/s13690-026-02088-7

**Published:** 2026-10-13

**Authors:** Dominik Röding, Ricarda Brender, Sibel Ünlü-Reske, Anna Brager, Alicia Strauß, Jacqueline Kittel, Maike Stolz, Christian Krauth, Christiane Meyer, Ulla Walter

**Affiliations:** 1 Hannover Medical School, Institute for Epidemiology, Social Medicine, and Health Systems Research, Hanover, Germany; 2 Institute for Didactics of Natural Sciences, Leibniz University Hannover, Hanover, Germany

**Keywords:** Effectiveness, Natural experiment, Education for sustainable development, Health promoting schools, Implementation, Students, Key informants

## Abstract

**Background:**

The Sustainable Development Goals combine health, social justice, sustainability, and environmental protection. Schools offer a suitable setting for linking these goals and incorporating them into the development of children and youth. FREI DAY is a whole-school approach in German-speaking countries and at German schools abroad. It is designed as a curriculum-anchored learning format in which students work on self-chosen projects related to the Sustainable Development Goals. The approach aims to improve lifestyles and living conditions in both health and environmental sustainability, and to build schools’ capacity to transform into healthy and sustainable organisations. FREI DAY is theory-based and aligned with the United Nations Sustainable Development Goals. This study examines the effectiveness of FREI DAY and the conditions necessary for its success, providing recommendations for its implementation and further development.

**Methods:**

The study is designed as a natural experiment with a sequential design. Over a period of three years, at least 60 secondary schools, including vocational schools, and 30 primary schools will be surveyed during three survey periods. The schools themselves decide whether and when to implement the eight-phase FREI DAY intervention. Schools will be classified as being in the intervention period once they moved from phase 3 (prepare FREI DAY pilot implementation) to phase 4 (implement FREI DAY pilot). Schools that have not yet reached phase 4 or discontinue will be classified as being in the control period. This means that schools can switch from the control condition to the intervention condition and vice versa during the study period. In each survey year, at least 20 students per school will be surveyed, i.e., around 1,200 students per wave. In addition, five school key informants per school will be surveyed annually, making 300 per wave (open cohort design). At intervention schools, at least three members of the school board per wave will also be surveyed. Changes in the results will be evaluated using hierarchical linear models.

**Discussion:**

This is the first study to evaluate the effectiveness of FREI DAY. We expect FREI DAY to improve students’ well-being, life skills, environmental awareness and environmental responsibility. In addition, it is expected to strengthen the transformative capacity of schools, create a new culture of learning and sustainability, and strengthen collective resilience.

**Trial registration:**

This study was registered in the German Clinical Trials Register: DRKS00036310 on August 07, 2025.

**Supplementary Information:**

The online version contains supplementary material available at https://doi.org/10.1186/s13690-026-02088-7.

**Table:** 

Text box 1. Contributions to the literature	
• A major gap in public health research is addressed by evaluating a whole-school approach that links health promotion with sustainability and social equity, areas that are typically studied separately. • It provides an evaluation of an intervention explicitly aligned with the Sustainable Development Goals, using a natural experiment design.	
• By examining both student outcomes and changes at the school system level, the study advances understanding of how schools can build long-term capacity for health, sustainability, and equity. • The findings will inform public health policy about conditions under which the intervention can be effectively implemented across diverse contexts.	

## Background

The Sustainable Development Goals (SDGs) have emerged as a global blueprint emphasizing the intertwined nature of health, social equity, and environmental stewardship [1]. Public health frameworks have long underscored the importance of social determinants of health—concepts that overlap substantially with many SDG targets, including those related to wellbeing, inclusion, and ecosystem protection [2].

Educational institutions offer a highly strategic setting to address these interlinked objectives [3, 4]. The Health Promoting Schools (HPS) model—championed by WHO and UNESCO—embodies a whole-school approach to fostering health and wellbeing across students, staff, families, and the wider community [5]. This approach advances a comprehensive vision that integrates supportive policies, physical and social environments, capacity building, health skills, and community partnerships to create systemic change [5].

In Germany, Education for Sustainable Development (ESD) is institutionalized through a national action plan aligned with the UNESCO “ESD for 2030” framework [1, 6]. Since the adoption of the 2017 National Action Plan on ESD, which includes 130 strategic goals and 349 recommended actions, the initiative has been anchored structurally within the country’s educational landscape [6, 7]. ESD’s whole-system approach extends beyond classroom instruction to include school governance, infrastructure, and community collaboration—closely mirroring the whole-school paradigm [8, 9].

The “FREI DAY” initiative represents an innovative German adaptation of these converging paradigms: operationalizing the SDGs through school-centred, whole school approaches that aim to foster sustainable lifestyles, democratic participation, and collective wellbeing [10]. It constitutes a promising intervention to advance health promotion, social justice, and ecological sustainability in tandem [10].

Previous research on FREI DAY has mainly consisted of qualitative master’s and doctoral theses. These studies have provided exploratory insights into teachers’ and students’ experiences with FREI DAY, perceived opportunities and challenges of implementation, and the potential of FREI DAY as a format for education for sustainable development. However, there is still no prospective quantitative study evaluating whether FREI DAY is associated with measurable changes in student outcomes, such as well-being, life skills, environmental awareness, or environmental responsibility. Moreover, little is known about whether and under which conditions FREI DAY strengthens schools’ transformative capacity as an organisation. This constitutes the central research gap addressed by the present study. Our study, funded by the German Federal Ministry of Research, Technology and Space, aims to investigate the factors influencing the implementation and effectiveness of FREI DAY.

### Whole school approach FREI DAY

FREI DAY is a complex, multi-component intervention based on concepts of transformative education [11], self-determination theory [12], project-based learning [13], and ESD [14]. A systematic description of the complexity of FREI DAY based on ten dimensions of complexity [15] can be found in the supplement 1.

**Fig. 1:**
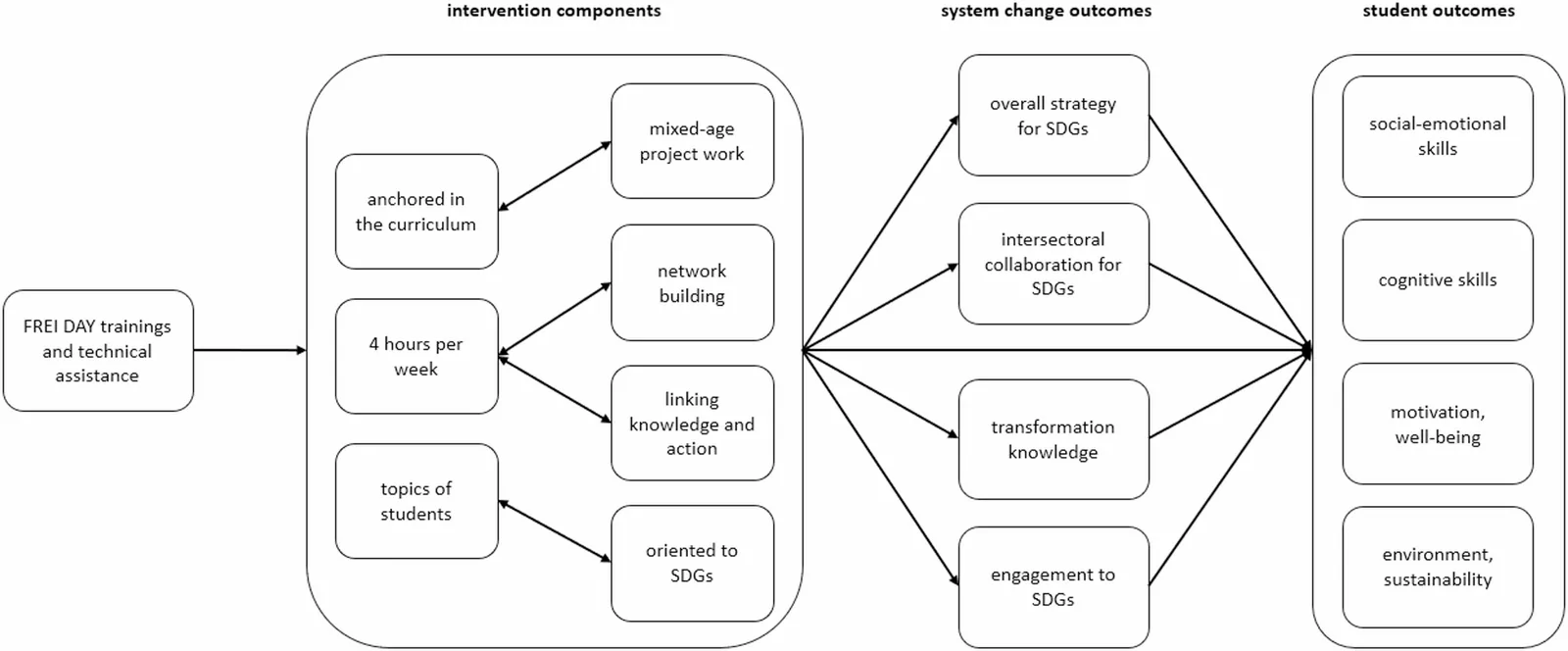
Logic model of FREI DAY and hypothesised pathways of change The figure illustrates the hypothesised mechanisms through which FREI DAY influences school-level and student-level outcomes. Training and technical assistance support the implementation of the seven core components of FREI DAY, which are expected to strengthen system change outcomes and subsequently improve student outcomes. Arrows indicate hypothesised causal pathways. SDGs = Sustainable Development Goals

Figure 1 illustrates the seven components of FREI DAY and the main causal pathways through which FREI DAY exerts its effects on schools as organisations (staff, structures, and processes) and on students. The causal pathway of this model starts with the provider of the intervention *Schule im Aufbruch gGmbH (SiA)*, who delivers trainings and technical assistance for schools to support them implementing and maintaining the intervention. This training and technical assistance includes online workshops and online as well as face-to-face network meetings for sharing ideas and experiences [16]. The implementation of FREI DAY consists the following eight phases in a time span of 2 to 4 years:

- Phase 1 – Inform: Schools obtain information from SiA about the objectives of FREI DAY, the support options available, and the implementation process, and participate in information events.
- Phase 2 – Finding allies: A FREI DAY team is formed to introduce and implement FREI DAY. The school community is informed of this and asked to provide support.
- Phase 3 – Prepare the pilot phase: The time frame and suitable classes are selected, teachers are offered trainings, and the introduction is planned based on the FREI DAY modules. The school establishes contacts with external cooperation partners.
- Phase 4 – Implement pilot phase: The pilot project is implemented and regularly documented for evaluation and further development if necessary. Schools recognise the conditions for success and the success factors of FREI DAY.
- Phase 5 – Evaluate the pilot phase: To evaluate the pilot project, all experiences relating to FREI DAY will be collected and questionnaires will be created for all participants in order to formulate the next steps for the further development of FREI DAY.
- Phase 6 – Anchoring the learning format: Schools adapt the FREI DAY concept templates to their circumstances, offering exchange formats for students, parents and colleagues. The school conference must decide to anchor FREI DAY.
- Phase 7 – Preparing for implementation: Continuing education and training are offered to the teaching staff, and the role of learning support in project-oriented work is introduced. The schools examine which additional resources they want to purchase and which rooms and learning spaces inside and outside the school can be used by the students.
- Phase 8 – Implementation: The schools have started implementing FREI DAY together with the school community and external cooperation partners. The students have been introduced to the principles of project work and are being supported in their learning process.

The seven components of the FREI DAY are (1) a curriculum anchored learning format (2) of at least four hours per week, (3) where students work in small groups (4) to come up with a project that addresses at least one of the SDGs (5) and then carry it out themselves (6) over the course of at least one school year and (7) supported by one or more internal or external collaboration partners. The role and task of the teachers is to support the student project groups in planning and execution of their projects. This often involves teachers helping project groups to obtain funding, purchase materials, obtain permits and establish collaborative relationships with internal and external project partners. It is not uncommon for these projects to only be realised if parents, various school staff and external local organisations, such as businesses or agents, participate in the project and carry out tasks with the students outside of school grounds and school hours. For example, when redesigning a city park, students need the support of the mayor and the local authority so that they can develop a landscape plan together. In other projects, for example, students got involved with refugee children, addressed everyday racism, organised clothes swaps and made everyday items through upcycling, or acted as energy detectives, formed climate councils or decided at children’s conferences that their school should become waste-free and plastic-free. Other students took a critical look at the so-called parent taxi and succeeded in getting almost all primary school children to travel to school by other means. In their local community, students used self-generated energy to light up local cycle paths or convinced the city administration to switch schools to green electricity [10]. Further project examples can be found in this video.

With regard to the effects of FREI DAY at the organisational level, we expect increases in (1) integrated strategy for SDGs, (2) intersectoral collaboration for SDGs, (3) transformational knowledge, and (4) engagement for SDGs. It is expected that the effects of FREI DAY on students will be mediated to a substantial extent by these four system-change outcomes. The intended student outcomes include social and emotional skills, cognitive skills, motivation and well-being, as well as a deeper awareness of the environment and sustainability [17].

## Methods and design

### Research aims and hypotheses

The primary aim of this study is to evaluate the effectiveness of FREI DAY as a whole-school approach for promoting health, well-being, and sustainability-related outcomes among students. We hypothesise that students in schools or classes exposed to FREI DAY will show greater improvements in well-being, optimism, self-efficacy, environmental awareness, and environmental responsibility than students not exposed to FREI DAY. A second aim is to examine whether FREI DAY strengthens schools’ transformative capacity. We hypothesise that schools implementing FREI DAY will show greater improvements in an integrated strategy for the SDGs, intersectoral collaboration for the SDGs, transformation knowledge, and engagement for the SDGs than schools in the control period. A third aim is to examine implementation conditions and potential moderators of effectiveness. We will therefore analyse whether intervention effects vary according to school-level characteristics, implementation quality, school team characteristics, and individual student characteristics.

### Study design and assignment method

**Fig. 2:**
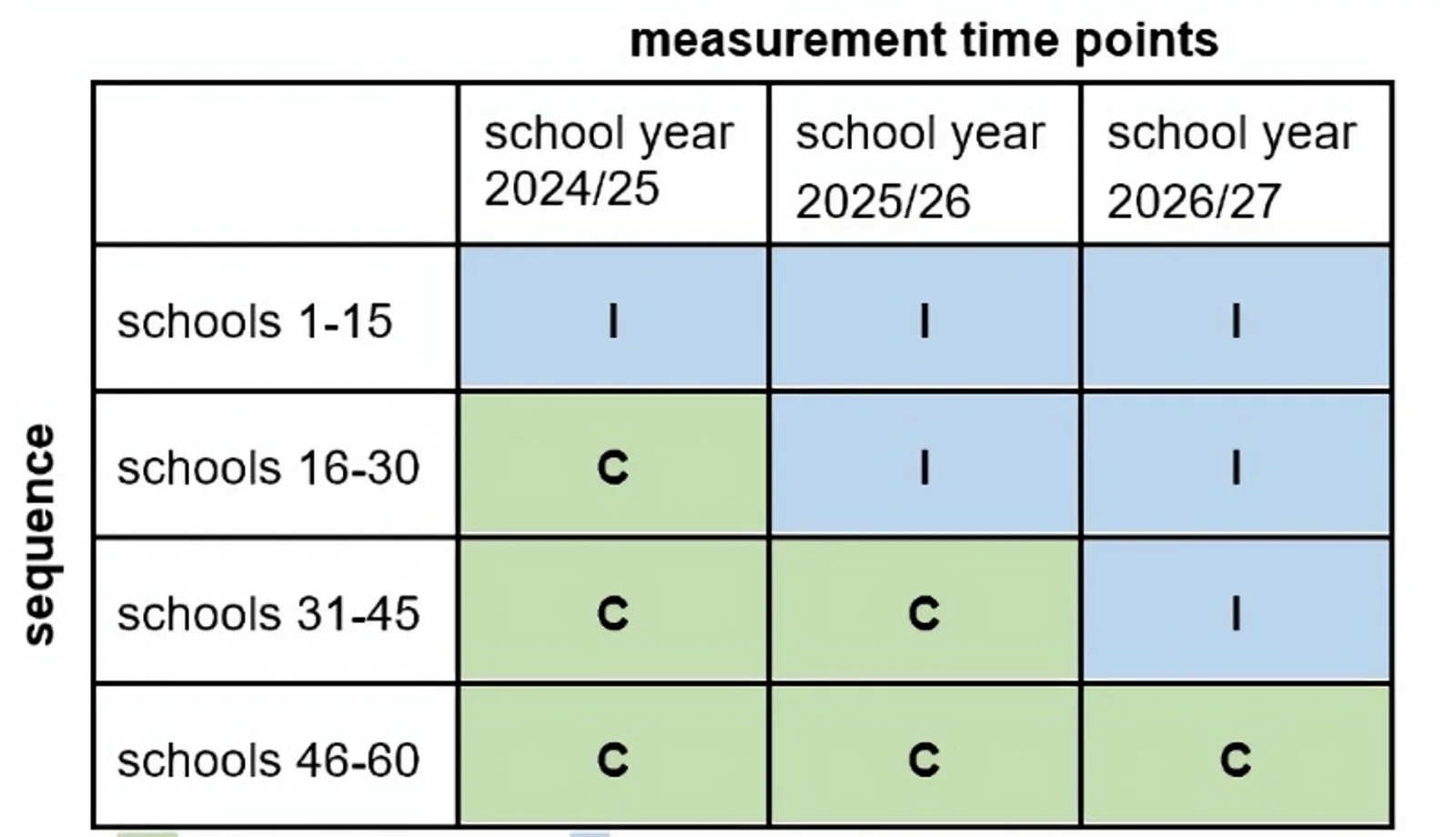
Non-randomised stepped-wedge design of the FREI DAY evaluation study

For our study, we are using the current spread of FREI DAY in German-speaking countries as a natural experiment [18, 19] with a sequential design, allowing us to refer to it as a non-randomised stepped wedge design (Fig. 2). To this end, at the start of the study, we recruit (1) schools that already implement FREI DAY, (2) schools that plan to pilot FREI DAY in the next two years and then, if appropriate, make it permanent, and (3) schools that do not plan to pilot FREI DAY in the next two years. This means that the schools themselves decide whether and, if so, when to implement FREI DAY. For components of FREI DAY that aim at systemic change (transformational capacity), in particular network building, schools are always the allocation units. In contrast, for components of FREI DAY whose immediate target group is students, such as the four hours of free time per week, skills, etc., classes or grade levels are usually the allocation units. This differentiation is particularly relevant for effectiveness analyses at the student level, as data is available within individual schools on both students who participate in FREI DAY and students who do not participate in FREI DAY.

Schools were grouped into four implementation sequences according to the timing of FREI DAY implementation. Schools could transition from the control period (C) to the intervention period (I) across the three annual measurement waves. C = control period; I = intervention period.

Based on the eight implementation phases described above, we will determine which of the two periods (control vs. intervention period) a school is in at a given data collection time point. At each survey wave, we receive information from the participating schools as to whether they are currently implementing FREI DAY or have implemented it. We classify schools that are at least in phase 4 of FREI DAY implementation as belonging to the intervention period. Schools that have not implemented FREI DAY or have not yet reached phase 4 are classified as belonging to the control period. If a school stop using FREI DAY during the study, we also classify it as belonging to the control period. Schools that we classify as part of the control period are a comparison group that does not implement FREI DAY. This classification is based on the annual Milestones and Benchmarks Tool and information provided by the participating schools at each survey wave. Since schools in Germany are encouraged to implement ESD, many schools carry out corresponding projects.

### Recruitment and eligibility of schools

**Fig. 3:**
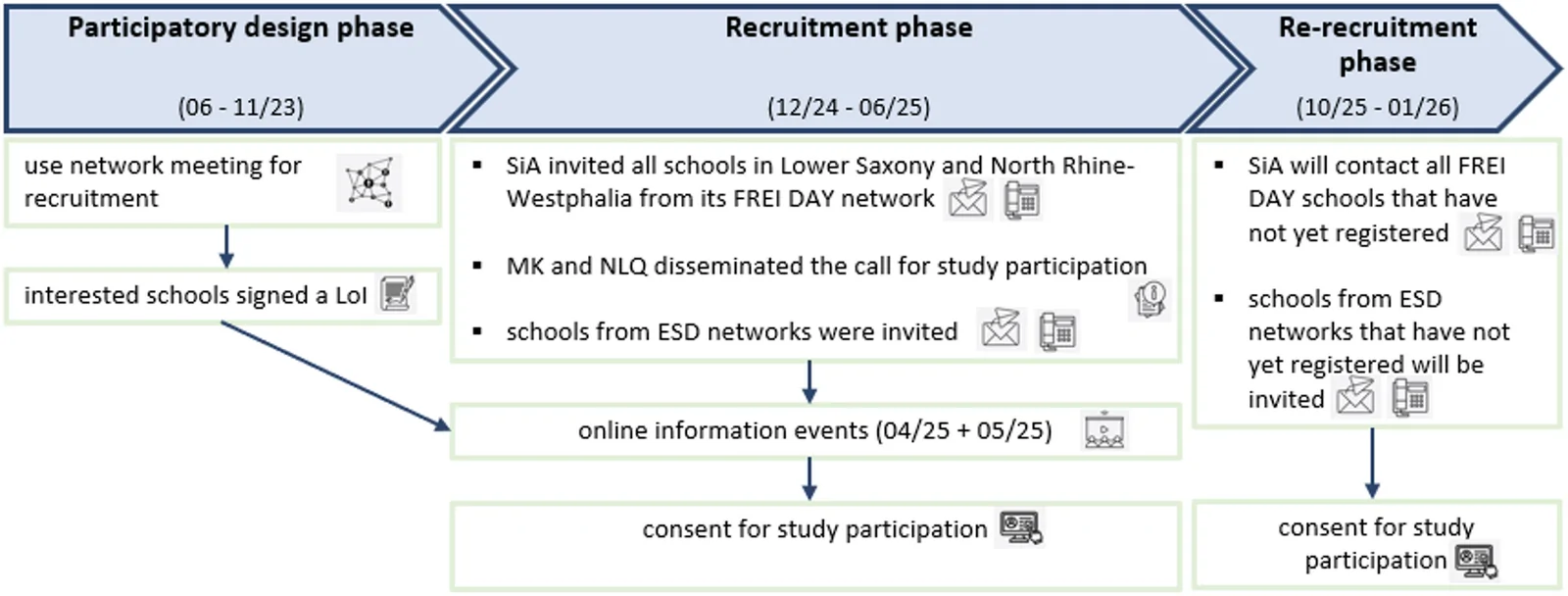
School recruitment process in Lower Saxony and North Rhine-Westphalia

The main phase of our study was preceded by a six-month participatory preliminary phase, during which we developed the study design and survey instruments in collaboration with the intervention provider SiA and schools that have already introduced FREI DAY or intend to do so. During the previous participatory preliminary phase in 2023, initial schools were invited to participate in the study at various network meetings and subsequently signed a letter of intent [20]. The majority of these schools are participating in the current study (Fig. 3).

The figure shows the procedures followed during participatory design phase, recruitment phase and re-recruitment phase, which were used to enrol schools into the FREI DAY evaluation study. Schools were recruited through the FREI DAY network, governmental education institutions and ESD networks. ESD = Education for Sustainable Development; LoI = Letter of Intent; MK = Lower Saxony Ministry of Education and Cultural Affairs; NLQ = Lower Saxony State Institute for School Quality Development; SiA = Schule im Aufbruch gGmbH.

The recruitment of schools at the beginning of the main phase of the study took place in various ways: (1) As the provider of the FREI DAY learning format, SiA invited all schools in Lower Saxony and North Rhine-Westphalia from its FREI DAY network to register for the study starting in February 2025. This network includes all schools that are informed about FREI DAY, are introducing it on a pilot basis or are actively implementing it. (2) The Lower Saxony Ministry of Education and Cultural Affairs and the Lower Saxony State Institute for School Quality Development were informed in December 2024 and disseminated the call for participation in the study. (3) Schools from other ESD networks were contacted by email as comparison schools. If no response was received, these schools were also contacted by telephone. These school networks aim to create learning environments in which ecological, social and equitable dimensions of sustainability are integrated and made tangible.

Interested schools were invited to attend an information event at the end of April or May 2025. Afterwards, the participating schools were sent the presentation slides from the information event, including FAQs and an overview of the content of the three quantitative surveys.

Schools could register to participate in the study, which consisted of three survey waves, by clicking on a registration link to SoSci-Survey. The contact persons named during registration then received an email for the first survey wave (May-September 2025) with information about the study procedure and the three different surveys (student survey, school key informant interview, school board interview), preview copies of the surveys, study information and data protection declarations, flyers and posters for the school key informant interview (SKI) to be displayed and distributed at the school, and a checklist and worksheets for students who are not allowed to participate, e.g. due to a lack of parental consent. The materials for the student survey were also sent by post, as they included photocopiable templates. On site, the contact persons coordinated the student survey and distributed access to the FREI DAY team members and school key informants in the school community.

For the second survey wave (February-June 2026), SiA will contact all FREI DAY schools that have not yet registered for and participated in the first survey wave in October/November 2025. Schools from the ESD networks that have not yet registered were also contacted again by email and invited to participate in the study.

To encourage participation in the study, all schools that take part receive a report on the data collected at their school after each survey. Schools can also participate in a webinar, during which they can ask questions about the constructs surveyed and their results. Schools will also be given tips on how to use the data for school development. Finally, participants are asked to inform the project team of any improvements that could be made to the data collection process at their school in future. The first survey wave webinars for the intervention schools (i.e., all schools in Phases 4–8) are scheduled for November/December 2025. Webinars for the comparison schools will take place at the beginning of December 2025. Webinars on the school report will also be offered in autumn after the two future survey waves.

### Participants, eligibility and data collection

**Fig. 4:**
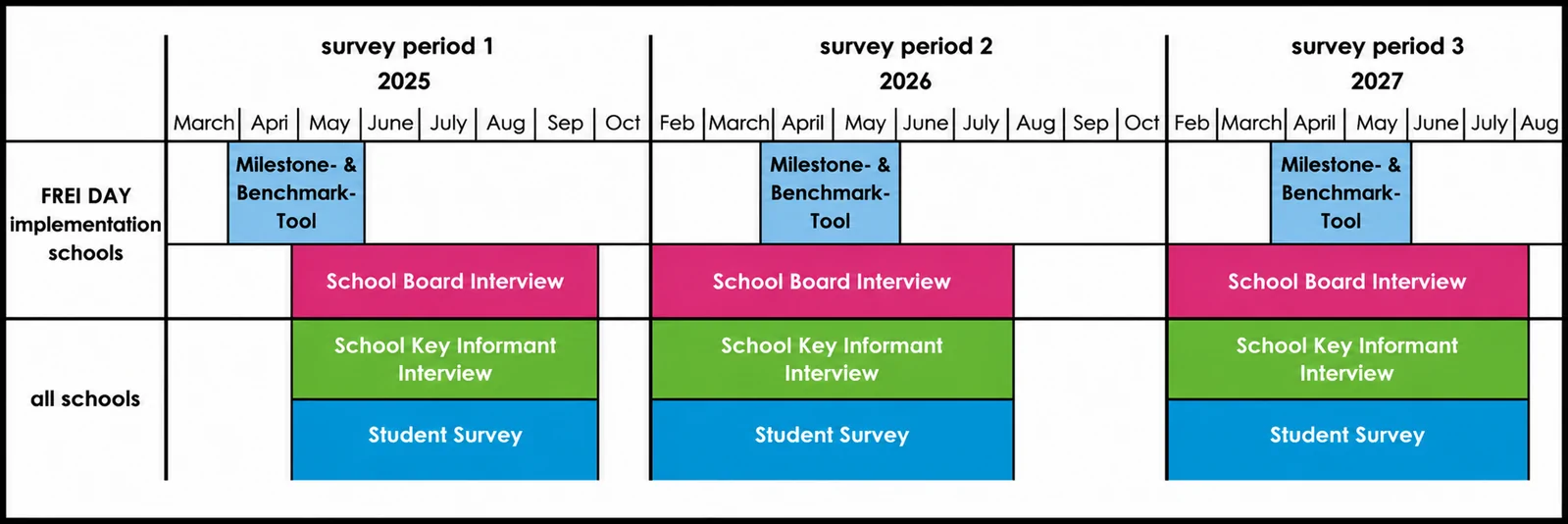
Survey schedule across three survey periods

Figure 4 provides an overview of the surveys that will be conducted in the participating schools. The survey instruments were specified and finalized during the participatory preliminary phase in a multi-month iterative process with teachers, partners, and SiA [20]. In addition to these surveys, qualitative empirical social research methods will be used in selected intervention schools.

The figure shows the timing of the four surveys conducted during the study. In survey period 1, three surveys began later than planned because approval from the school district was delayed. Schools in the intervention period are referred to as *FREI DAY implementation schools*, whereas *all schools* include both schools in the intervention and control period. The *Milestone & Benchmark-Tool* is completed by FREI DAY coordinators at the intervention schools and assesses the current status of implementation. The *School Board Interview* surveys the school board on factors that promote or hinder implementation. The *School Key Informant Interview* is directed at all members of the school community and is used to identify context-related and school-related factors. The *student survey* was used to assess students’ competencies, well-being, and environmental awareness.

In order to be allowed to conduct the planned surveys at schools in the federal state of Lower Saxony, an application for approval was submitted to the Regional Office for Schools and Education in Hanover (RLSB) in October 2024. The research project was approved in January 2025. No approval from the state school authority is required for surveys in schools in the federal state of North Rhine-Westphalia. Here, the school management decides on the implementation of empirical studies or surveys after consulting the school conference [21].

#### Student survey

At the beginning of each survey wave, participating schools receive a sample copy of the questionnaire (supplement 2 + 3; available in German), worksheets for students who are not allowed to participate e.g., due to a lack of parental consent (supplement 4 + 5; available in German), a checklist for conducting the student survey, data protection and consent forms, study information for parents and students, and an overview of the planned surveys. The schools organize the student survey themselves. Schools should conduct the survey during a lesson or a free period. The online survey takes about 35 (+/- 10) minutes to complete. Primary schools should only allow third and fourth graders to participate in the survey. The questionnaire has been adapted for these students. All schools are instructed to only allow students under the age of 14 to participate in the survey if they have submitted a written informed consent form from their parents to the school. In addition, a written informed consent form must also be provided by the students.

The schools were asked either to have a representative sample of their classes participate in the survey or to conduct a full survey, if possible. They were also informed that at least 20 students per school needed to participate in all three planned survey waves. The schools conducting FREI DAY were also informed that, if possible, they should encourage all students who are participating in FREI DAY, have participated in FREI DAY, or will participate in FREI DAY during the study period to take part in the survey. Furthermore, all schools were informed that, if possible, all students who participated in the first survey should also participate in the two follow-up surveys.

In order to be able to merge the longitudinal data sets of individual students at a later stage, students generate a personal pseudonym for themselves in each survey wave using the so-called Duisburg Code [22]. No personal data such as real names or personal addresses are collected. The student survey consists of validated multi-item scales and known indicators on the following topics: (1) sociodemographic data (e.g., gender, grade level, living situation and family constellation, migration background, life situation), (2) social and emotional skills, (3) cognitive skills, (4) motivation and well-being, (5) environmental awareness and responsibility, and (6) fear of the future.

#### School key informant interview (SKI)

School key informants are defined as all the essential persons from the school community, e.g., school administrators, teachers, (non-)educational staff, parents, or employees of the school authority. For the SKI, the contact persons at the participating schools receive a copy of the questionnaire (supplement 6; available in German), a survey link, a privacy policy, study information, an overview of the planned surveys, and flyers and posters to distribute and display at their schools for each survey wave. The contact persons distribute the survey link within the school community. In addition, a snowball system allows school key informants to provide further contact details at the end of the survey. The survey is conducted either as a computer-assisted telephone interview or as an online survey, with participation in the form of a telephone interview being expressly recommended, as participants can ask questions about the complex questionnaire. The survey takes about 35 (+/- 10) minutes. All participants must actively consent to the data collection by checking a box at the beginning of the survey. If possible, all school key informants who participated in the first survey should also participate in the two follow-up surveys. In addition, other persons may also respond to the survey in subsequent waves. To enable the data from the three survey waves to be merged at a later stage, participants generate a personal pseudonym in each survey wave according to the Duisburg Code [22]. No personal data such as real names or personal addresses are collected. The SKI consists of validated multi-item scales on the following topics: (1) local conditions (e.g., opportunities for participation, social climate), (2) intermediate outcomes (e.g., willingness to change, support for SDGs), and (3) sociodemographic data (e.g., gender, age, role at school).

#### School board interview (SBI)

At the beginning of each survey wave, the contact persons at the intervention schools receive a copy of the questionnaire (supplement 7; available in German), a survey link, a privacy policy, and study information for the participants. The contact person distributes the survey link to the FREI DAY team (here referred to as the school board). The school board consists of all persons who are actively involved in the introduction and/or implementation of FREI DAY. The online survey takes about 25 (+/- 10) minutes to complete. All participants must actively consent to the data collection by checking a box at the beginning of the survey. If possible, all school board members who participated in the first survey should also participate in the two follow-up surveys. New team members who joined during the survey period can also participate in the follow- up survey.

To enable the data from the three survey waves to be merged at a later stage, participants generate a personal pseudonym in each survey wave according to the Duisburg Code [22]. No personal data such as real names or personal addresses are collected. The SBI consists of validated multi-item scales and collects data on (1) facilitating factors (e.g., team spirit, goal orientation, efficiency, (2) inhibiting factors (e.g., team conflicts, implementation barriers, stress), (3) health economic factors (e.g., motivation, effort), and (4) sociodemographic data (e.g., gender, age, function at the school). In addition, participants can provide contact information for potential school key informants for the SKI. Individuals who have participated in the SBI can also participate in the SKI.

#### Milestones and Benchmarks Tool (MBT)

In each survey wave, the FREI DAY coordinators at the intervention schools receive the MBT from SiA, in which they answer questions about the current status of FREI DAY implementation (supplement 8; available in German). The MBT is embedded within a SiA-survey that asks questions about the implementation of FREI DAY at the school. The data is then transmitted to MHH. The survey takes about 45 min to complete. No personal data, such as real names or personal addresses, is collected. The MBT is based on the instrument used in Communities That Care [23] and measures which of the eight FREI DAY phases the schools have already completed and how difficult the implementation was.

#### Focus groups

A total of two focus groups with 3–8 students will be conducted at approximately 15–20 primary and secondary schools that are implementing FREI DAY. A key criterion is therefore the schools’ existing experience in implementing FREI DAY. In the case of secondary schools, care will also be taken to ensure that numerous types of schools are included in the survey: secondary modern schools, integrated comprehensive schools, cooperative comprehensive schools, grammar schools, and vocational schools. The discussion will begin with photo motifs related to FREI DAY selected and commented on by the students themselves, based on the photovoice method [24]. The subsequent discussion focuses on evaluating FREI DAY, learning support, and the learning environment (supplement 9 + 10; available in German). Students describe what FREI DAY means to them offering insights from both academic and personal perspectives. In addition, the opportunities and obstacles of working together in project groups under teacher guidance are examined. Students also discuss how much they and their projects consider sustainability, and the contribution they make to implementing the SDGs in their region. This ties in with transformative action in the context of ESD 2030. Finally, a brief review refers back to the photo motifs selected at the beginning and is combined with a concluding flash round.

#### Teacher interviews

Up to two teacher interviews will be conducted at each of the 10–15 schools implementing FREI DAY. A key criterion for selecting teachers is that the schools have had extensive experience in implementing FREI DAY (at least two school years). In addition, attention is paid to ensuring the greatest possible diversity of school types in order to reflect a broad spectrum of school realities with regard to FREI DAY. The interview focuses on analysing the changes initiated by FREI DAY, examining four areas based on the four quadrants of integral theory [25] – internal, external, collective, and individual (supplement 11; available in German). The questions asked include what structural and organizational conditions were created for FREI DAY and to what extent regional networks are forming based on the school (collective, external). The change in the school community and the relationship between students and teachers, as well as the emergence of a possible culture of sustainability in the school, are also focused on (collective, internal). In addition, the study will examine how FREI DAY has changed the professional attitude toward teaching and learning and how teachers and their students view the future (individual, external). It will also look at how the introduction of FREI DAY has changed teachers’ personal workloads, their relationship to their profession, and their individual values (individual, internal). Subsequently, the individual understanding of the transformation in the school through FREI DAY is examined. For this purpose, the aspects mentioned above are used graphically to weigh up where such a transformation process begins from the teachers’ point of view and how it develops. Finally, each teacher is asked to justify their feelings about FREI DAY by selecting one or more blobs from a blob tree.

#### Most significant change

The Most Significant Change (MSC) method [26, 27] is implemented in schools that participate in FREI DAY. Different types of schools should be taken into account here. However, as the method is very time-consuming and is planned to be repeated at the respective schools, a maximum of five schools can participate. The MSC method consists of three core elements (supplement 12; available in German): (1) School members write stories about significant changes they have noticed at their schools as a result of FREI DAY. Using worksheets (in school classes) (supplement 13; available in German) and a SoSci-Survey questionnaire (for adults – e.g., parents, teachers, FREI DAY coordinators, school administrators), participants describe what they believe was the most significant change brought about by FREI DAY. The process of recalling the events of FREI DAY, deciding on a change, and writing the story takes 10–60 min, depending on the participant. (2) Once enough stories have been collected, a discussion panel moderated by the study team lasting several hours will be held, consisting of various members of the school community. The panel will read a selection of the stories and discuss which changes are considered particularly significant, drawing conclusions for the further development of FREI DAY. The panel discussion will be recorded and evaluated. (3) In the final step, the stories and the results from the discussion panel will be shared with the school community in the form of a report and/or a school exhibition. The school can decide for itself whether and to what extent it would like to take up the suggestions for school development. If there is interest, the MSC method will be implemented again at the school after one year, possibly in a slightly modified form with regard to the prompts (worksheets, questionnaires) for writing stories. In this way, the method will also be further developed with feedback from the schools. The ultimate goal is to use the transfer strategy to provide suggestions on which prompts have led to “good” stories and how schools could implement the MSC method independently, if necessary, in order to evaluate FREI DAY or other innovative formats and improve them within the school’s internal framework.

### Outcomes

#### Primary student-level outcomes

To evaluate the health-promoting effects of FREI DAY, a specially developed student questionnaire is used to examine well-being [28], optimism [29], and general self-efficacy expectations [30] using validated multi-item scales ranging from 1 = disagree to 4 = agree. The scale for fear of the future is based on a study by the Federal Ministry for the Environment, Nature Conservation, and Nuclear Safety and the Federal Environment Agency [31] and is a multi-item scale ranging from 1 = very afraid to 4 = not afraid. In addition, the effects of FREI DAY on ecological sustainability (environmental awareness [32, 33], awareness of environmental consequences [34], attribution of responsibility for environmental problems [34]) are measured using the response format from 1 = disagree to 4 = agree. For primary school, the items were shortened and formulated in simpler terms.

#### Secondary student-level outcomes

As secondary outcomes social and emotional skills (cooperation and discussion skills [35], self-efficacy expectations and social skills [36], perspective taking [37], empathy [35]) and cognitive skills (creative thinking [35], problem-solving skills [35], metacognition – planning and structuring [38]) were recorded. For primary school, the items were shortened and simplified. In addition, the metacognition scale was removed for primary school students. Furthermore, as secondary outcomes, the SKI collects data on a validated construct for student participation, the social climate, and a self-developed indicator for the sustainability climate of the school. The questionnaire for the SKI is an adaptation of the Community Key Informant Interview from the German effectiveness study on Communities That Care [39]. All constructs collected are measured using 4-point multi-item scales (1 = disagree to 4 = agree).

#### Intermediate school-level outcomes

These outcomes are considered intermediate school-level outcomes because they are expected to mediate or facilitate longer-term effects of FREI DAY on students. As part of the school key informant interview, we measure, as intermediate outcomes, whether the intervention schools achieve significantly greater improvements in the dimensions of school capacity for transformation compared to the comparison schools during the observation period. To this end, 4-level multi-item scales from a validated instrument for measuring community capacity for health promotion [40, 41] are adapted and modified to the school context.

### Sample size calculation

In summary, the study aims to recruit a gross sample of at least 60 schools. This corresponds to an expected net sample of at least 54 schools for the system-change outcomes and, depending on the achieved student response and follow-up rates, to at least 720 students in 36 schools for the minimum student-level analysis or approximately 1,080 students in 54 schools if the target net school sample is reached. Sample size calculation for longitudinal observational studies with clustered samples is so complex that, to date, only a few sample size calculators offer solutions for this, and even then, not for all design specifications. This is because there are still no proven solutions for some design specifications. This also applies to this project, which is why we can only aim for the best possible approximation of a suitable case number calculation. The following case number calculations were performed using an online tool from the National Institutes of Health (https://researchmethodsresources.nih.gov). This tool enables a highly differentiated sample size calculation for stepped wedge group randomized trials (SWGRTs) and, in our opinion, is currently the best approximation for the case number calculation required for our study design.

#### Intermediate school-level outcomes

As system change outcomes, five dimensions of school capacity for transformation are assessed at three points in time using the SKI. For the calculation, we had to treat the surveys as repeated cross-sections, but it is actually an open cohort. For the system change outcomes, we expect moderate effects (d = 0.5), which we want to detect with a type 1 error rate of 5% and a statistical power of 80%. For the system change outcomes, we also assume an intracluster correlation (ICC) = 0.1 and a cluster autocorrelation (CAC) = 0.5 [42]. The reliability of context characteristics measured using intersubjective ratings correlates strongly and positively with the number of raters/respondents [43]. Studies measuring community characteristics have shown that a sample size of ten respondents per community is approximately the lower limit for reliable measurements [39]. The calculation is also based on the assumption that the analyses are performed using mixed models and are therefore adjusted for cluster membership and time/sequence. We see no need to adjust further covariates at the cluster level. At the individual level, adjustments should be made to the baseline values of the dependent variable. Here, we assume that the baseline values explain 0.25 of the variances in the post-measurement of the dependent variable. This results in a required sample size of nine schools with ten respondents per sequence. With the postulated three sequences, this corresponds to a total of 27 schools. In order to be able to perform sensitivity analyses separately for subgroups (e.g., degree of deprivation of the school location), it should be possible to differentiate between at least two subgroups with at least 27 schools each. This would require a net sample of at least 54 schools. Assuming a loss to follow-up of 10%, the gros sample would have to comprise at least 60 schools if subgroup analyses are to be possible.

#### Primary and secondary student-level outcomes

With the student survey, we collect outcomes of health promotion and sustainable development (e.g., resilience, self-efficacy, sense of responsibility, environmental awareness) at three points in time. The student survey is designed as a closed cohort. For these outcomes, we expect weak effects (d = 0.2), which we want to detect with a type 1 error rate of 5% and a statistical power of 80%. For these measurements, we assume intracluster correlations (ICC) = 0.04, individual autocorrelations = 0.7, and cluster autocorrelations (CAC) = 0.5 [42]. We also assume that at least 60 students per school will initially participate in the student survey on average, but that a high loss to follow-up at this level could result in a net sample of only 20 students per school on average. The calculation is also based on the assumption that the analyses will be performed using mixed models and will therefore be adjusted for cluster membership and time/sequence. We see no need to adjust for further covariates at the cluster level. At the individual level, adjustments should be made to the baseline values of the dependent variable. Here, we assume that the baseline values explain 0.5 of the variance in the post-measurement of the dependent variable. This results in a required sample size of 12 schools with ten respondents per sequence. With the postulated three sequences, this corresponds to a total of 36 schools. This corresponds to a minimum of (36 schools x 20 students) 720 students. If we achieve the target net sample of 54 schools, at least 1,080 students could be analysed. This would allow for limited sensitivity and subgroup analyses (e.g., by gender or socioeconomic status of the adolescents) by including interaction terms in the mixed models.

### Statistical methods

Missing values are defined in advance for all surveys and recoded if necessary. Multiple imputation is performed for the student survey. Mean imputation is performed for the SBI and SKI data. If only one item in the construct was answered, this case remains in the analyses. However, values are only imputed if at least three people from the same school have provided information on the item. If people have systematically not received an item or a scale (e.g., due to a filter question), they are excluded from the imputation. The information is only imputed to the extent that the people have provided information; after a termination, the person is no longer included in the imputation.

In an early phase of the study, the following analyses are planned based on the cross-sectional data from the first wave of surveys: (1) The psychometric properties of the instruments used will be examined. Exploratory and confirmatory factor analyses will be calculated to check structural validity. Cronbach’s α will be calculated for each scale to check reliability. An intracluster correlation coefficient (ICC) will be calculated for all constructs as a measure of interrater agreement. At the school level, this is calculated as the proportion of the total variance attributable to the schools. For an initial assessment of construct validity, correlations between the constructs are analysed. These are carried out at both the individual and school levels, with the latter based on school-specific means. (2) Hierarchical models are used to test hypotheses about correlations between school characteristics and student characteristics, such as whether higher school capacity for transformation is associated with higher levels of health- and environment-related attitudes and behaviours among students. Further analyses examine whether students differ in their health- and environment-related attitudes and behaviours depending on whether they have already completed FREI DAY at least once.

The main effects of the intervention are examined using longitudinal data according to the intention-to-treat principle using mixed models as average treatment effects. Mixed models are calculated for all intermediate as well as for all primary and secondary outcomes.

Subgroup and sensitivity analyses are performed to examine whether the intervention effects vary according to subgroups (1) at the school level (e.g., school type, deprivation of the location, willingness of the school to innovate), (2) at the level of the FREI DAY team and learning guides (e.g., attitude, motivation, effort, implementation quality), and (3) at the individual level (e.g., socioeconomic status or gender of adolescents). To this end, all models are calculated separately for each subgroup. On the other hand, effect moderators at the individual level are identified using interaction terms.

From a health economics perspective, we examine how the motivation and effort of the learning guides influence the effectiveness of the intervention at the student level. To this end, we conduct multivariate regression analyses and a mediation analysis.

## Discussion

This is the first study to evaluate the effectiveness of the whole school approach FREI DAY. The results of the natural experiment and qualitative research will be triangulated using a mixed methods design. To this end, the SDGs, the concepts of life skills, shaping competence and planetary health form the theoretical framework for bringing together the research results on health promotion, environmental sustainability and health inequality. This theoretical framework makes it possible to interpret the research results in their situational and overall contexts using the sociological concept of explanatory understanding. Our primary aim is to gain a deeper understanding of the mechanisms of action of FREI DAY and the role of local conditions in its implementation. We expect FREI DAY to improve students’ well-being, life skills, environmental awareness, and environmental responsibility. In addition, it is expected to strengthen the transformative capacity of schools, create a new culture of learning and sustainability, and strengthen collective resilience.

Several limitations should be considered when interpreting the findings of this study. First, the study uses a natural experiment with a non-randomised stepped-wedge structure. Schools decide themselves whether and when to implement FREI DAY, which may introduce selection bias and confounding by school characteristics such as innovation readiness, leadership commitment, or prior engagement with education for sustainable development. Although mixed models and adjustment for relevant covariates will be applied, residual confounding cannot be fully excluded. Second, FREI DAY is a complex whole-school intervention whose implementation is expected to vary considerably across schools with regard to intensity, duration, organisational embedding, and implementation fidelity. Such heterogeneity may contribute to variation in intervention effects. Third, most outcomes rely on self-reported data from students and school stakeholders and may therefore be influenced by social desirability, recall bias, and common-method variance. Finally, attrition over the three survey waves may reduce the available longitudinal sample and potentially affect representativeness despite planned strategies for participant retention and the use of statistical methods to address missing data. Nevertheless, the naturalistic design enhances external validity by allowing the evaluation of FREI DAY under real-world implementation conditions.

## Supplementary Information

Supplementary Material 1

Supplementary Material 2

Supplementary Material 3

Supplementary Material 4

Supplementary Material 5

Supplementary Material 6

Supplementary Material 7

Supplementary Material 8

Supplementary Material 9

Supplementary Material 10

Supplementary Material 11

Supplementary Material 12

Supplementary Material 13

## Data Availability

No datasets were generated or analysed during the current study.

## Abbreviations

CAC: Cluster Autocorrelation
ESD: Education for Sustainable Development
HPS: Health Promoting School
ICC: Intracluster Correlation
MBT: Milestones and Benchmarks Tool
MHH: Hannover Medical School
MSC: Most Significant Change
RLSB: Regional Office for School and Education (ger.: Regionale Landesamt für Schule und Bildung)
SBI: School Board Interview
SDG: Sustainable Development Goal
SiA: Schule im Aufbruch
SKI: School Key Informant Interview
SWGRT: Stepped Wedge Group Randomized Trial
UNESCO: United Nations Educational, Scientific and Cultural Organization
WHO: World Health Organization
