# Supplementary material for: Study protocol for a quasi-experimental evaluation of the whole school approach FREI DAY
Source: Arch Public Health. 2026 Oct 13;84:222. doi: 10.1186/s13690-026-02088-7 (PMC13670517; doi:10.1186/s13690-026-02088-7)
Supplement: Supplementary file 1 [file 13690_2026_2088_MOESM10_ESM.pdf]

Supplement 10: Guide for focus groups in secondary schools

Leitfaden Fokusgruppendiskussion

Zeit ca. 1h	Fragestellungen	Zusätzliche Impulse	Material
4 Min.	**1. Einstieg**<br>• Vorstellung von mir und dem Projekt<br>• Hinweise für den Ablauf<br>• Hinweise auf Anonymität und Aufzeichnung der Fokusgruppe<br>• *Vorstellungsrunde* der Schüler:innen (Name, Alter, Klasse)<br><br>*Wie ist der FREI DAY an eurer Schule organisiert?*<br><br>*Seit wann nehmt ihr am FREI DAY teil?*<br><br>*Welches Projekt führt ihr durch?*<br><br>*An welche Nachhaltigkeitsziele (SDGs) knüpfen eure Projekte an?*	Abbildung aller SDGs	Abb.
12 Min.	**2. Partizipativer Einstieg**<br><br>**Reflexive Fotografie**<br>Den Schüler:innen wird folgende Aufgabegestellt:<br>**Was macht für dich den FREI DAY aus?**		iPads des IDN

	Bitte macht ein Foto von dem, was für euch den FREI DAY ausmacht. Ihr könnt zum Beispiel Orte, Gegenstände oder Menschen fotografieren. Falls ihr einzelne Menschen fotografiert, fragt diese bitte vorher, ob die Aufnahme für sie in Ordnung ist. Wenn das, was ihr fotografieren möchtet, nicht auf dem Schulgelände erreichbar ist, könnt ihr auch ein Bild aus dem Internet raussuchen. Achtet jedoch darauf, sensibel mit den Fotos anderer umzugehen.		
10 Min.	**3. Reflexionsfragen zu den aufgenommenen Fotos**<br><br>*Bevor ihr euch gegenseitig eure Bilder präsentiert, überlegt jede:r für sich eine Begründung für die Auswahl des Motivs.*<br><br>*Zeigt bitte euer Bild und erläutert, warum ihr euch für dieses Motiv entschieden habt.*<br><br>*Welche Gefühle oder Erlebnisse verbindet ihr mit dem Motiv?*	*Sind eure Gefühle im Zusammenhang mit dem Motiv eher positiv, neutral oder eher negativ?*	
10 Min.	**4. Bewertung des FREI DAY**<br><br>*Was unterscheidet für euch den FREI DAY vom normalen Schulunterricht?*<br><br>*Wie gefällt euch der FREI DAY? Bewertet diesen auf einer Skala von 1 (sehr gut) bis 6 (ungenügend)*<br><br>*Was hat sich durch den FREI DAY für euch verändert?*	*Was genau ist anders?*<br><br>*Was fällt euch schwer/ leicht beim FREI DAY?*<br><br>*Geht ihr lieber in die Schule?*	

		Gibt es für euch weniger Stress im Schulalltag?	
7 Min.	**5. Lernklima in der Gruppe/Lernbegleitung**<br><br>*Wie gefällt euch das Arbeiten in einer Projektgruppe?*<br><br>*Wie geht ihr mit schwierigen Situationen bei der Projektarbeit um?*<br><br>*Wie gut fühlt ihr euch von den Erwachsenen bei eurem Projekt unterstützt und warum? Bewertet auf einer Skala von 1 (sehr gut) bis 6 (ungenügend).*	*Was gefällt euch besonders gut/nicht so gut?*<br><br>*Gibt es auch mal Streit oder Meinungsverschiedenheiten? Was macht ihr dann?*<br><br>*Was machen die Lehrkräfte gut/ nicht gut im Rahmen des FREI DAY?*<br>*Hat sich durch den FREI DAY das Verhältnis zu den Lehrkräften verändert? Falls ja, wie begründet ihr das?*	
12 Min.	**6. Transformatives Handeln**<br><br>*Wie berücksichtigen eure Projekte Aspekte der Nachhaltigkeit?*<br><br>*Wie sehr tragen eure Projekte zur Verwirklichung der SDGs in eurer Region bei? Bewerte diese auf einer Skala von 1 (sehr gut) bis 6 (ungenügend)*<br><br>*Hat sich durch den FREI DAY verändert, wie ihr auf die Zukunft schaut? Was traut ihr euch jetzt mehr/weniger zu?*	*Was hat euch konkret bewegt, etwas zu ändern? Gab es*	

		besondere Momente, die euch darin bestärkt haben?	
5 Min	**7. Abschluss**<br><br>*Am Anfang dieser Diskussion habt ihr Fotos gemacht, die zeigen sollten, was für euch den FREI DAY ausmacht. Würdet ihr jetzt, nachdem wir ausführlicher über den FREI DAY gesprochen haben, ein anderes Motiv wählen?*<br><br>Blitzlicht: *Was nehmt ihr aus unserer Diskussionsrunde mit?*	*Falls ja, warum?*	

ZIELE FÜR NACHHALTIGE ENTWICKLUNG

1 KEINE ARMUT

2 KEIN HUNGER

3 GESUNDHEIT UND WOHLERGEHEN

4 HOCHWERTIGE BILDUNG

5 GESCHLECHTER-GLEICHHEIT

6 SAUBERES WASSER UND SANITÄR-EINRICHTUNGEN

7 BEZAHLBARE UND SAUBERE ENERGIE

8 MENSCHENWÜRDIGE ARBEIT UND WIRTSCHAFTS-WACHSTUM

9 INDUSTRIE, INNOVATION UND INFRASTRUKTUR

10 WENIGER UNGLEICHHEITEN

11 NACHHALTIGE STÄDTE UND GEMEINDEN

12 NACHHALTIGE/R KONSUM UND PRODUKTION

13 MASSNAHMEN ZUM KLIMASCHUTZ

14 LEBEN UNTER WASSER

15 LEBEN AN LAND

16 FRIEDEN, GERECHTIGKEIT UND STARKE INSTITUTIONEN

17 PARTNER-SCHAFTEN ZUR ERREICHUNG DER ZIELE

ZIELE FÜR NACHHALTIGE ENTWICKLUNG

Developed in collaboration with TROLLBÄCK+COMPANY | TheGlobalGoals@trollback.com | +1.212.529.1010
For queries on usage, contact: dpicampaigns@un.org | Non official translation made by UNRIC Brussels (September 2015)

Medizinische Hochschule Hannover

Leibniz Universität Hannover
