# Supplementary material for: Study protocol for a quasi-experimental evaluation of the whole school approach FREI DAY
Source: Arch Public Health. 2026 Oct 13;84:222. doi: 10.1186/s13690-026-02088-7 (PMC13670517; doi:10.1186/s13690-026-02088-7)
Supplement: Supplementary file 2 [file 13690_2026_2088_MOESM11_ESM.pdf]

Supplement 11: Guide for teacher interviews

Leitfaden Lehrkräfteinterview

Zeit 45 Min.	Fragestellungen	Zusätzliche Impulse	Material
12 Min.	**1. Einstieg**<br>• Vorstellung von mir und dem Projekt<br>• Hinweise auf Anonymität und Aufzeichnung des Interviews<br><br>*Seit wann sind Sie Lehrkraft?*<br><br>*Welche Fächer unterrichten Sie?*<br><br>*Was kommt Ihnen als erstes in den Sinn, wenn Sie an den FREI DAY denken?*<br><br>*Wie ist der FREI DAY an Ihrer Schule organisiert?*<br><br>*Seit wann sind Sie im FREI DAY aktiv beteiligt und was hat Sie dazu motiviert?*<br><br>*Wie begleiten Sie die SuS bei Ihrer Projektarbeit?*<br><br>*Welche abgeschlossenen Projekte sind Ihnen noch in besonderer Erinnerung?*		

	Haben Sie einen Schlüsselmoment in Bezug auf den FREI DAY, d.h. ein Erlebnis, das sie besonders nachhaltig berührt hat oder Ihnen ganz besonders im Gedächtnis geblieben ist?		
5 Min.	**2. Äußere Strukturen einer nachhaltigen Lernkultur (kollektiv, systemisch)**<br><br>*Welche organisatorischen Veränderungen wurden vorgenommen, um den FREI DAY an Ihrer Schule umzusetzen?*<br><br>*Gibt es nach wie vor an Ihrer Schule Rahmenbedingungen, die die Umsetzung des FREI DAY erschweren?*<br><br>*Wie sehr bilden sich durch den FREI DAY regionale Netzwerke?*<br><br>*Wie sehr tragen die FREI DAY-Projekte zur Verwirklichung der SDGs im regionalen Umfeld der Schule bei?*	*Regionale Zusammenarbeit mit Firmen, Vereinen, außerschulischen Lernorten?*	Abb. 1 SDGs
5 Min.	**3. Innere Strukturen einer nachhaltigen Lernkultur (kollektiv, kulturell)**<br><br>*Was hat sich durch den FREI DAY in Ihrer Schule und Schulgemeinschaft verändert?*<br><br>*Inwiefern trägt der FREI DAY zu einer Nachhaltigkeitskultur Ihrer Schule bei?*<br><br>*Inwiefern hat sich durch den FREI DAY das Verhältnis zwischen Lehrkräften und SuS verändert?*		

5 Min.	**4. Äußere Strukturen einer nachhaltigen Lernkultur (individuell, kulturell)**<br><br>*Wie hat sich Ihre professionelle Haltung zum Lehren und Lernen durch den FREI DAY verändert?*<br><br>*Welche Handlungsmuster oder Routinen haben Sie im Schulalltag durch den FREI DAY entwickelt?*<br><br>*Hat sich durch den FREI DAY verändert, wie Sie grundsätzlich mit Ihren SuS auf die Zukunft schauen?*		
5 Min.	**5. Innere Strukturen einer nachhaltigen Lernkultur (individuell, persönlich)**<br><br>*Was hat sich für Sie persönlich durch die Einführung des FREI DAY geändert?*<br><br>*Wie hat sich Ihre Arbeitsbelastung durch den FREI DAY verändert?*<br><br>*Wie hat sich die Beziehung zu Ihrem Beruf durch den FREI DAY verändert?*<br><br>*Welche Haltungen, Werte und Überzeugungen werden für Sie durch den FREI DAY besonders angesprochen oder herausgefordert?*	*Mit welchen Herausforderungen kämpfen Sie im Schulalltag des FREI DAY?*<br><br>*Gibt es mehr oder weniger Stress im Schulalltag?*	

7 Min.	**6. Transformationsprozesse**<br><br>*Was verstehen Sie unter einer Transformation von Schule im Allgemeinen?*<br><br>*Wir haben zuvor über innere, äußere, kollektive und individuelle Aspekte einer Lernkultur im Kontext des FREI DAY gesprochen. Die Abb. verdeutlicht diese Strukturen.*<br><br>*Wie gewichten Sie die vier Felder (mit ihren jeweiligen Perspektiven auf Veränderung) für den Transformationsprozess der Schule auf Grundlage Ihrer Erfahrungen mit dem FREI DAY? Wo startet ein solcher Transformationsprozess? Bringen Sie die vier Felder in eine zeitliche Abfolge.*	*Was bedeutet für Sie eine Entwicklung/ Veränderung von Schule/ ein Wandel?*<br><br>*Malen Sie Pfeile in den Kreis oder tragen Nummern in diesen ein.*	Abb. 2 Quadranten
5 Min.	**7. Blob Tree**<br><br>*Ich möchte Ihnen nun einen Blob Tree, eine Baumgrafik, zeigen, auf der sich unterschiedliche Blob-Figuren befinden. Diese Figuren stellen verschiedene Emotionen, Körperhaltungen und soziale Gefüge dar.*<br><br>*Welche der abgebildeten Blobs beschreibt ihre Gefühle und Gedanken in Bezug auf den FREI DAY am besten und warum?*	*Welcher Blob macht für Sie den FREI DAY aus?*	Abb. 3 Blob Tree
1 Min.	**8. Abschluss**<br><br>*Haben Sie noch etwas mitzuteilen im Zusammenhang mit dem FREI DAY, über das wir noch nicht gesprochen haben?*		

Vier Perspektiven auf

ZIELE FÜR NACHHALTIGE ENTWICKLUNG

schulische

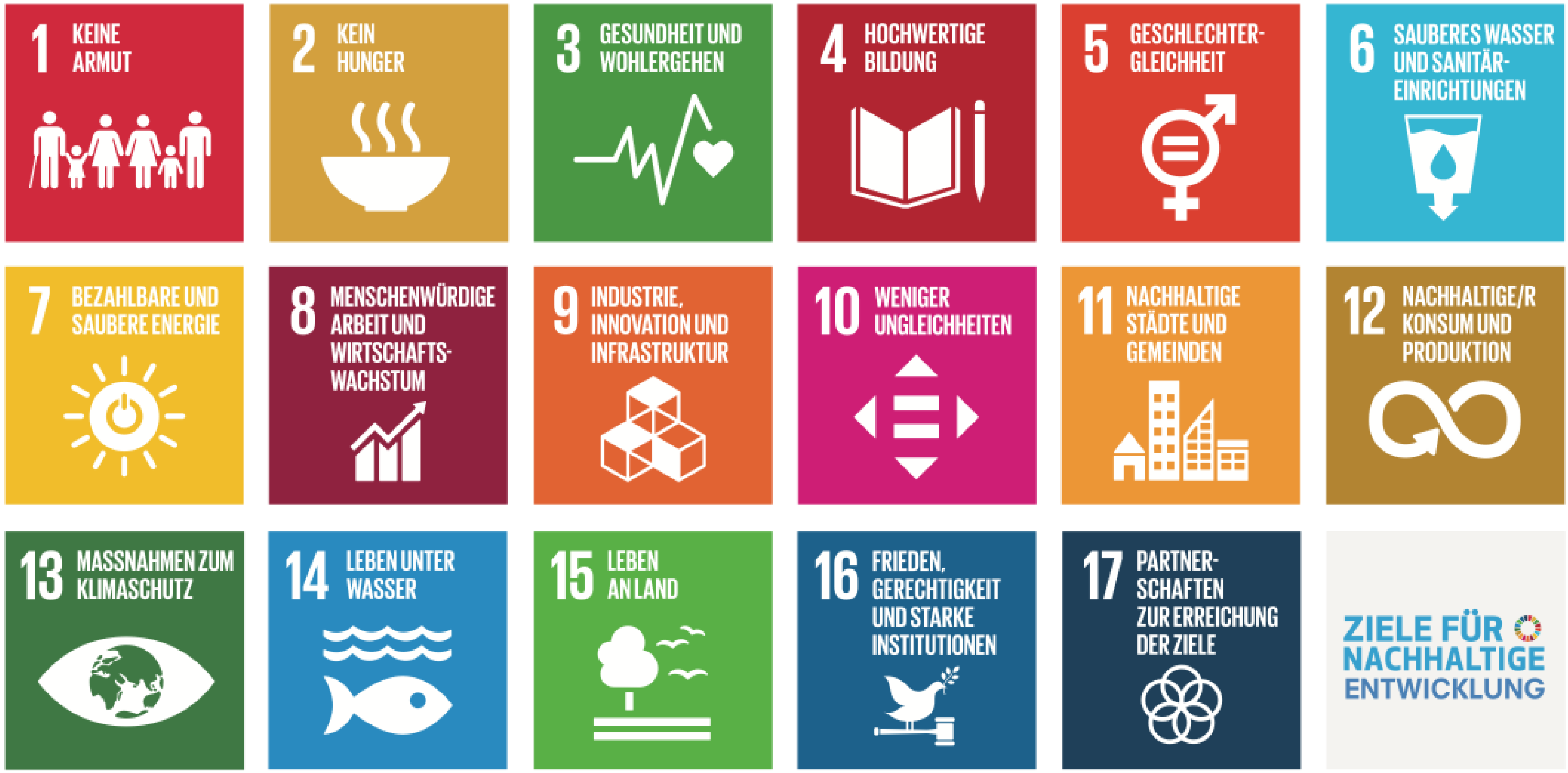

Transformation

Developed in collaboration with TROLLBÄCK + COMPANY | TheGlobalGoals@trollback.com | +1.212.529.1010
For queries on usage, contact: dpicampaigns@un.org | Non official translation made by UNRIC Brussels (September 2015)

Leibniz Universität Hannover
Medizinische Hochschule Hannover

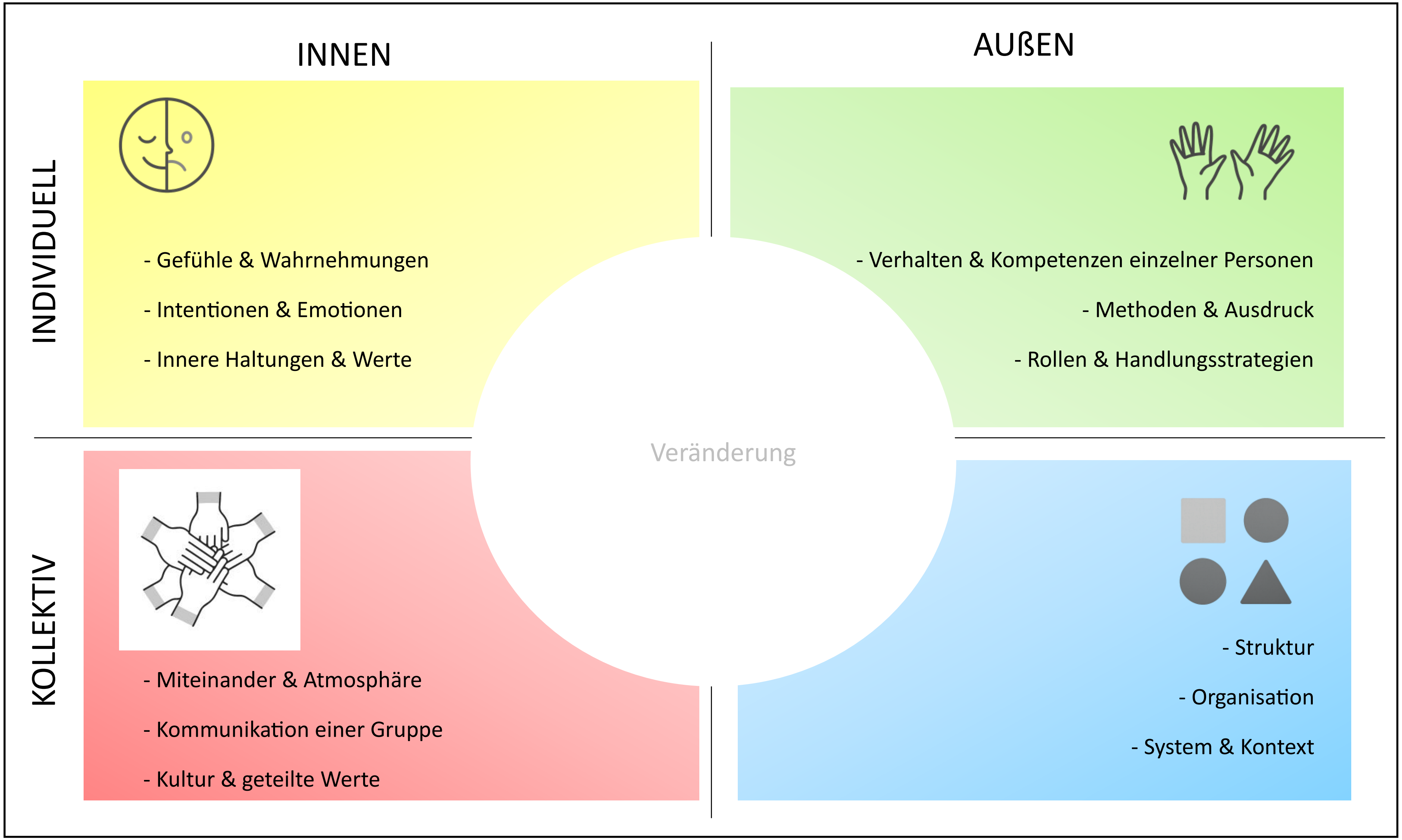

Leibniz Universität Hannover

Medizinische Hochschule Hannover

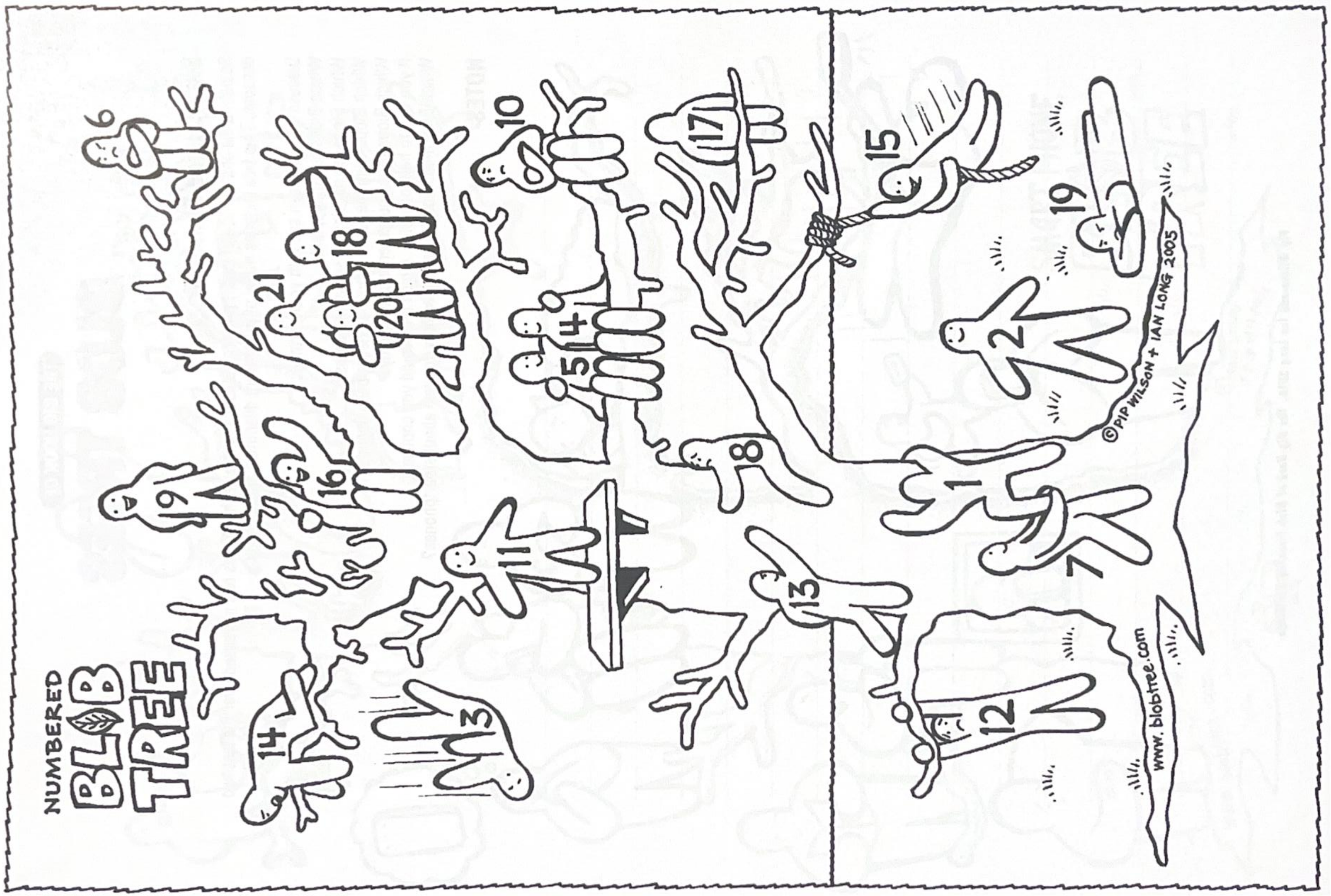
NUMBERED
BLOB TREE
©PIP WILSON + IAN LONG 2005
www.blobtree.com
