# Supplementary material for: Study protocol for a quasi-experimental evaluation of the whole school approach FREI DAY
Source: Arch Public Health. 2026 Oct 13;84:222. doi: 10.1186/s13690-026-02088-7 (PMC13670517; doi:10.1186/s13690-026-02088-7)
Supplement: Supplementary file 3 [file 13690_2026_2088_MOESM12_ESM.pdf]

Supplement 12: Planning and agenda for the *Most Significant Change* discussion panel

Planung und Ablauf des MSC-Diskussionspanels

Bedenken und Frage nach Feedback/ Ideen:

- Ablauf braucht viel Zeit. Passt zur Dauer, die laut MSC-Expert:innen mindestens berechnet werden sollte. Für Schulalltag ist das aber sehr lang.
  -> Kürzen, z.B. indem die Zahl der Geschichten reduziert wird? Habe die Zeiten bezogen auf sehr kurze Geschichten ausgerechnet (die Geschichten an der Montessori-Schule bestehen meist nur aus 3 – 6 Sätzen). Bei längeren Geschichten (z.B. KGS Pattensen) würde ich max. 5 Geschichten einbringen, damit TN sich auf alle konzentrieren können.
  Sollte Anzahl der Geschichten trotzdem auch bei kurzen Geschichten reduziert werden? -> MHH-Team sagt, das wäre gute Stellschraube
- Phase 2 – Geschichten lesen und besprechen: ich stelle mir Version besser vor, in der auch in Kleingruppen vorgearbeitet wird (siehe Ablauf). Was denkt ihr? Welche Nachteile sollten dabei beachtet werden und wie könnte man damit umgehen?
- Freue mich über alle Ideen und Ergänzungen! z.B. Gedanken zum Ablauf; Ideen dazu, wie Gespräche in der großen Gruppe gut aufgezeichnet werden können, auch wenn TN sich im Raum bewegen, etc?

Teilnehmende und Anzahl Geschichten:

- ca. 5 - max. 8 Geschichten; wahrscheinlich ca. 6
- 8 - 10 Teilnehmende (Schüler:innen, Lehrkräfte, Schulleitung, FREI DAY Koordination)
- Wenn Geschichten in Kleingruppen gelesen werden:
  - o drei/vier Gruppen mit 2-3 Personen?
  - o pro Kleingruppe 1-2 Geschichten

Material:

- Getränke, Kekse, Gummibärchen, Nüsse
- Rest siehe Ablauf

Zu beachten währenddessen:

- Machtdynamiken und Hierarchie: SuS besonderes Rederecht geben? So wie es das manchmal gibt als Redeliste für FLINTA* Personen?
  - o Mit SuS vorbesprechen, was sie brauchen um sich sprechfähig zu fühlen

Ziele

Ablauf

Zeit	Inhalt	Material, Anmerkung
vorher	**Flipcharts** (Bild Fenster, Ablauf, Gesprächsrahmen, Ideenhafen) aufhängen oder auslegen<br>**Aufnahmegeräte** - Check und bereitlegen<br>**Kreppband** für Namensschilder;<br>**Materialtisch** mit dünnen Stiften, Markern, Papier, Modikarten;<br>**Geschichten**<br>**Modinotizen**; ZIMM<br>**Leitfragen** auf Modikarten<br>**Musikbox**	
0:00 – 0:15	**1) Einleitung und Rahmen**<br>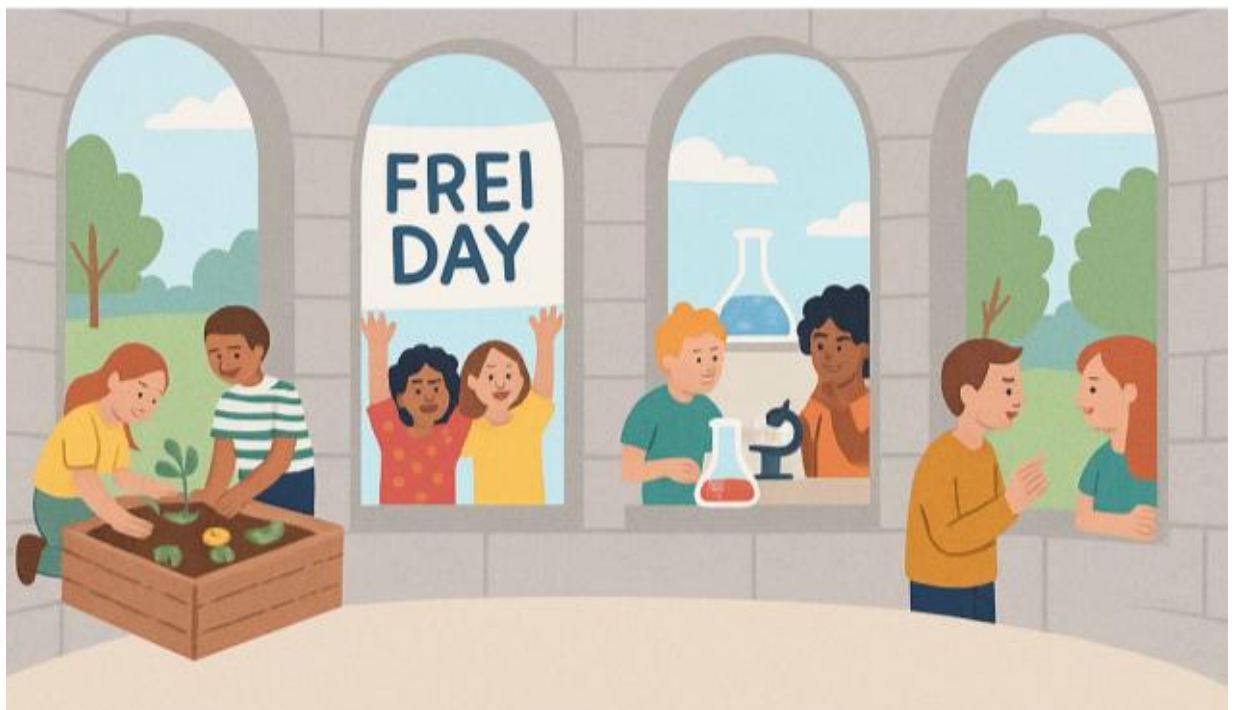<br>Wir alle schauen auf den FREI DAY mit unterschiedlichen Perspektiven, wie durch unterschiedliche Fenster. Führt zu verschiedenen Sichtweisen. Gesammelte Geschichten bieten verschiedene Perspektiven darauf, welche wichtigen Veränderungen durch FREI DAY passieren. Uns interessiert, welche Perspektive ihr besonders wichtig für Weiterentwicklung des FREI DAY findet! Was ihr wichtig findet, hängt von eurer Rolle und persönlichem Hintergrund ab, davon durch welches Fenster ihr blickt. Ziel ist, dass andere von euch lernen können, was euch besonders wichtig ist und warum. Deshalb gerne persönlich - was müssen andere wissen, um euch gut zu verstehen? Warum findet ihr welche Veränderung besonders wichtig?	Aufzeichnen!<br><br>Vorstellen, Zweck/womit gehen wir raus, MSC erklären, Ablaufplan auf Flipchart zeigen; Dauer ca. 2,5 Stunden; Meldezeichen. Bedürfnisse?<br><br>Aufstellungsspiel um alle kurz ins Sprechen zu bringen, Gruppe miteinander ankommt, Hürden senkt [1]<br><br>Bild 1: FREI DAY Fenster<br>Flipchart 2: Ablauf Panel<br>Flipchart 3: Gesprächsrahmen Ideenhafen
0:15 – 0:30	**2) Geschichten vorlesen und besprechen**<br>**2a Kleingruppenarbeit** 15mins	

	- 6 Geschichten auf Gruppen verteilt. Vorlesen pro Geschichte braucht ca. 1min, besprechen 5 – 8 Minuten<br>- Vorarbeit in Kleingruppen: lesen; auf Modikarte schreiben, was sich verändert hat, Stichpunkte zum Inhalt;<br>- auswählen wer vorliest in Großgruppe<br><br>Leitfragen zum Besprechen:<br>- Welche (bedeutsame) Veränderung wird beschrieben?<br>- Was macht diese Veränderung bedeutsam?<br>- Was versucht uns die erzählende Person ggf. noch mitzuteilen?	Geschichten ausgedruckt auf Papier<br><br>Modikarten, Marker<br><br>Leitfragen-Modikarten (zu 2)
0:30-0:50	**2b Großgruppe** 15mins<br>- Großgruppe: Jede Gruppe liest Geschichte vor, legt Modikarten aus; fragt welche Veränderungen ergänzt werden sollen (+ wenn Zeit ist was Geschichte auslöst)	Aufzeichnen!<br><br>Impuls geben: gut zuhören, später positionieren zu Perspektive, die du besonders wichtig findest!
0:50 – 0:55	**3a) Gallery-Walk.** Geschichten und zugehörige Modikarten werden im Raum ausgelegt. TN laufen durch Raum, lesen Geschichten, positionieren sich bei Geschichte, die sie besonders bedeutsam finden.<br>- Leitfragen 5: Von all diesen Veränderungen, welche findest du besonders bedeutsam? Warum findest du sie besonders bedeutsam?	Modikarte-Leitfragen zu 3<br><br>Besonders bedeutsam oder besonders bedeutsam für Schulentwicklung?
0:55 – 1:20<br><br>+ 5 falls Geschichten besprochen werden, bei denen niemand steht<br><br>+ Puffer, falls eine Person nochmal Platz wechselt und	**3b) Austausch:**<br>- Warum habe ich mich zu dieser Geschichte gestellt? (2 mins/Person * 9 Personen = 18 Minuten)<br>- Wenn alle gesprochen haben: möchtest du kommentieren, was gesagt wurde, deine Perspektive hinzufügen?<br>~~- Geschichten die nicht besetzt wurden: Was würde eine Person sagen, die bei dieser Geschichte steht, warum sie die Geschichte besonders bedeutsam findet? (+ 5mins)~~<br>- Am Ende fragen: „Hat sich verändert, was ihr besonders bedeutsam findet,	Aufzeichnen!<br>Leere Modikarten oder Papier für Satz<br><br>Erst unkommentiert zuhören; ihr dürft später kommentieren -> Notizen machen wer will<br><br>Ihr dürft jederzeit eure Position in dieser Phase verändern

etwas sagen will	jetzt wo ihr die Gründe der anderen gehört habt? Wechselt ggf. eure Position.“<br>- Jede Person schreibt einen vollständigen Satz auf, warum sie Geschichte besonders bedeutsam findet (+5mins) (markieren zu welcher Geschichte das gehört + Rolle!)	Oder sollte nicht aufgeschrieben werden, um die Zeit zu kürzen (Aufschreiben wird von MSC-Kurs empfohlen)<br><br>mitteilen: Sätze mitaufhängen + auswerten später<br><br>Notizen einsammeln
1:20 - 1:35	**Pause** 10mins kommunizieren, 15 draus machen	
**1:35 – 2:35 + Open End**	**4) Schulentwicklung und Reflexion der Diskussion:** was lernen wir aus Geschichten und der Diskussion? Konsequenzen?<br><br>Think-Pair-Share erklären<br><br>~~Was erzählen uns diese Geschichten darüber, wie es der Schule mit dem Erreichen der Wünsche und Ziele zum FREI DAY geht?~~	Leitfragen-Modikarten Think-Pair-Share
1:35 - 1:50<br><br>15mins (12 kommunizieren + in Wecker einstellen)	**Think:**<br>Fühlen: Wie geht es dir jetzt und welche Gefühle haben die Geschichten und der Austausch bei dir ausgelöst?<br>Denken: Welche Gedanken hat der Austausch bei dir angestoßen? Was nimmst du mit?<br>Handeln: Was ist dein nächster Schritt oder dein Vorschlag für nächste Schritte der Schule?<br><br>Nur wenn Zeit ist:<br>Welche Geschichten wurden vielleicht nicht erzählt, welche Perspektiven nicht gehört?	Notizen – Hinweis: andere Schulmitglieder bekommen Notizen nicht zu sehen. Ich werte die für die Studie aus, anonymisiert. Schreibt eure Rolle drauf, zB Schülerin<br><br>Material: Blatt für Notizen; Stifte; Leitfragen 5 Think
1:50 - 2:05<br><br>15mins (12 kommunizieren + in Wecker einstellen)	**Pair - 4er Gruppen:** Nenne die 2 wichtigsten Punkte, die du aufgeschrieben hast. Fasst die Punkte zusammen und schreibt zu jedem zusammengefassten Punkt einen ganzen Satz auf eine Moderationskarte	Modikarten, Eddings<br><br>Leitfrage 7 Pair<br><br>Notizen einsammeln!
10mins<br><br>2:05 - 2:15	**Share:** In Großgruppe werden die zentralsten Punkte kurz geteilt.	Aufnehmen!<br><br>mitteilen: danach noch Zeit für Umsetzung

15mins<br><br>2:15 - 2:30	**Action:**<br>Entstehen aus den Geschichten oder der Diskussion...<br>- Punkte, die an anderer Stelle weiter besprochen werden sollten?<br>- Aufgaben, die verteilt werden sollten?	Aufnehmen!<br>Punkte mitschreiben, damit TN Überblick behalten was gesagt wurde (entweder ich oder verteilen an TN, damit ich gut weitermoderieren kann)<br><br>Leitfrage 8: Action
5mins<br><br>2:30 - 2:35	**5) Meta-Reflexion zur Methode** MSC: War die Methode hilfreich für euch? Was würdet ihr gleich lassen, was anders machen?	Aufnehmen!

Einleiten

Ich stelle mich vor:

- Jacky, arbeite an Leibniz-Uni in Hannover in Projekt, das FREI DAY erforscht; außerdem gebe ich manchmal Workshops und unterstütze Leute dabei, ins Gespräch zu kommen und für gemeinsame Ziele einzutreten (wie bei euch, FREI DAY Sichtweisen auf FREI DAY sammeln und FREI DAY weiterentwickeln)
- Meine Rolle: Rahmen geben, damit ihr euch gut austauschen könnt - Redeanteile; Rolle als Forscherin – Aufnahme der Diskussion etc
- Workshop-Du

Bild 1 –FREI DAY Fenster. Zweck/womit gehen wir raus + MSC erklären

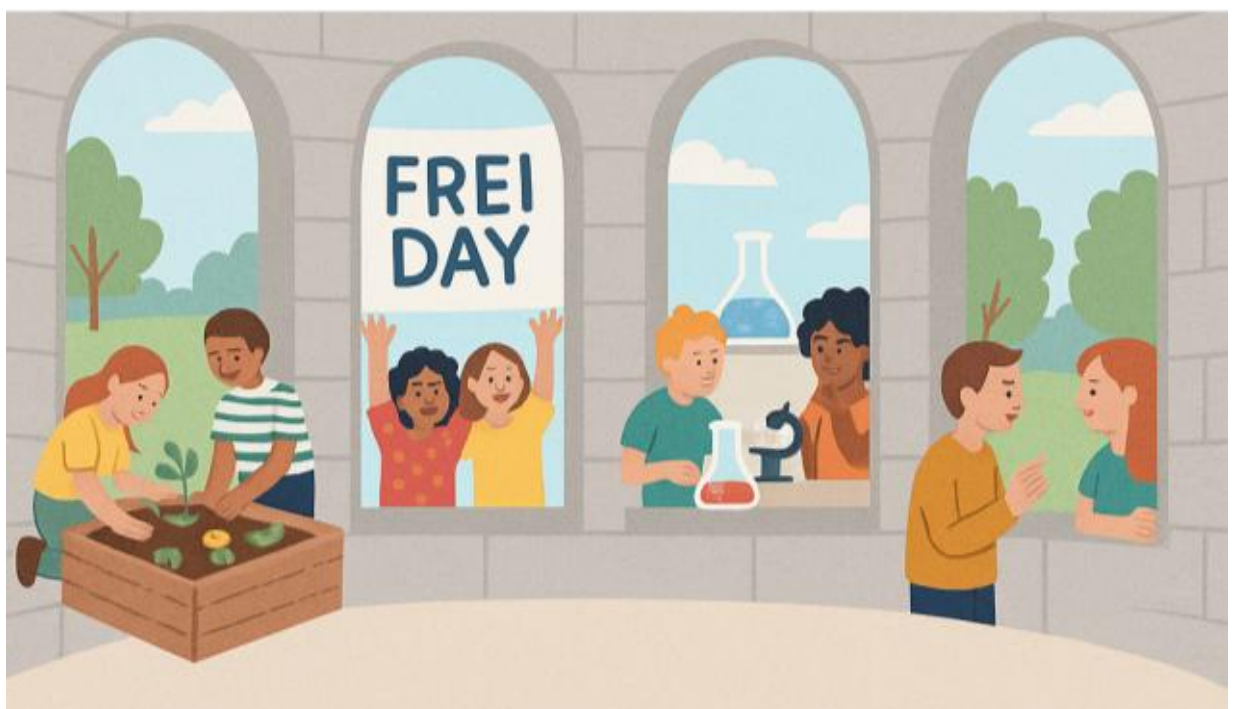

Wir alle schauen auf den FREI DAY mit unterschiedlichen Perspektiven, wie durch unterschiedliche Fenster. Führt zu verschiedenen Sichtweisen. Gesammelte Geschichten bieten verschiedene Sichtweisen darauf, welche wichtigen Veränderungen durch FREI DAY passieren. Uns interessiert, welche Perspektive ihr besonders wichtig für **Weiterentwicklung des FREI DAY** findet! Was ihr wichtig findet, hängt von eurer Rolle und persönlichem

Hintergrund ab, davon durch welches Fenster ihr blickt. Ziel ist, dass **andere von euch lernen** können, was euch besonders wichtig ist und warum. Deshalb gerne persönlich - was müssen andere wissen, um euch gut zu verstehen? Warum findet ihr welche Veränderung besonders wichtig?

Ablaufplan auf Flipchart 2 zeigen; Dauer ca. 2,5 Stunden

[1] Flipchart 3 Gesprächsrahmen:

- Beachten:
    - Wichtig, dass keine negativen Konsequenzen für die Anwesenden oder andere Schulmitglieder durch Gesagtes entstehen
    - Nicht versuchen, Anonymität der Geschichten aufzuheben. Geht um Inhalte des Gesagten
    - Wenn ihr nach dem Gespräch ein Anliegen habt, kommt gerne auf mich zu. Wenn ihr etwas braucht um gut sprechen zu können und euch sicher zu fühlen in der Gruppe, gern mitteilen
- Gesprächsrahmen:
    - Alles darf gesagt werden, kritisches und freudiges. Wir üben dabei: immer miteinander teilen, warum wir etwas wie empfinden. Anderen zuhören und aufeinander eingehen. Auf Redeanteile achten, diejenigen die weniger sprechen mal vorlassen, wenn man selbst viel spricht mal zurückhalten -> exploratorischer Dialog
    - Meldezeichen für gemeinsame Verantwortung: Aufnahmegerät rumgeben (A); Technischer Punkt: Pause, Bedürfnisse (T); Meldereihenfolge selbst mitzählen und anzeigen beim melden (Finger 1-5)
    - Gespräche in Großgruppe werden aufgezeichnet, in Kleingruppe nicht. Beim Sprechen Aufnahmegerät in die Hand nehmen, weitergeben an nächste Person die sprechen will (quasi wie Redestab)
    - Ideen und kleine Off-Topics, die euch wichtig sind, aber gerade nicht ganz in den Ablauf passen, könnt ihr jederzeit auf Modi-Karten schreiben und selbstständig in **Ideenhafen** hängen, damit später ein Platz dafür gesucht werden kann

Aufstellung im Raum, dabei miteinander reden. 5 mins: Wie viele Zimmerpflanzen; Wie wach bis müde; Wie sehr hast du Lust, die Schule mitzugestalten? 3.Frage, die Hierarchie senkt?
