# Supplementary material for: Study protocol for a quasi-experimental evaluation of the whole school approach FREI DAY
Source: Arch Public Health. 2026 Oct 13;84:222. doi: 10.1186/s13690-026-02088-7 (PMC13670517; doi:10.1186/s13690-026-02088-7)
Supplement: Supplementary file 4 [file 13690_2026_2088_MOESM13_ESM.pdf]

Supplement 13: Worksheet for the *Most Significant Change* story

Erzähle deine Geschichte der Veränderung!

Erzähle eine Veränderung durch den FREI DAY, die du besonders wichtig/ bedeutsam findest. Die Veränderung darf positiv oder negativ sein – sie muss aber tatsächlich geschehen sein!

Die folgenden Leitfragen helfen dir Schritt für Schritt, deine Geschichte zu erzählen.

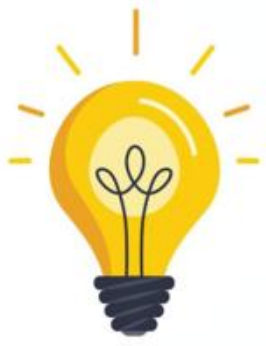

Tipp: Mach deine Geschichte lebendig, indem du beschreibst, was du gesehen, gehört, gefühlt, oder von anderen wahrgenommen hast. Welche Gedanken und Gefühle kamen bei dir auf?

Denke jetzt an deine Zeit im FREI DAY.

Sammle zunächst Stichpunkte in der Wolke:
Welche (positiven und negativen) Veränderungen sind durch den FREI DAY passiert?

Positiv +

Negativ -

Kreise die Veränderung aus der Wolke ein, die du besonders wichtig/ bedeutsam findest.
Beschreibe diese Veränderung in deiner Geschichte auf den nächsten Seiten.

Die folgenden Fragen helfen dir, **deine Geschichte** mit Anfang, Mitte und Schlussteil zu schreiben. Nutze **nur die Fragen**, die du **hilfreich** findest. **Erzähle in ganzen Sätzen.**

1) Anfang – Beschreibe eine Situation, in der die Veränderung durch den FREI DAY noch <u>nicht</u> passiert ist. Wie hast du dich vor der Veränderung gefühlt? *(z.B. unzufrieden, frustriert, verärgert, lustlos, gelangweilt, motivierter, entspannter, ...).* Beschreibe, warum es dir so ging.

____

____

____

____

____

____

2) Mitte – Beschreibe konkrete Momente, in denen du bemerkt hast, dass eine Veränderung entsteht. Wie wurde die Veränderung angestoßen? Beschreibe auch, was du bei der Veränderung gefühlt hast und warum *(z.B. Freude, Frust, Ärger, Motivation, Langeweile, ...).*

____

____

____

____

____

____

____

____

____

3) Schluss – Situation danach: Was ist jetzt anders? Wie bemerkst du, dass eine Veränderung passiert ist? Beschreibe einen Moment, in dem du gemerkt hast, dass sich etwas in dir oder um dich herum durch den FREI DAY verändert hat (in der Schule, Zuhause, im Verein, bei Freunden/Familie ...).

____

____

____

____

____

____

____

4) Warum ist diese Veränderung besonders bedeutsam/wichtig für dich? Du kannst auch zusätzlich einen Ausblick geben, was sich dadurch in Zukunft noch weiter verändern könnte.

____
____
____
____
____
____

Optional: Wie ist diese Veränderung möglich geworden? Beschreibe das nur, falls es in deiner Geschichte noch nicht vorkommt.

____
____
____
____

Gib deiner Geschichte eine **Überschrift**, die gut zur beschriebenen Veränderung passt. 2-3 Stichpunkte, die in deiner Geschichte vorkommen, reichen auch aus:

____

Weitere Informationen:

Schule: ________________

Rolle (passende ankreuzen):
- ☐ Schüler:in
- ☐ Eltern/Erziehungsberechtigte
- ☐ Lehrkraft
- ☐ FREI-DAY Projektbegleitung

Stell dir einen geheimen Code zusammen. Den Code brauchst du, falls du z.B. später nicht mehr möchtest, dass wir deine Geschichte weiterverwenden.

_____ Erster Buchstabe vom Vornamen deiner Mutter (z. B. Maria = M).
_____ Tag, an dem du Geburtstag hast (z. B. Geburtstag am 7. März = 7).
_____ Erster Buchstabe der Straße, in der du wohnst (z. B. Baumweg = B).
Falls du etwas nicht weißt, kannst du stattdessen ein **X** nehmen.

Möchtest du uns noch etwas mitteilen?

____
____

Letzte Seite nicht vergessen! :)

Vielen Dank, dass du deine Geschichte erzählt hast!

Fast geschafft! Bitte lies hier noch aufmerksam und kreuze an:

Mit der Abgabe deiner Geschichte stimmst du zu, dass sie für die Forschung verwendet werden darf (entsprechend der Beschreibung auf dem Informationsblatt). Deine Geschichte wird nicht mit deinem Namen verknüpft. Wenn du deine Geschichte ganz für dich behalten möchtest, gib sie nicht ab – dir entstehen keine Nachteile daraus. Vor der Abgabe kannst du ein Foto von deiner Geschichte machen, um eine Version für dich zu behalten.

Wenn du später merkst, dass deine Geschichte nicht oder anders weiterverwendet werden soll, tue folgendes:
1) Teile uns deinen geheimen Code mit (siehe vorherige Seite) und
2) teile uns mit, wie wir mit deiner Geschichte umgehen sollen (z.B. löschen, nur für die Forschung verwenden, usw.). Das hat keine Nachteile für dich und du musst keinen Grund angeben. Melde dich hierfür bei dem Kontakt, der auf dem Informationsblatt angegeben ist.

Wenn du hiermit einverstanden bist, **kreuze an:**

- ○ Ich stimme zu, dass meine Geschichte ohne meinen Namen für die Öffentlichkeitsarbeit der Schule auch nach außen hin verwendet werden darf (z.B. im Flur aushängen, auf die Website aufnehmen).
- ○ Ich stimme zu, dass meine Geschichte ohne meinen Namen für die Öffentlichkeitsarbeit zum FREI DAY über die Schule hinaus verbreitet werden darf.
