# Supplementary material for: Study protocol for a quasi-experimental evaluation of the whole school approach FREI DAY
Source: Arch Public Health. 2026 Oct 13;84:222. doi: 10.1186/s13690-026-02088-7 (PMC13670517; doi:10.1186/s13690-026-02088-7)
Supplement: Supplementary file 5 [file 13690_2026_2088_MOESM1_ESM.pdf]

Supplement 1: Systematic description of the complexity of FREI DAY – ten dimensions of complexity[1]

1. **Active components included in the intervention, in relation to the comparison:**

The FREI DAY consists of seven intervention components: (1) a curriculum anchored learning format (2) of at least four hours per week, (3) where students work in small groups (4) to come up with a project that addresses at least one of the Sustainable Development Goals (SDGs) (5) and then carry it out themselves (6) over the course of at least one school year and (7) supported by one or more internal or external collaboration partners. All of these components must be delivered as a bundle.

2. **Behaviour or actions of intervention recipients or participants to which the intervention is directed:**

The intervention addresses both teaching staff and students and targets a variety of different behaviour and actions. Teachers become learning facilitators and take on advisory, structuring and reflective roles, designing learning processes in a competence-oriented manner and supporting collaborative work. Students plan and structure their projects independently, work creatively and cooperatively, reflect on social issues, demonstrate empathetic and discursive behaviour, and engage with environmental and sustainability topics.

3. **Organisational levels and categories targeted by the intervention:**

Multi-level: The intervention targets three levels: individual (students, teachers), classes and school.

4. **The degree of tailoring intended or flexibility permitted across sites or individuals in applying or implementing the intervention:**

A certain degree of variation in the implementation of the FREI DAY intervention from site to site is permitted: the focus must be on the SDGs, but the selection of individual SDGs may vary. The selection of project topics is always up to the students, but the learning guides can provide guidance. FREI DAY

[1] Lewin S, Hendry M, Chandler J, Oxman AD, Michie S, Shepperd S, et al. Assessing the complexity of interventions within systematic reviews: development, content and use of a new tool (iCAT_SR). BMC Med Res Methodol. 2017;17:76. doi:10.1186/s12874-017-0349-x.

must be structurally anchored in the curriculum, but schools can decide for themselves on which dates FREI DAY will be implemented and which grades and classes will participate. In principle, FREI DAY should be held for four hours per week, but in some cases it may be shorter. The building of a network is one of the components of FREI DAY, but its implementation and form vary greatly. Knowledge and action must be linked, but this is implemented to varying degrees in the student projects.

5. **The level of skill required by those delivering the intervention in order to meet the intervention objectives:**

No specialised skills required.

6. **The level of skill required for the targeted behaviour when entering the included studies by those receiving the intervention, in order to meet the intervention objectives:**

No specialised skills required.

7. **The degree of interaction between intervention components, including the independence/interdependence of intervention components:**

It is to be expected that there will be substantial interaction or inter-dependency between intervention components or actions i.e., the delivery of one intervention component impacts on the delivery of another, resulting in a synergistic effect. The focus on the SDGs interacts strongly with the choice of topics for the student projects. The number of hours allocated to FREI DAY implementation interacts moderately with the linking of knowledge and action. The number of hours allocated to FREI DAY implementation interacts weakly with network building. The structural anchoring in the curriculum interacts moderately with cross-year implementation.

8. **The degree to which the effects of the intervention are dependent on the context or setting in which it is implemented:**

Moderately context dependent: It is to be expected that the effects of the intervention are likely to be transferrable across a limited range of settings only. The impact of the intervention depends on the

following contextual factors: degree of urbanisation, deprivation index, type of school, openness to innovation among school staff and parents.

9. The degree to which the effects of the intervention are changed by recipient or provider factors:

It is to be expected that the effects of the intervention will be modified by both recipient (age and language barriers among students) and provider factors (number of years of experience with FREI DAY, resources).

10. The nature of the causal pathway between the intervention and the outcome it is intended to effect:

Pathway variable, long: The causal pathway includes three or more steps between intervention and outcome and occurs over a long time period; is variable and more than one causal pathway has been proposed (see Figure 1 in the manuscript).
