# Supplementary material for: Study protocol for a quasi-experimental evaluation of the whole school approach FREI DAY
Source: Arch Public Health. 2026 Oct 13;84:222. doi: 10.1186/s13690-026-02088-7 (PMC13670517; doi:10.1186/s13690-026-02088-7)
Supplement: Supplementary file 6 [file 13690_2026_2088_MOESM2_ESM.pdf]

Supplement 2: Student questionnaire for primary schools

Schüler:innen-Befragung

Information für Lehrkräfte:
Bitte gehen Sie die ersten beiden Seiten dieses Dokuments mit den Kindern gemeinsam durch bzw. lesen Sie diese vor.

Mach mit bei unserer Umfrage!

Deine Schule macht 2025 zusammen mit der Medizinischen Hochschule Hannover eine Umfrage. Das ist eine Befragung, bei der wir wissen möchten, was Schulen tun können, damit es allen Schülerinnen, Schülern und ihren Familien gut geht.

Worum geht es?
Wir möchten wissen, wie du lebst, wie es dir geht, wie du mit Problemen und Sorgen umgehst, was dir wichtig ist und welche Meinung du zu bestimmten Themen hast, z.B. Umweltschutz.

Muss du mitmachen?
Nein, du entscheidest selbst! Wenn du nicht mitmachen möchtest oder eine Frage nicht beantworten willst, ist das völlig in Ordnung. Es passiert nichts Schlimmes, wenn du nicht teilnimmst.

Sind deine Antworten geheim?
Ja! Niemand erfährt, was du geantwortet hast – auch nicht deine Lehrer oder deine Eltern. Du musst auch nicht deinen Namen aufschreiben.

Möchtest du an der Umfrage teilnehmen?

☐ Ja

☐ Nein

Lies dir bitte erst die Anleitung durch. Dann kannst du mit dem Ausfüllen anfangen.

Vielen Dank!

Wie füllst du den Fragebogen aus?

Es gibt keine richtigen oder falschen Antworten. Deine ehrliche Meinung zu unseren Fragen ist uns sehr wichtig.

- Falls keine Antwort genau passt, wähle die, die am besten zu dir passt.
- Die meisten Fragen haben vier Antwortmöglichkeiten. Hier siehst du ein Beispiel:

	nein	eher nein	eher ja	ja
	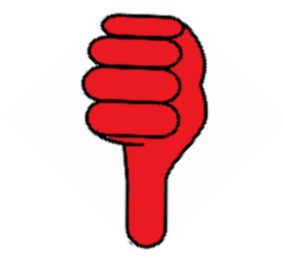	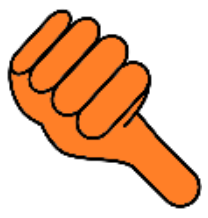	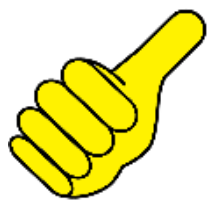	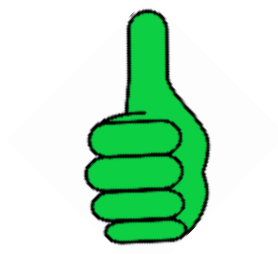
Ich esse gern Pizza	○	○	○	○

nein

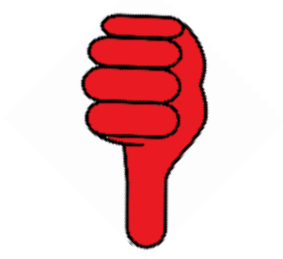

→ Das stimmt gar nicht für dich.

eher nein

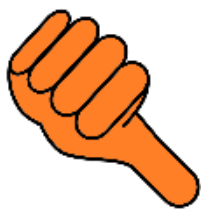

→ Das stimmt meistens nicht für dich.

eher ja

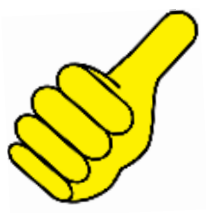

→ Das stimmt meistens für dich.

Ja

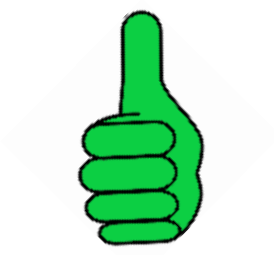

→ Das stimmt genau für dich.

- Wenn du auf dieses Symbol 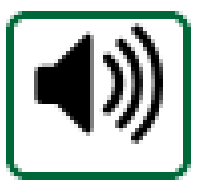 klickst, dann werden dir die Fragen vorgelesen.

Viel Spaß beim Mitmachen!

Abschnitt A: Einstiegsfragen

A1. Erstelle deinen geheimen Code

Wir möchten wissen, ob du später noch genauso denkst wie heute. Damit wir das herausfinden können, ohne deinen Namen zu kennen, bitten wir dich, einen Code mit drei Zahlen zu erfinden:

_____ Erster Buchstabe **vom Namen deiner Mama** (z. B. Maria = M).
_____ Tag, an dem du Geburtstag hast (z. B. Geburtstag am 7. Juli = 7).
_____ Erster Buchstabe deines Geburtsmonats (z. B. Juli = J).

Beispiel:

Wenn deine Mama Maria heißt, du am 6. Geburtstag hast und im Juli geboren bist, wäre dein Code: **M06J**.

Falls du etwas nicht weißt, kannst du stattdessen ein **X** nehmen.
Zum Beispiel: Wenn du deinen Geburtsmonat nicht kennst, könnte dein Code **M06X** sein.

Abschnitt B: Informationen zur Person

B1. Klassenstufe

In welche Klasse gehst du?

☐ 3. Klasse

☐ 4. Klasse

B2. Geschlecht

Bist du?

☐ Junge

☐ Mädchen

☐ weder Junge noch Mädchen

B3. Wohnsituation und Familienkonstellation

Mit wem lebst du Zuhause zusammen? (Mach bitte bei allem was zutrifft ein Kreuz!)

☐ Vater

☐ Mutter

☐ Stiefvater

☐ Stiefmutter

☐ Geschwister (auch Stief- und Halbgeschwister)

☐ andere Personen

B4. Migrationshintergrund

(A) Wurdest du in Deutschland geboren?

☐ ja

☐ nein

☐ weiß ich nicht

(B) Wurden deine leiblichen Eltern in Deutschland geboren`

Mutter	☐ ja	☐ nein	☐ weiß ich nicht
Vater	☐ ja	☐ nein	☐ weiß ich nicht

(C) Welche Sprache sprichst du Zuhause die meiste Zeit? (Bitte kreuze nur ein Feld an!)

☐ Deutsch

☐ eine andere Sprache

B5. Lebenssituation

(A) Hast du ein eigenes Zimmer nur für dich allein?

☐ ja

☐ nein

(B) Wie viele Bücher gibt es in deinem Zuhause?

☐ keine oder nur sehr wenige

☐ ein Regal

☐ mehr als ein Regal

(C) Gibt es bei dir Zuhause Musikinstrumente (z.B. ein Klavier, eine Gitarre, eine Blockflöte oder andere Instrumente)?

☐ ja

☐ nein

(D) Wie häufig bist du mit deiner Familie im letzten Jahr in den Urlaub ins Ausland gefahren?

☐ überhaupt nicht

☐ 1-mal

☐ 2-mal

☐ mehr als 2-mal

Abschnitt C: Jeder Mensch ist einzigartig

C0. Teilnahme FREI DAY [Frage nur für Interventionsschulen]

Deine Schule hat in einigen Klassenstufen den FREI DAY eingeführt. Beim FREI DAY haben Kinder jede Woche Zeit, sich mit eigenen Projekten für eine bessere Zukunft zu beschäftigen. Hast du schon mal am FREI DAY teilgenommen?

☐ ja → Filter: Frage C0.1 anzeigen

☐ nein → Filter: Frage C0.1 überspringen und Frage C1 anzeigen

C0.1 FREI DAY Wissenstransfer [Frage nur für Interventionsschulen]

Stimmt das für dich?:

Was ich beim FREI DAY gelernt habe, kann ich auch im normalen Unterricht nutzen. ☐ ja

☐ nein

☐ eher nein

☐ eher ja

☐ ja

☐ weiß ich nicht

C1. Schulinvolvement/ Wohlbefinden

Stimmt das für dich?

	nein	eher nein	eher ja	ja	weiß ich nicht
(A) Ich gehe ziemlich gerne in die Schule.					
(B) Ich fühle mich im Unterricht wohl.					
(C) Ich lerne gerne in der Schule.					

C2. Erlernen von Kooperations- und Diskussionskompetenz

Stimmt das für dich?

	nein	eher nein	eher ja	ja	weiß ich nicht
(A) Wir lernen, worauf es beim Diskutieren ankommt.					
(B) Wir lernen, wie man in Gruppen gut zusammenarbeiten kann.					
(C) Wir lernen, wie man andern helfen kann, um das Gelernte besser zu verstehen.					
(D) Wir lernen, wie man in der Diskussion seine Meinung begründet.					

C3. Selbstwirksamkeitserwartung und Sozialkompetenz

Stimmt das für dich?

	nein	eher nein	eher ja	ja	weiß ich nicht
(A) Wenn ich aufgeregt bin, kann ich mich beruhigen.					
(B) Wenn ich mir etwas wünsche und es nicht sofort bekomme, kann ich warten.					
(C) Wenn ich traurig bin, weiß ich was ich tun muss, damit es mir besser geht.					

(Fortsetzung Frage C3)	nein	eher nein	eher ja	ja	weiß ich nicht
(D) Wenn ich wütend bin kann ich trotzdem mit anderen sprechen.					

C4. Perspektivübernahme

Stimmt das für dich?

	nein	eher nein	eher ja	ja	weiß ich nicht
(A) Bei Meinungsverschiedenheiten versuche ich, die Sache aus Sicht aller Beteiligten zu betrachten.					
(B) Wenn ich versuche meine Freunde besser zu verstehen, stelle ich mir vor, wie die Dinge für sie aussehen.					
(C) Bevor ich Personen kritisiere, versuche ich mir vorzustellen, wie es mir an ihrer Stelle ginge.					

C5. Empathie

Stimmt das für dich?

	nein	eher nein	eher ja	ja	weiß ich nicht
(A) Die Gefühle von Personen, die ich gerne mag, bedeuten mir viel.					
(B) Ich erkenne im Gesichtsausdruck einer Person, ob sie gerade glücklich, überrascht, traurig oder ängstlich ist.					
(C) Wenn jemand eine schlechte Schulnote bekommt, kann ich mir gut vorstellen, wie sich diese Person fühlt.					
(D) Wenn jemand ganz andere Wünsche hat als ich, kann ich das gut nachempfinden.					

C6. Kreatives Denken

Stimmt das für dich?

	nein	eher nein	eher ja	ja	weiß ich nicht
(A) Für Probleme finde ich häufig Lösungen, an die andere noch nicht gedacht haben.					
(B) Wenn ich eine Idee höre, traue ich mir zu, sie zu verbessern.					

(Fortsetzung Frage C6)	nein	eher nein	eher ja	ja	weiß ich nicht
(C) Andere sagten mir schon häufiger, ich hätte einfallsreiche Ideen.					

C7. Problemlösefähigkeit

Stimmt das für dich?

	nein	eher nein	eher ja	ja	weiß ich nicht
(A) Wenn ich ein Problem habe, erkenne ich es an.					
(B) Wenn ich ein Problem habe, weiß ich, wie es dazu kam.					
(C) Wenn ich ein Problem nicht allein lösen kann, suche ich mir Hilfe.					
(D) Bei Problemen überlege ich auch, ob ich selbst etwas dazu beitrage.					

C8. Allgemeine Selbstwirksamkeitserwartung

Stimmt das für dich?

	nein	eher nein	eher ja	ja	weiß ich nicht
(A) Wenn ich mich richtig anstrenge, dann klappt das was ich will.					
(B) Ich kann sehr viele Dinge.					
(C) Ich kann mich richtig freuen, wenn ich etwas geschafft habe.					
(D) Wenn mir etwas gelingt, dann deshalb, weil ich es gut kann.					

C9. Optimismus

Stimmt das für dich?

	nein	eher nein	eher ja	ja	weiß ich nicht
(A) Ich lache oft.					
(B) Ich finde mich toll.					
(C) Ich bin oft länger traurig.					
(D) Manchmal weiß ich gar nicht wie es mir geht. (Ob ich traurig, ängstlich, wütend oder fröhlich bin)					

C10. Zukunftsangst

Wie viel Angst hast du davor, dass sich diese Dinge in Zukunft verschlechtern?

	sehr viel Angst	eher viel Angst	eher wenig Angst	keine Angst	weiß ich nicht
(A) Umwelt und Wetter werden schlechter.					
(B) Unterricht in Schulen wird schlechter.					
(C) Menschen sind ungerechter zueinander.					
(D) Kriege oder Angriffe werden mehr.					
(E) Kranke Menschen bekommen weniger Hilfe.					
(F) Verbrechen und Gewalt werden mehr.					
(G) Menschen haben nicht genug Geld zum Leben.					
(H) Mehr Menschen finden keine Arbeit.					

Abschnitt D: Umwelt und Nachhaltigkeit

D1. Umweltbewusstsein und Verantwortungszuschreibung für Umweltprobleme

Stimmt das für dich?

	nein	eher nein	eher ja	ja	weiß ich nicht
(A) Wenn es so weitergeht wie bisher, gibt es schreckliche Umweltzerstörungen.					
(B) Menschliche Erfindungen werden dafür sorgen, dass die Erde bewohnbar bleibt.					
(C) Pflanzen und Tiere haben die gleichen Rechte zu existieren wie Menschen.					
(D) Die Natur ist stark genug, um die Auswirkungen der Umweltverschmutzung auszugleichen.					
(E) Der Klimawandel wird oft übertrieben dargestellt.					

D2. Bewusstsein für Umweltkonsequenzen

Stimmt das für dich?

	nein	eher nein	eher ja	ja	weiß ich nicht
(A) Der Klimawandel ist ein Problem für uns alle.					
(B) Der Verbrauch von Erdöl und Kohle ist problematisch.					
(C) Der Natur geht es besser, wenn wir weniger Energie verbrauchen.					
(D) Es ist nicht sicher, ob der Klimawandel ein echtes Problem ist.					

D3. Verantwortungszuschreibung für Umweltprobleme

Stimmt das für dich?

	nein	eher nein	eher ja	ja	weiß ich nicht
(A) Ich bin mitverantwortlich für Probleme auf der ganzen Welt.					
(B) Ich bin mitverantwortlich für den Verbrauch von Rohstoffen der Erde.					
(C) Ich bin mitverantwortlich für die Erwärmung der Erde.					
(D) Einzelne können Probleme auf der ganzen Welt nicht verringern.					

Abschnitt E: Kontrollfragen

E1. Kontrollfragen zur ehrlichen Beantwortung

Stimmt das für dich?

	nein	eher nein	eher ja	ja	weiß ich nicht
(A) Ich habe alle Fragen sorgfältig beantwortet.					
(B) Ich habe alle Fragen ehrlich beantwortet.					

Abschluss

Das ist das Ende der Umfrage.

Vielen Dank für deine Teilnahme!

Wir möchten uns ganz herzlich für deine Mithilfe bedanken.
Du kannst das Browser-Fenster nun schließen.
