# Supplementary material for: Study protocol for a quasi-experimental evaluation of the whole school approach FREI DAY
Source: Arch Public Health. 2026 Oct 13;84:222. doi: 10.1186/s13690-026-02088-7 (PMC13670517; doi:10.1186/s13690-026-02088-7)
Supplement: Supplementary file 7 [file 13690_2026_2088_MOESM3_ESM.pdf]

Supplement 3: Student questionnaire for secondary schools

Schüler:innen-Befragung

Mach mit bei unserer Umfrage!

Deine Schule führt 2025 zusammen mit uns, der Medizinischen Hochschule Hannover, eine Umfrage durch. Wir möchten herausfinden, was Schulen tun können, damit es allen Schülerinnen, Schülern und ihren Familien gut geht

Worum geht es in dem Fragebogen?
Wir fragen dich unter anderem, wie du lebst, wie es dir geht, wie du mit Problemen und Sorgen umgehst, was dir wichtig ist und welche Meinung du zu bestimmten Themen hast, z.B. Umweltschutz.

Muss ich daran teilnehmen?
Die Teilnahme ist freiwillig. Es entstehen dir keine Nachteile, wenn du nicht mitmachst.

Was passiert mit meinen Antworten?
Deine Antworten sind **geheim**. Du wirst nicht nach deinem Namen gefragt, und niemand an deiner Schule oder in deiner Familie wird erfahren, was du geantwortet hast.

Möchtest du an der Umfrage teilnehmen?

☐ Ja, ich möchte an der Umfrage teilnehmen.

☐ Nein ich möchte nicht an der Umfrage teilnehmen.

Lies dir bitte erst die Anleitung durch. Dann kannst du mit dem Ausfüllen anfangen.

Vielen Dank!

Wie fülle ich den Fragebogen aus?

Es gibt keine richtigen oder falschen Antworten. Deine ehrliche Meinung zu unseren Fragen ist uns sehr wichtig.

- Wenn mal keine der Antwortmöglichkeiten so richtig passt, dann nimm die Antwort, die deiner eigenen Meinung am nächsten kommt.
- Wenn eine Frage auf dich nicht zutrifft, du die Frage nicht verstehst oder die Antwort nicht weißt, dann kreuze bitte „weiß ich nicht" an.
- Wenn du zu einer Frage etwas mitteilen willst, dann hast du am Ende des Fragebogens die Gelegenheit dazu.

Viel Spaß beim Mitmachen!

Abschnitt A: Einstiegsfragen

A1. Erstelle deinen geheimen Code

Wir möchten wissen, ob du später noch genauso denkst wie heute. Damit wir das herausfinden können, ohne deinen Namen zu kennen, bitten wir dich, einen Code mit drei Zahlen zu erfinden:

______ Erster Buchstabe des Vornamens deiner Mutter (z.B. Maria = M).

______ Tag deines Geburtstags (z. B. Geburtstag am 06. Juli = 06)

______ Erster Buchstabe deines Geburtsmonats (z.B. Juli = J).

Beispiel:
Wenn der Vorname deiner Mutter **M**aria heißt, du am **06.** Geburtstag hast und im **J**uli geboren bist, wäre dein Code: **M06J**.

Falls du eine Angabe nicht weißt, kannst du stattdessen ein **X** verwenden.

Zum Beispiel: Wenn du deinen Geburtsmonat nicht weißt, könnte dein Code **M06X** sein

Abschnitt B: Informationen zur Person

B1. Klassenstufe

In welche Klasse gehst du? [Einfachauswahl von Klasse 5 -13 und „Ich besuche eine berufsbildende Schule“]

☐ Klasse 5-7
→ Filter: bei Frage A3 Antwortoption 1 anzeigen

☐ Klasse 8-13 oder „Ich besuche eine beruflich bildende Schule“
→ Filter. bei Frage A3 Antwortoption 2 anzeigen

B2. Geschlecht

Bist du?

[Antwortoption 1]

☐ Junge

☐ Mädchen

☐ weder Junge noch Mädchen

[Antwortoption 2]

☐ männlich

☐ weiblich

☐ nicht-binär

B3. Wohnsituation und Familienkonstellation

Mit wem lebst du Zuhause zusammen? (Mach bitte bei allem was zutrifft ein Kreuz!)

☐ Vater

☐ Mutter

☐ Stiefvater

☐ Stiefmutter

☐ Geschwister (auch Stief- und Halbgeschwister)

☐ andere Personen

B4. Migrationshintergrund

(A) Wurdest du in Deutschland geboren?

☐ ja

☐ nein

☐ weiß ich nicht

(B) Wurden deine leiblichen Eltern in Deutschland geboren?

Mutter	☐ ja	☐ nein	☐ weiß ich nicht
Vater	☐ ja	☐ nein	☐ weiß ich nicht

(C) Welche Sprache sprichst du Zuhause die meiste Zeit? (Bitte kreuze nur ein Feld an!)

☐ Deutsch

☐ eine andere Sprache

B5. Lebenssituation

(A) Hast du ein eigenes Zimmer nur für dich allein?

☐ ja

☐ nein

(B) Wie viele Bücher gibt es in deinem Zuhause?

☐ keine oder nur sehr wenige

☐ ein Regal

☐ mehr als ein Regal

(C) Gibt es bei dir Zuhause Musikinstrumente (z.B. ein Klavier, eine Gitarre, eine Blockflöte oder andere Instrumente)?

☐ ja

☐ nein

(D) Wie häufig bist du mit deiner Familie im letzten Jahr in den Urlaub ins Ausland gefahren?

☐ überhaupt nicht

☐ 1-mal

☐ 2-mal

☐ mehr als 2-mal

Abschnitt C: Jeder Mensch ist einzigartig

C0. Teilnahme FREI DAY [Frage nur für Interventionsschulen]

Deine Schule hat in einigen Klassenstufen den FREI DAY eingeführt. Beim FREI DAY haben Schülerinnen und Schüler jede Woche Zeit, sich mit eigenen Projekten für eine bessere Zukunft zu beschäftigen. Hast du schon mal am FREI DAY teilgenommen?

☐ ja → Filter: Frage C0.1 anzeigen

☐ nein → Filter: Frage C0.1 überspringen und Frage C1 anzeigen

C0.1 FREI DAY Wissenstransfer [Frage nur für Interventionsschulen]

Inwieweit stimmst du dieser Aussage zu?:
Was ich beim FREI DAY gelernt habe, kann ich auch im normalen Unterricht nutzen.

☐ stimme nicht zu

☐ stimme eher nicht zu

☐ stimme eher zu

☐ stimme zu

☐ weiß ich nicht

C1. Schulinvolvement/ Wohlbefinden

Inwieweit stimmst du folgenden Aussagen über dich zu?

	stimme nicht zu	stimme eher nicht zu	stimme eher zu	stimme zu	weiß ich nicht
(A) Ich gehe ziemlich gerne in die Schule.					
(B) Ich fühle mich im Unterricht wohl.					
(C) Ich lerne gerne in der Schule.					

C2. Erlernen von Kooperations- und Diskussionskompetenz

Inwieweit stimmst du den folgenden Aussagen zu deiner Schule zu?: Wir lernen, …

	stimme nicht zu	stimme eher nicht zu	stimme eher zu	stimme zu	weiß ich nicht
(A) …worauf es beim Diskutieren ankommt.					
(B) …wie man in Gruppen gut zusammenarbeiten kann.					
(C) …wie man andern helfen kann, um das Gelernte besser zu verstehen.					
(D) …wie man in der Diskussion seine Meinung begründet.					

C3. Selbstwirksamkeitserwartung und Sozialkompetenz

Inwieweit stimmst du folgenden Aussagen über dich zu?

	stimme nicht zu	stimme eher nicht zu	stimme eher zu	stimme zu	weiß ich nicht
(A) Ich traue mich zu sagen, was ich denke, auch wenn die anderen nicht meiner Meinung sind.					
(B) Auch in einer ganz neuen Klasse kann ich schnell neue Freunde finden.					
(C) Wenn mich jemand ärgert, kann ich mich wehren, ohne Gewalt anzuwenden.					
(D) Wenn ich etwas Falsches getan habe, schaffe ich es, mich zu entschuldigen.					
(E) Wenn ich mich ganz traurig und mies fühle, schaffe ich es, mit anderen darüber zu sprechen.					
(F) Wenn mich jemand ärgert, schaffe ich es trotzdem, ruhig zu bleiben.					
(G) Auch wenn mir alles zu viel wird, schaffe ich es, meine schlechte Laune nicht an anderen auszulassen.					

C4. Perspektivübernahme

Inwieweit stimmst du folgenden Aussagen über dich zu?

	stimme nicht zu	stimme eher nicht zu	stimme eher zu	stimme zu	weiß ich nicht
(A) Bei Meinungsverschiedenheiten versuche ich, die Sache aus Sicht aller Beteiligten zu betrachten.					
(B) Ich versuche manchmal meine Freunde besser zu verstehen, indem ich mir vorstelle, wie die Dinge für sie aussehen.					
(C) Bevor ich Leute kritisiere, versuche ich mir vorzustellen, wie es mir an ihrer Stelle ginge.					

C5. Empathie

Inwieweit stimmst du folgenden Aussagen über dich zu?

	stimme nicht zu	stimme eher nicht zu	stimme eher zu	stimme zu	weiß ich nicht
(A) Die Gefühle mir nahestehender Personen sind für mich von Bedeutung.					

(Fortsetzung Frage C5)	stimme nicht zu	stimme eher nicht zu	stimme eher zu	stimme zu	weiß ich nicht
(B) Ich erkenne im Gesichtsausdruck einer Person, ob sie gerade glücklich, überrascht, traurig oder ängstlich ist.					
(C) Wenn jemand eine schlechte Schulnote bekommt, kann ich mir gut vorstellen, wie sich diese Person fühlt.					
(D) Wenn andere Personen ganz andere Bedürfnisse und Wünsche haben als ich, kann ich das gut nachvollziehen.					

C6. Kreatives Denken

Inwieweit stimmst du folgenden Aussagen über dich zu?

	stimme nicht zu	stimme eher nicht zu	stimme eher zu	stimme zu	weiß ich nicht
(A) Für Probleme finde ich häufig Lösungen, an die mein Umfeld noch nicht gedacht hat.					
(B) Wenn ich eine Idee höre, traue ich mir zu, sie zu verbessern/ weiterzuentwickeln.					
(C) Wenn ich eine Aufgabe nicht lösen kann, nehme ich einen anderen Blickwinkel ein, um doch zu einer Lösung zu kommen.					
(D) Ich versuche lieber etwas Neues als immer nur zu wiederholen, was ich oder mein Umfeld schon oft gemacht haben.					
(E) Andere sagten mir schon häufiger, ich hätte einfallsreiche Ideen.					

C7. Problemlösefähigkeit

Inwieweit stimmst du folgenden Aussagen über dich zu?

	stimme nicht zu	stimme eher nicht zu	stimme eher zu	stimme zu	weiß ich nicht
(A) Wenn ich ein Problem habe, erkenne ich es an.					
(B) Wenn ich ein Problem habe, weiß ich, wie es dazu kam.					
(C) Wenn ich ein Problem nicht allein lösen kann, suche ich mir Hilfe.					

(Fortsetzung Frage C7)	stimme nicht zu	stimme eher nicht zu	stimme eher zu	stimme zu	weiß ich nicht
(D) Bei Problemen überlege ich auch, ob ich selbst etwas dazu beitrage.					

C8. Metakognition (Planen und Strukturieren)

Inwieweit stimmst du folgenden Aussagen über dich zu?
Wenn ich eine etwas schwierigere Aufgabe löse ...

	stimme nicht zu	stimme eher nicht zu	stimme eher zu	stimme zu	weiß ich nicht
(A)...mache ich mir einen Arbeitsplan.					
(B)...schreibe ich wichtige Dinge heraus und ordne sie so an, dass ich sehe, was zusammengehört.					
(C)...plane ich zuerst, wie ich die Arbeit anpacken will.					

C9. Allgemeine Selbstwirksamkeitserwartung

Hier geht es um Deine persönlichen Einschätzungen und Gefühle. Bitte gib an, was am ehesten zutrifft!

	stimme nicht zu	stimme eher nicht zu	stimme eher zu	stimme zu	weiß ich nicht
(A) Wenn sich Herausforderungen auftun, finde ich Mittel und Wege, mich durchzusetzen.					
(B) Die Lösung schwieriger Probleme gelingt mir immer, wenn ich mich darum bemühe.					
(C) In unerwarteten Situationen weiß ich immer, wie ich mich verhalten soll.					
(D) Auch bei überraschenden Ereignissen glaube ich, dass ich gut mit ihnen zurechtkommen werde.					
(E) Schwierigkeiten sehe ich gelassen entgegen, weil ich meinen Fähigkeiten immer vertrauen kann.					
(F) Was auch immer passiert, ich werde schon klarkommen.					
(G) Für jedes Problem kann ich eine Lösung finden.					

C10. Optimismus

Hier geht es um Deine persönlichen Einschätzungen und Gefühle. Bitte gib an, was am ehesten zutrifft!

	stimme nicht zu	stimme eher nicht zu	stimme eher zu	stimme zu	weiß ich nicht
(A) Ich glaube, dass sich mein Leben positiv entwickeln wird.					
(B) Mein Leben macht Spaß.					
(C) Es kommt immer wieder etwas, worauf ich mich freuen kann.					
(D) Ich werde eines Tages im Berufsleben erfolgreich sein.					
(E) Ich glaube, dass ich in Zukunft mit meinen Freunden viel Spaß haben werde.					
(F) Was auch immer passiert, ich kann die gute Seite daran sehen.					

C11. Zukunftsangst

Manche Kinder und Jugendliche machen sich Sorgen, dass sich bestimmte Dinge in der Zukunft verschlechtern.
Wie viel Angst hast du davor, dass sich diese Dinge verschlechtern?

	sehr viel Angst	eher viel Angst	Eher wenig Angst	keine Angst	weiß ich nicht
(A) Umwelt und Klima					
(B) Schule und Bildungswesen					
(C) Soziale Gerechtigkeit					
(D) Kriege und Terrorismus					
(E) Medizinische Versorgung					
(F) Kriminalität					
(G) Wirtschaftliche Lage					
(H) Arbeitslosigkeit					

Abschnitt D: Umwelt und Nachhaltigkeit

D1. Umweltbewusstsein und Verantwortungszuschreibung für Umweltprobleme

Im Folgenden findest du Aussagen über die Beziehung zwischen Mensch und Umwelt. Inwieweit stimmst du diesen zu?

	stimme nicht zu	stimme eher nicht zu	stimme eher zu	stimme zu	weiß ich nicht
(A) Wenn es so weitergeht wie bisher, steuern wir auf eine Umweltkatastrophe zu.					
(B) Der menschliche Erfindungsgeist wird dafür sorgen, dass die Erde bewohnbar bleibt.					
(C) Pflanzen und Tiere haben die gleichen Rechte zu existieren wie Menschen.					
(D) Die Natur ist stark genug, um die Auswirkungen der Umweltverschmutzung auszugleichen.					
(E) Der Klimawandel wird oft übertrieben dargestellt.					
(F) Das Gleichgewicht der Natur ist sehr zerbrechlich.					

D2. Bewusstsein für Umweltkonsequenzen

Inwieweit stimmst du diesen zu?

	stimme nicht zu	stimme eher nicht zu	stimme eher zu	stimme zu	weiß ich nicht
(A) Der Klimawandel ist ein Problem für unsere Gesellschaft.					
(B) Energieeinsparung hilft dem Klimawandel entgegenzuwirken.					
(C) Die Erschöpfung von fossilen Brennstoffen ist problematisch.					
(D) Die Qualität der Umwelt wird sich verbessern, wenn wir weniger Energie verbrauchen.					
(E) Es ist nicht sicher, ob der Klimawandel ein echtes Problem ist.					

D3. Verantwortungszuschreibung für Umweltprobleme

Inwieweit stimmst du diesen zu?

	stimme nicht zu	stimme eher nicht zu	stimme eher zu	stimme zu	weiß ich nicht
(A) Ich bin mitverantwortlich für globale Probleme.					
(B) Ich bin mitverantwortlich für die Ausschöpfung der Ressourcen der Erde.					
(C) Ich bin mitverantwortlich für die Erderwärmung.					
(D) Mein Beitrag zu den globalen Problemen ist minimal.					
(E) Neben der Regierung und der Industrie bin auch ich für den hohen Ressourcenverbrauch verantwortlich.					
(F) Prinzipiell können Einzelne nichts zu der Reduzierung von globalen Problemen beitragen.					

Abschnitt E: Kontrollfragen

E1. Kontrollfragen zur ehrlichen Beantwortung

Inwieweit stimmst du folgenden Aussagen über dich zu?

	stimme nicht zu	stimme eher nicht zu	stimme eher zu	stimme zu	weiß ich nicht
(A) Ich habe alle Fragen gewissenhaft beantwortet.					
(B) Ich habe alle Fragen ehrlich beantwortet.					

Abschluss

Möchtest du uns noch etwas mitteilen? Nutze dafür das Kommentarfeld direkt unter diesem Text.

Vielen Dank für deine Teilnahme!

Wir möchten uns ganz herzlich für deine Mithilfe bedanken.
Du kannst das Browser-Fenster nun schließen.
