# Supplementary material for: Study protocol for a quasi-experimental evaluation of the whole school approach FREI DAY
Source: Arch Public Health. 2026 Oct 13;84:222. doi: 10.1186/s13690-026-02088-7 (PMC13670517; doi:10.1186/s13690-026-02088-7)
Supplement: Supplementary file 8 [file 13690_2026_2088_MOESM4_ESM.pdf]

Name: ______________________ Datum: ______________________

Elektropolis – eine Stadt der Zukunft in einem Kinderbuch von Erich Kästner

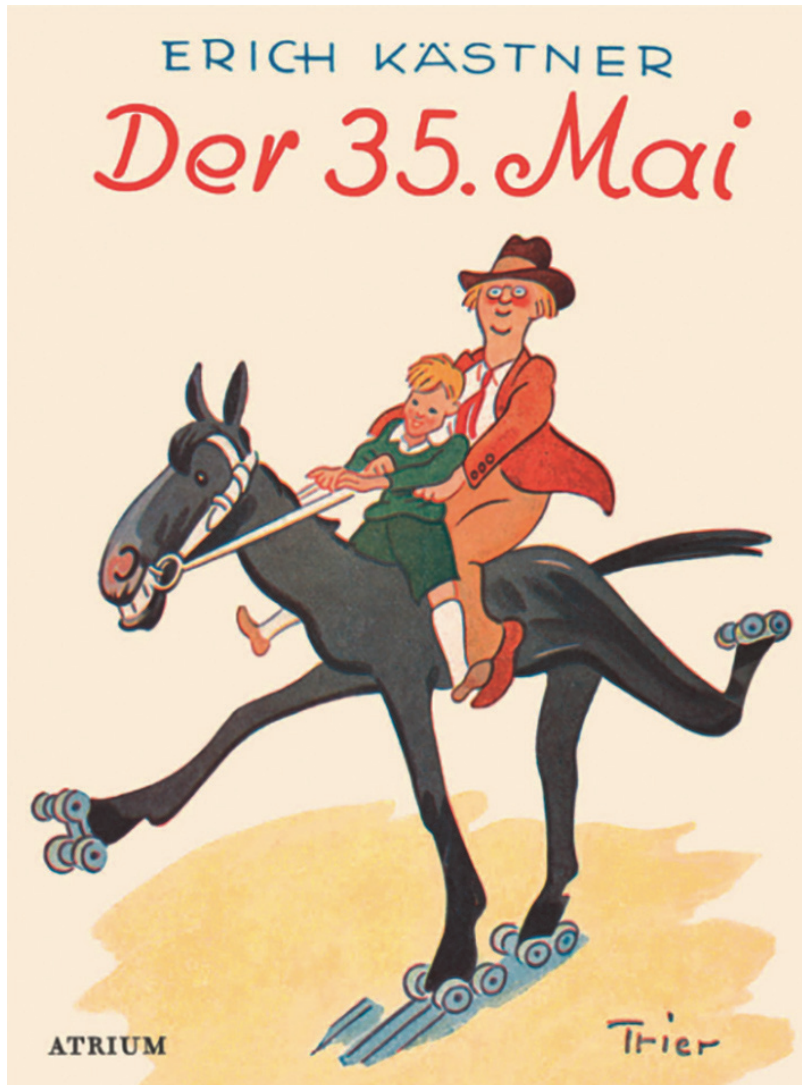

Im Jahr 1931 schrieb der bekannte Schriftsteller Erich Kästner ein Buch zu einer Reise in die Südsee. Der Titel „Der 35. Mai" verrät, dass es hier um eine etwas verrückte Fantasiegeschichte geht.

Drei Reisende – der Schüler Konrad, sein Onkel Ringelhuth und ein Pferd – erleben Abenteuer auf dem Weg in die Südsee. Auf dieser Reise gelangen sie zu der Stadt „Elektropolis". Dazu steigen sie in eine U-Bahn ein, die automatisch und ohne Fahrer fährt. 1931 gab es in Berlin schon eine U-Bahn und auch Autos, aber die wurden von Menschen gesteuert.

Hier ein Ausschnitt aus der Geschichte von 1931:

„Zu arbeiten scheint hier überhaupt niemand", meinte Ringelhuth. „Alles fährt im Auto spazieren. Versteht ihr das?"

Konrad, der neugierig neben einem Wagen hergerannt war, kam zurück und schüttelte den Kopf. „Denkt euch bloß", sagte er, „die Autos fahren von ganz allein, ohne Schofför *(=Fahrer)* und ohne Steuerung. Mir ist das völlig schleierhaft." Da bremste ein Wagen und hielt neben ihnen. Eine nette alte Dame saß hinten drin. Sie [...] fragte freundlich: „Sie sind wohl von auswärts?"

„Es reicht", erwiderte der Onkel. „Können Sie uns erklären, wieso hier die Autos von selber fahren?"

Die alte Dame lächelte. „Unsere Wagen werden ferngelenkt", erzählte sie. „Das Lenkverfahren beruht auf der sinnreichen Koppelung eines elektromagnetischen Felds mit einer Radiozentrale. Ganz einfach, was?" [...]

Sie bogen in eine belebte Straße ein, um sich die Schaufenster von Elektropolis zu betrachten. Aber kaum hatten sie die Bürgersteige betreten, so fielen sie alle drei der Länge lang um [...].

„Hilfe!" schrie Konrad. „Der Fußsteig ist lebendig!"

Der Fußsteig war nämlich, damit man nicht zu gehen brauchte, mit einem laufenden Band versehen. Darauf stellte man sich und fuhr [...] durch die Straßen. Wenn man in ein Geschäft wollte, trat man vom laufenden Band herunter [...].

Am meisten imponierte ihnen aber folgendes: Ein Herr, der vor ihnen auf dem Trottoir *(=Bürgersteig)* lang fuhr, trat plötzlich aufs Pflaster, zog einen Telefonhörer aus der Manteltasche, sprach eine Nummer hinein und rief: „Gertrud, hör mal, ich komme heute eine Stunde später zum Mittagessen." [...] Dann steckte er sein Taschentelefon wieder weg, trat auf das laufende Band, las in einem Buch und fuhr seiner Wege." (Kästner 1970/1931, S. 98 ff.)

Die drei Reisenden erleben noch andere Dinge in der Stadt Elektropolis, in der alles automatisch läuft und niemand arbeiten muss. Allerdings fängt die Stadt dann plötzlich an, sich selbst zu zerstören ...

Friedrich Verlag GmbH I Grundschule Sachunterricht 96 I 2022 I Zum Beitrag S. 08–14
Text: © Quelle: Erich Kästner: Der 35. Mai. Atrium Verlag 2018, S. 98 ff., ISBN 9783037921128, Cover: Atrium Verlag, Zürich 1935

Name: __________ Datum: __________

Elektropolis – eine Stadt der Zukunft in einem Kinderbuch von Erich Kästner (Teil 2)

Wenn du mehr wissen willst, kannst du das Buch „Der 35. Mai" lesen.

Lies den Text von Erich Kästner.

Sind die Ideen von 1931 in dieser Geschichte heute Wirklichkeit?
Was gibt es davon heute?

Wie findest du die Erfindungen in der Geschichte von Erich Kästner?

Sind die Erfindungen für alle Menschen gleich gut?

Welche Gefahren können von den Erfindungen ausgehen?

Wie würde deine Stadt der Zukunft aussehen?

Friedrich Verlag GmbH | Grundschule Sachunterricht 96 | 2022 | Zum Beitrag S. 08–14
