# Supplementary material for: Study protocol for a quasi-experimental evaluation of the whole school approach FREI DAY
Source: Arch Public Health. 2026 Oct 13;84:222. doi: 10.1186/s13690-026-02088-7 (PMC13670517; doi:10.1186/s13690-026-02088-7)
Supplement: Supplementary file 9 [file 13690_2026_2088_MOESM5_ESM.pdf]

UNESCO-Erbeformen in Deutschland

Aufgaben

1. Nenne die Anzahl der Welterbestätten in Deutschland und in Niedersachsen.
2. Verorte die Beispiele A-E für Erbeformen der UNESCO (M2) in der Karte (M1).
3. Nenne die Bundesländer, in denen (globale) Geoparks zu finden sind.
4. a) Überprüfe, ob in Niedersachsen Weltdokumentenerbe zu finden ist.
   b) Bestimme das Bundesland mit dem meisten Weltdokumentenerbe.
5. Bestimme die Anzahl der grenzüberschreitenden oder transnationalen Welterbstätten.
6. Plane eine fünftägige Reise in Deutschland, auf der du von allen Erbeformen mindestens eine vor Ort besichtigen kannst.

M2 Beispiele für UNESCO-Erbeformen

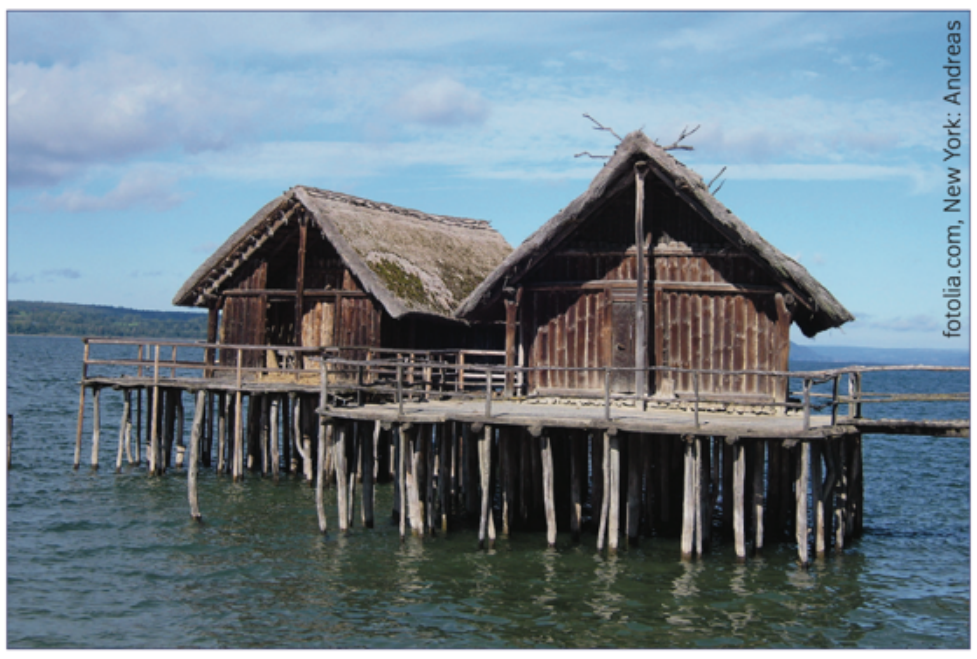

A Beispiel für Welterbe: Prähistorische Pfahlbauten um die Alpen (Foto: Rekonstruktion)

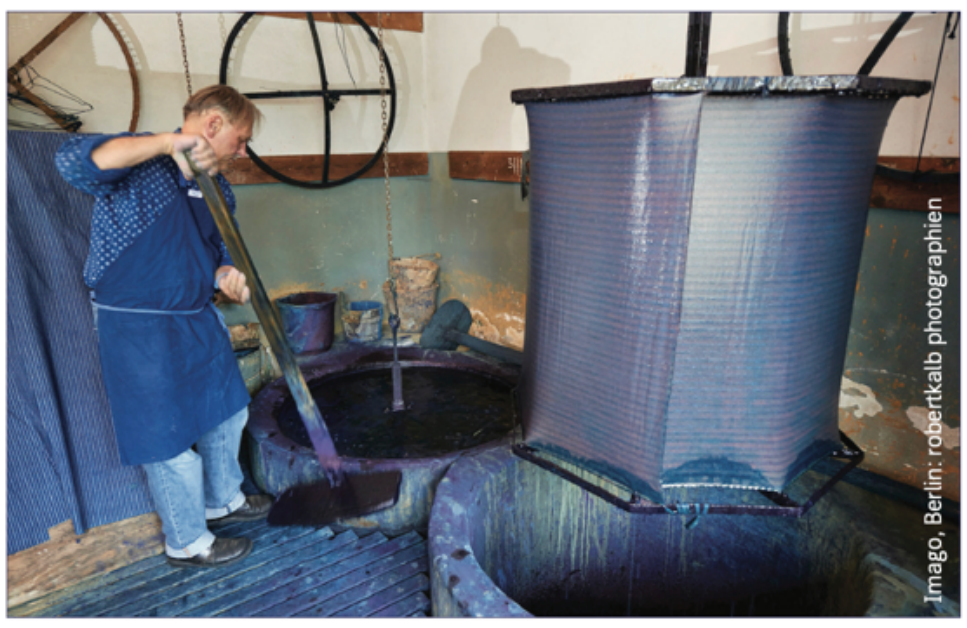

B Beispiel für immaterielles Kulturerbe: Blaudruck

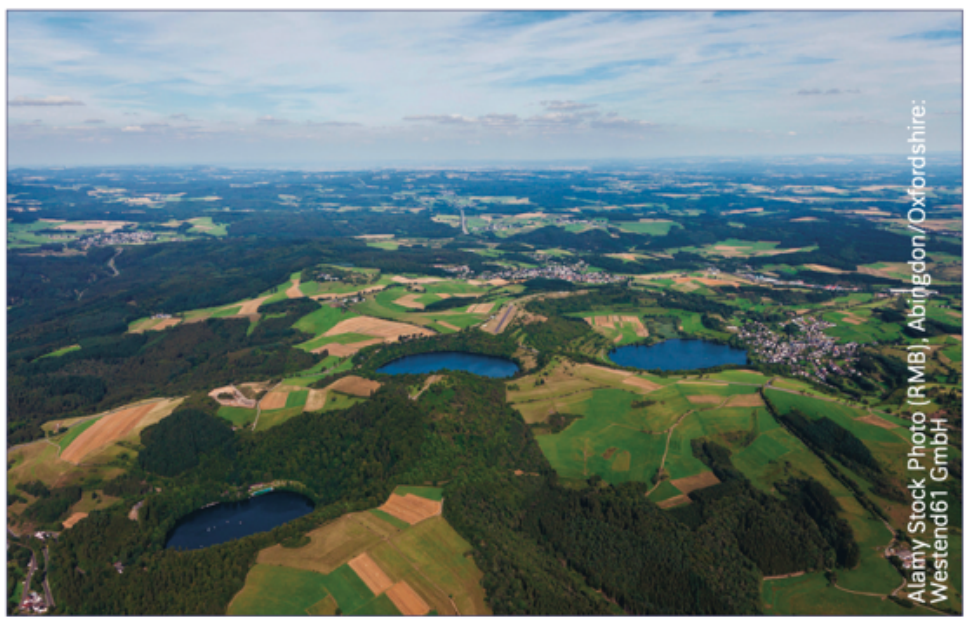

C Beispiel für einen Geopark: Vulkaneifel

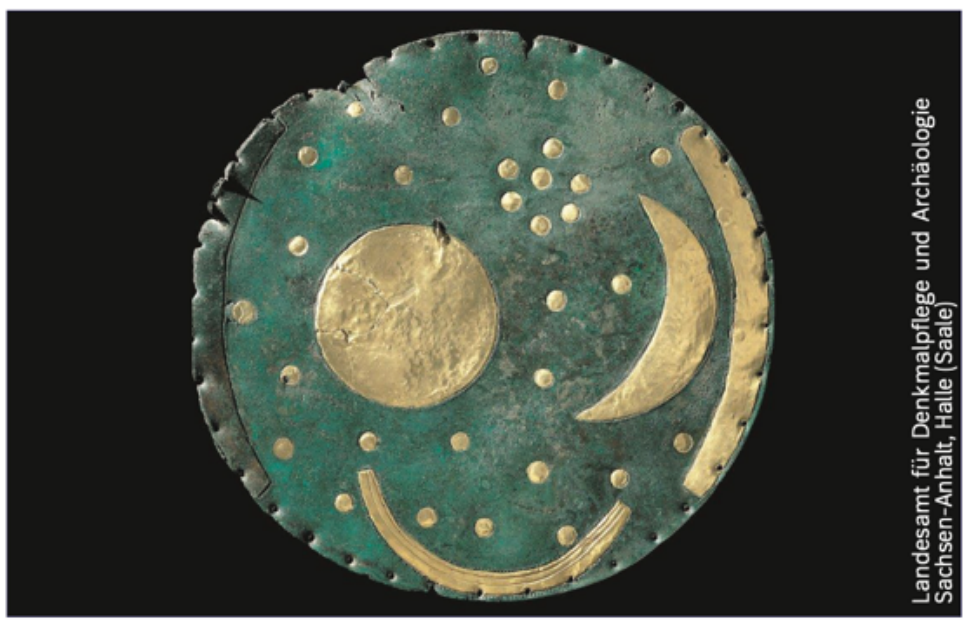

D Beispiel für Weltdokumentenerbe: Himmelsscheibe von Nebra

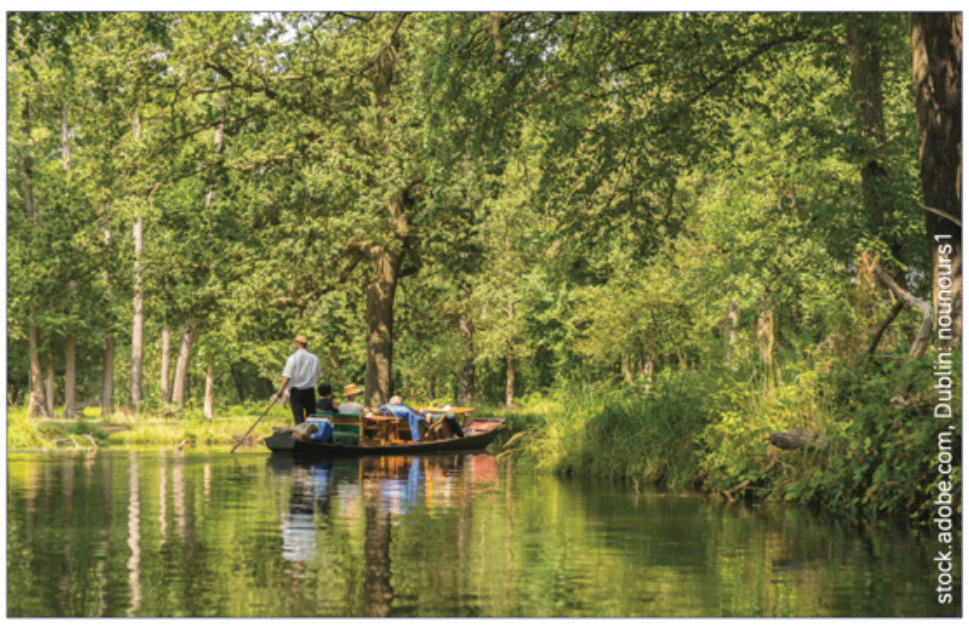

E Beispiel für ein Biosphärenreservat: Spreewald

M1 UNESCO-Erbeformen in Deutschland (Stand: April 2022)

Welterbe – Kulturerbe

1. Aachener Dom (Aachen; 1978)
2. Speyerer Dom (Speyer; 1981)
3. Würzburger Residenz und Hofgarten (Würzburg; 1981)
4. Wallfahrtskirche „Die Wies" (Steingaden; 1983)
5. Schlösser Augustusburg u. Falkenlust in Brühl (Brühl; 1984)
6. Dom und Michaeliskirche in Hildesheim (Hildesheim; 1985)
7. Römische Baudenkmäler, Dom und Liebfrauenkirche in Trier (Trier, Igel; 1986)
8. Hansestadt Lübeck (Lübeck; 1987)
9. Schlösser und Parks von Potsdam und Berlin (Berlin, Potsdam; 1990, erweitert 1992, 1999)
10. Kloster Lorsch (Lorsch; 1991)
11. Bergwerk Rammelsberg, Altstadt von Goslar und Oberharzer Wasserwirtschaft (Goslar, niedersächsischer Harz; 1992, erweitert 2010)
12. Altstadt von Bamberg (Bamberg; 1993)
13. Klosteranlage Maulbronn (Maulbronn; 1993)
14. Stiftskirche, Schloss und Altstadt von Quedlinburg (Quedlinburg; 1994)
15. Völklinger Hütte (Völklingen; 1994)
16. Kölner Dom (Köln; 1996)
17. Das Bauhaus und seine Stätten in Weimar, Dessau und Bernau (Weimar, Dessau, Bernau bei Berlin; 1996, erweitert 2017)
18. Luthergedenkstätten in Eisleben und Wittenberg (Eisleben, Wittenberg; 1996)
19. Klassisches Weimar (Weimar; 1998)
20. Museumsinsel Berlin (Berlin; 1999)
21. Wartburg (Eisenach; 1999)
22. Gartenreich Dessau-Wörlitz (Oranienbaum-Wörlitz, Dessau-Roßlau; 2000)
23. Klosterinsel Reichenau (Insel Reichenau; 2000)
24. Industriekomplex Zeche Zollverein in Essen (Essen; 2001)
25. Oberes Mittelrheintal (Rhein zwischen Bingen und Koblenz; 2002)
26. Altstädte von Stralsund und Wismar (Stralsund, Wismar; 2002)
27. Rathaus und Roland in Bremen (Bremen; 2004)
28. Muskauer Park/Park Muzakowski (Bad Muskau; 2004), mit Polen
29. Grenzen des Römischen Reiches – Obergermanisch-Raetischer Limes (von Bad Hönningen/Rheinbrohl am Rhein bis zum Kastell Eining an der Donau; Aufnahme der deutschen Stätte 2005), mit Großbritannien und Nordirland (1987, erweitert 2008)
30. Altstadt von Regensburg mit Stadtamhof (Regensburg; 2006)
31. Siedlungen der Berliner Moderne (Berlin; 2008)
32. Fagus-Werk in Alfeld (Alfeld; 2011)
33. Prähistorische Pfahlbauten um die Alpen (Alpenraum; 2011), mit Frankreich, Italien, Österreich, Schweiz, Slowenien
34. Markgräfliches Opernhaus Bayreuth (Bayreuth; 2012)
35. Bergpark Wilhelmshöhe (Kassel; 2013)
36. Karolingisches Westwerk und Civitas Corvey (Höxter; 2014)
37. Hamburger Speicherstadt und Kontorhausviertel mit Chilehaus (Hamburg; 2015)
38. Das architektonische Werk von Le Corbusier – ein herausragender Beitrag zur Moderne (Stuttgart; 2016), mit Argentinien, Belgien, Frankreich, Indien, Japan und der Schweiz
39. Höhlen und Eiszeitkunst der Schwäbischen Alb (Höhle Hohler Fels, Höhle Geißenklösterle, Sirgensteinhöhle, Bocksteinhöhle, Höhle Hohlenstein-Stadel, Vogelherdhöhle; 2017)
40. Archäologischer Grenzkomplex Haithabu und Danewerk (Busdorf; mehrere Gemeinden in den Kreisen Schleswig-Flensburg und Rendsburg-Eckernförde; 2018)
41. Naumburger Dom (Naumburg; 2018)
42. Montanregion Erzgebirge/Krušnohoří (Montan- und Bergbaulandschaften im Erzgebirge; 2019), mit Tschechien
43. Augsburger Wassermanagement-System (Augsburg; 2019)
44. Grenzen des Römischen Reiches – Donaulimes (Verlauf in Niederbayern, u. a. Regensburg; 2021), mit Österreich und der Slowakei
45. SchUM-Stätten (Mainz, Worms, Speyer; 2021)
46. Grenzen des Römischen Reiches – Niedergermanischer Limes (Haus Bürgel bei Monheim, Remagen, Xanten; 2021), mit den Niederlanden
47. Mathildenhöhe Darmstadt (Darmstadt; 2021)
48. Bedeutende Kurstädte Europas (in Deutschland: Baden-Baden, Bad Kissingen, Bad Ems; 2021), mit Belgien, Großbritannien und Nordirland, Frankreich, Italien, Österreich, Tschechien

Welterbe – Naturerbe

1. Grube Messel (Messel; 1995)
2. Wattenmeer (in Deutschland: NP Hamburgisches Wattenmeer, NP Niedersächsisches Wattenmeer, NP Schleswig-Holsteinisches Wattenmeer; 2009, erweitert 2014), mit Dänemark und den Niederlanden
3. Alte Buchenwälder und Buchenurwälder der Karpaten und anderer Regionen Europas (Jasmund, Hainich, Kellerwald-Edersee, Serrahn, Grumsin; Aufnahme der deutschen Stätten 2011), mit der Slowakei und der Ukraine (2007) sowie Albanien, Belgien, Bulgarien, Italien, Kroatien, Österreich, Rumänien, Slowenien, Spanien, Ukraine (2017)

Weltdokumentenerbe

Es ist nur der jeweils erstgenannte Ort in die Karte aufgenommen worden.

1. Tondokumente traditioneller Musik 1893–1952 (Edison-Zylinder) des Berliner Phonogrammarchivs (Berlin; 1999)
2. Gutenberg-Bibel (Göttingen; 2001)
3. Goethes literarischer Nachlass (Weimar; 2001)
4. Beethovens Neunte Sinfonie (in Deutschland: Berlin, Bonn; 2001)
5. Fritz Langs Stummfilmklassiker „Metropolis" (Wiesbaden; 2001)
6. Reichenauer Handschriften (in Deutschland: München, Bamberg, Darmstadt, Trier, Aachen)
7. Kinder- und Hausmärchen der Brüder Grimm (Kassel; 2005)
8. Waldseemüllerkarte von 1507 (Washington [USA]; 2005)
9. Renaissance-Bibliothek des Mathias Corvinus (in Deutschland: München, Wolfenbüttel; 2005)
10. Briefwechsel von Gottfried Wilhelm Leibniz (Hannover; 2007)
11. Nibelungenlied (in Deutschland: München, Karlsruhe; 2009)
12. Benz-Patent von 1886 (Fellbach; 2011)
13. Dokumente zum Bau und Fall der Berliner Mauer und der Zwei-Plus-Vier-Vertrag (Berlin, Frankfurt a. M., Potsdam, Hamburg, Kleinmachnow; 2011)
14. Lorscher Arzneibuch (Bamberg; 2013)
15. Himmelsscheibe von Nebra (Halle/Saale; 2013)
16. Die goldene Bulle (in Deutschland: Frankfurt a. M., Darmstadt, Nürnberg, München, Stuttgart; 2013)
17. Schriften von Karl Marx „Das Manifest der Kommunistischen Partei" und „Das Kapital" (Amsterdam; 2013)
18. Frühe Schriften der Reformationsbewegung (Wolfenbüttel, Dresden, Dessau, Berlin, Weimar, Gotha, Wittenberg, Heidelberg, Worms, Jena; 2015)
19. Autograph der h-Moll-Messe von Johann Sebastian Bach (Berlin; 2015)
20. Goldener Brief des birmanischen Königs Alaungphaya an den britischen König George II. (Hannover; 2015)
21. Handschriften des Buches „Al-Masaalik Wa Al-Mamaalik" (Gotha; 2015)
22. Digitale Sammlung zur sprachlichen Vielfalt (Nijmegen; 2015)
23. Verfahrensunterlagen und Tonbandaufnahmen des ersten Frankfurter Auschwitz-Prozesses (Marburg; 2017)
24. Constitutio Antoniniana (Gießen; 2017)

Geoparks

1. Bergstraße-Odenwald (2015)
2. Harz – Braunschweiger Land – Ostfalen (2015)
3. Schwäbische Alb (2015)
4. TERRA.vita (2015)
5. Vulkaneifel (2015)
6. Muskauer Faltenbogen/Łuk Mużakowa (2015), mit Polen
7. Thüringen Inselsberg – Drei Gleichen (2021)

Biosphärenreservate

1. Flusslandschaft Elbe (1979, erweitert 1997)
2. Thüringer Wald (1979, erweitert 1986, 1990, 2016, 2018)
3. Berchtesgadener Land (1990, erweitert 2010)
4. Schorfheide-Chorin (1990)
5. Schleswig-Holsteinisches Wattenmeer und Halligen (1990)
6. Rhön (1991, erweitert 2014)
7. Spreewald (1991)
8. Südost-Rügen (1991)
9. Hamburgisches Wattenmeer (1992)
10. Niedersächsisches Wattenmeer (1992)
11. Pfälzerwald-Nordvogesen (1992, erweitert 1998), mit Frankreich
12. Oberlausitzer Heide- und Teichlandschaft (1996)
13. Schaalsee (2000)
14. Bliesgau (2009)
15. Schwäbische Alb (2009)
16. Schwarzwald (2017)

Immatrielles Kulturerbe der Menschheit

Es ist nur der jeweils erstgenannte Ort in die Karte aufgenommen worden.

1. Falknerei (Seelze, Hamminkeln, Gehaus; 2016), transnational
2. Genossenschaftsidee (Delitzsch, Hachenburg; 2016)
3. Orgelbau und Orgelmusik (u. a. Karlsruhe, Laberweinting, Mettlach, Speyer; 2017)
4. Blaudruck (Jever, Einbeck, Erfurt, Pulsnitz, Lüdinghausen, Berlin, Wolsier, Coswig, Scheeßel, Ruhmannsfelden; 2018), transnational
5. Bauhüttenwesen (Aachen, Bamberg, Dresden, Freiburg, Köln, Lübeck, Mainz, Passau, Regensburg, Schwäbisch Gmünd, Soest, Ulm, Xanten; 2020)

Erklärung

4 – Wallfahrtskirche „Die Wies" – (Steingaden; 1983)

Nummer in der Karte – offizieller Name des UNESCO-Erbes – Ort(e), Region – Jahr der Ausweisung

4 – grenzüberschreitende oder transnationale Erbestätte

Betrifft ein UNESCO-Erbe mehrere Orte, so wurden aufgrund der Übersichtlichkeit in der Karte nicht immer alle dargestellt.

Betrifft ein UNESCO-Erbe auch Orte außerhalb Deutschlands, so wurden diese in der Karte nicht dargestellt.

Staatsgrenze
Landesgrenze
Stadt

0 20 40 60 80 100 km

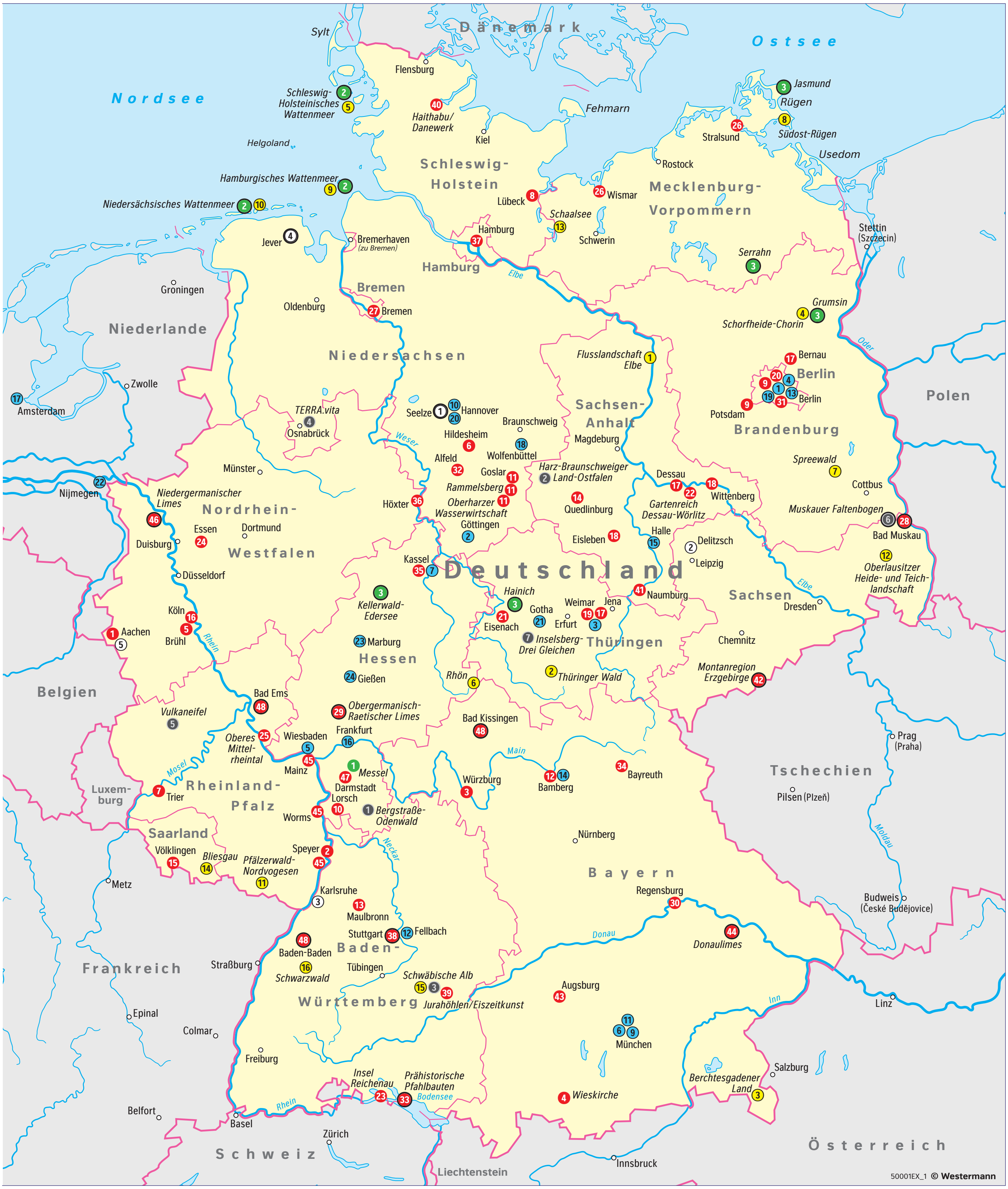
Dänemark
Ostsee
Nordsee
Sylt
Flensburg
Schleswig-Holsteinisches Wattenmeer
Haithabu/ Danewerk
Kiel
Fehmarn
Jasmund
Rügen
Südost-Rügen
Stralsund
Usedom
Helgoland
Rostock
Schleswig-Holstein
Mecklenburg-Vorpommern
Hamburgisches Wattenmeer
Niedersächsisches Wattenmeer
Lübeck
Wismar
Schaalsee
Schwerin
Stettin (Szczecin)
Jever
Bremerhaven (zu Bremen)
Hamburg
Elbe
Serrahn
Groningen
Oldenburg
Bremen
Grumsin
Schorfheide-Chorin
Niederlande
Niedersachsen
Oder
Flusslandschaft Elbe
Bernau
Berlin
Zwolle
Amsterdam
TERRA.vita
Osnabrück
Seelze
Hannover
Braunschweig
Sachsen-Anhalt
Potsdam
Polen
Brandenburg
Weser
Hildesheim
Magdeburg
Wolfenbüttel
Alfeld
Münster
Goslar
Harz-Braunschweiger Land-Ostfalen
Spreewald
Dessau
Nijmegen
Niedergermanischer Limes
Rammelsberg
Höxter
Oberharzer Wasserwirtschaft
Quedlinburg
Gartenreich Dessau-Wörlitz
Wittenberg
Cottbus
Muskauer Faltenbogen
Nordrhein-Westfalen
Essen
Dortmund
Göttingen
Halle
Eisleben
Bad Muskau
Duisburg
Delitzsch
Leipzig
Oberlausitzer Heide- und Teichlandschaft
Kassel
Deutschland
Düsseldorf
Naumburg
Sachsen
Dresden
Hainich
Kellerwald-Edersee
Weimar
Jena
Köln
Gotha
Erfurt
Eisenach
Aachen
Brühl
Rhein
Marburg
Inselsberg-Drei Gleichen
Thüringen
Chemnitz
Hessen
Montanregion Erzgebirge
Belgien
Gießen
Rhön
Thüringer Wald
Bad Ems
Vulkaneifel
Obergermanisch-Raetischer Limes
Bad Kissingen
Frankfurt
Oberes Mittelrheintal
Wiesbaden
Prag (Praha)
Main
Mosel
Mainz
Messel
Würzburg
Bamberg
Bayreuth
Tschechien
Luxemburg
Rheinland-Pfalz
Darmstadt
Trier
Lorsch
Bergstraße-Odenwald
Pilsen (Plzeň)
Worms
Saarland
Neckar
Nürnberg
Moldau
Völklingen
Bliesgau
Pfälzerwald-Nordvogesen
Speyer
Bayern
Metz
Karlsruhe
Regensburg
Budweis (České Budějovice)
Maulbronn
Donau
Fellbach
Stuttgart
Donaulimes
Baden-Baden
Baden-Württemberg
Frankreich
Straßburg
Schwarzwald
Tübingen
Schwäbische Alb
Augsburg
Jurahöhlen/Eiszeitkunst
Inn
Linz
Epinal
Colmar
München
Freiburg
Salzburg
Insel Reichenau
Prähistorische Pfahlbauten
Bodensee
Berchtesgadener Land
Wieskirche
Belfort
Basel
Zürich
Schweiz
Österreich
Innsbruck
Liechtenstein
50001EX_1 © Westermann
