# Supplementary material for: Study protocol for a quasi-experimental evaluation of the whole school approach FREI DAY
Source: Arch Public Health. 2026 Oct 13;84:222. doi: 10.1186/s13690-026-02088-7 (PMC13670517; doi:10.1186/s13690-026-02088-7)
Supplement: Supplementary file 10 [file 13690_2026_2088_MOESM6_ESM.pdf]

Supplement 6: Questionnaire for school key informants (school key informant interview)

Befragung von Schlüsselpersonen an Schulen

Farbliche Markierung, welche Personengruppen die folgenden Fragen beantworten sollen:

Rot: **NUR** Schulleitung

Grün: Schulleitung, Lehrkräfte, Schulsoziabarbeiter:in, Schulpsycholog:in. Anderes Schulpersonal, nicht-pädagogisches Schulpersonal, Schüler:innenvertretung, Mitarbeitende des Schulträgers

Blau: Schulleitung, Lehrkräfte, Schulsoziabarbeiter:in, Schulpsycholog:in. Anderes Schulpersonal, nicht-pädagogisches Schulpersonal, Schüler:innenvertretung, Elternvertreter:innen, Mitarbeitende des Schulträgers

Ohne farbliche Markierung: ALLE

Interviewhinweis

Diese Befragung ist Teil der Forschungsstudie *Gesundheit und Nachhaltigkeit an Schulen*. Es können alle an der Schule aktiven Personen, z.B. Schulleitungen, Lehrkräfte, (nicht-) pädagogisches Personal und Eltern, teilnehmen.

Mit dieser Befragung erfassen wir den Stand und die Entwicklung struktureller Faktoren (z.B. Offenheit für Innovationen, intersektorale Kooperation, Unterstützung für die Verfolgung von SDGs) an den Schulstandorten. Diese Faktoren gelten als Gelingensbedingungen für eine erfolgreiche Gestaltung gesunder und nachhaltiger Lebensweisen und Lebensbedingungen.

Insbesondere erfragen wir Ihre persönlichen Einschätzungen. Dabei gibt es kein Richtig und kein Falsch! Diese Befragung richtet sich an verschiedene Personengruppen in der Schulgemeinschaft, deswegen kann es sein, dass Sie nicht alle Fragen beantworten können. Dann geben Sie bitte „Weiß nicht“ an.

Wir **empfehlen** Ihnen für die Beantwortung der Fragen einen **Interviewtermin** mit uns auszumachen. Dabei haben Sie die Möglichkeit, jederzeit Fragen zu stellen oder Ihre Angaben näher auszuführen.

Zur Terminvereinbarung können Sie uns entweder unten Ihren Namen und Ihre E-Mail-Adresse angeben, die wir ausschließlich zur Vereinbarung eines Interviewtermins nutzen werden, oder eine E-Mail an folgende Adresse schicken: freiday@mh-hannover.de.

Ihr Name: ____________

Ihre E-Mail-Adresse: ____________

Falls Sie die Befragung online ausfüllen möchten, klicken Sie unten auf "Weiter".

Ansichtsexemplar

Abschnitt 0: Datenschutzerklärung

01. Zu Beginn möchten wir Ihnen die Studieninformationen und die Datenschutzerklärung zur Verfügung stellen. Diese finden Sie untenstehend als herunterladbare PDF-Dateien.

Die Teilnahme an der Befragung ist freiwillig. Es entstehen für Sie keine Nachteile, wenn Sie nicht daran teilnehmen. Bei Teilnahme drohen Ihnen keine Konsequenzen aufgrund Ihrer Angaben.

Akzeptieren Sie unsere Datenschutzbedingungen?

☐ Ich habe die datenschutzrechtlichen Bestimmungen gelesen und akzeptiere diese.
→ weiter zu **Frage A1**

☐ Ich stimme den datenschutzrechtlichen Bestimmungen nicht zu.
→ weiter zu **Frage 02**

Datenschutzerklärung herunterladen (PDF-Datei)
Datenschutzerklärung für Schüler:innen herunterladen (PDF-Datei)
Studieninformation herunterladen (PDF-Datei)

02. Wenn Sie den datenschutzrechtlichen Bestimmungen nicht zustimmen, können Sie nicht an der Befragung teilnehmen.
Möchten Sie Ihre Angabe ändern?

☐ Ja → zurück zu **Frage 01**

☐ Nein → weiter zu **Seite 03**

03. Wenn Sie den datenschutzrechtlichen Bestimmungen nicht zustimmen, können Sie nicht an der Befragung teilnehmen.
Sie können das Browser-Fenster nun schließen.

Ansichtsexemplar

Abschnitt A: Einstiegsfragen

A1. Für unser Forschungsprojekt ist ganz entscheidend, dass wir erkennen können, ob sich Ihre persönlichen Einschätzungen zwischen den Befragungszeitpunkten ändern. Damit wir hierfür nicht auf Ihren persönlichen Namen angewiesen sind, bitten wir Sie, uns ein dreistelliges Pseudonym wie folgt mitzuteilen.

Erste Stelle: Erster Buchstabe des Vornamens Ihrer Mutter (z.B. Maria = M, Elisabeth = E).

Zweite Stelle: Erster Buchstabe des Vornamens Ihres Vaters (z.B. Max = M, Stefan = S).

Dritte Stelle: Der Tag des Geburtsdatums Ihrer Mutter (z.B. Geburtstag am 07. Mai = 07, am 12. Oktober = 12).

Pseudonym: ____ ____ ____ ____

A2. Bitte geben Sie den vollständigen Namen Ihrer Schule an, damit wir Ihre persönlichen Einschätzungen einer konkreten Schule zuordnen können.
Schule: ____________________________

Abschnitt B: Informationen zur Person

In diesem ersten Abschnitt werden Ihnen zunächst ein paar allgemeine Fragen zu Ihrer Person gestellt.

B1. Welchem Geschlecht fühlen Sie sich zugehörig?

☐ Männlich

☐ Weiblich

☐ Nicht-binär

B2. Wie alt sind Sie?

☐ Unter 25 Jahre

☐ 25 bis 35 Jahre

☐ 36 bis 45 Jahre

☐ Über 45 Jahre

B3. Welche Funktion haben Sie an Ihrer Schule?

☐ Schulleiter:in

☐ Lehrkraft

☐ Schulsozialarbeiter:in

☐ Schulpsycholog:in

☐ Anderes pädagogisches Schulpersonal

☐ Nicht-pädagogisches Schulpersonal

☐ Schüler:innenvertretung

☐ Elternvertretung

☐ Mitglied des Schulfördervereins

☐ Mitarbeitende des Schulträgers

☐ Andere Funktion, und zwar: _______________

Ansichtsexemplar

Abschnitt H: Fragen zur Schule

In diesem Abschnitt werden Ihnen allgemeine Fragen zu Ihrer Schule gestellt.

H1. Wie viele Personen der nachfolgenden Berufsgruppen sind schätzungsweise an ihrer Schule tätig?

Bitte fassen Sie alle weiteren Personen, die nicht in die ersten drei Kategorien passen, unter sonstige Betreuungskräfte zusammen.

Berufsgruppe	Anzahl
– Lehrkräfte	________
– Lehrkräfte (Teilzeit)	________
– Referendar:innen	________
– Kräfte aus den Bereichen Sozialarbeit, Schulpsychologie, Seelsorge	________
– Sonstige Betreuungskräfte	________

H2. Wie viele Schüler:innen besuchen schätzungsweise Ihre Schule?

____

H3. Handelt es sich um eine Schule im „Sozialen Brennpunkt“?

☐ Ja

☐ Nein

Ansichtsexemplar

Abschnitt I: Fragen zu Umgang und Ausstattung der Schule

In diesem Abschnitt werden Ihnen Fragen zum Umgang an Ihrer Schule und zu der Ausstattung Ihrer Schule gestellt.

I1. Die folgenden Aussagen beziehen sich auf die Mitbestimmung der Schüler:innen an Ihrer Schule. Bitte kreuzen Sie das Zutreffende an.

	Stimme nicht zu	Stimme eher nicht zu	Stimme eher zu	Stimme zu	Weiß nicht
(A) Schüler:innen nehmen die in der Schule bestehenden Mitbestimmungsmöglichkeiten tatsächlich wahr.					
(B) In der Schule herrscht ein offenes, auf wechselseitiger Anerkennung beruhendes Klima.					
(C) Schüler:innen wollen an schulischen Entscheidungen partizipieren.					
(D) Die Schule fördert bei Schüler:innen die Überzeugung, mit schwierigen Herausforderungen fertig zu werden.					
(E) Die Schule bietet den Schüler:innen Räume, in denen sie echte Verantwortung wahrnehmen können.					
(F) Wie es in der Schule zu Entscheidungen kommt, ist nachvollziehbar.					
(G) In der Schule gibt es klare Regeln.					

Ansichtsexemplar

I2. Inwieweit beteiligen sich die Eltern an Ihrer Schule an den folgenden Aktivitäten oder nehmen folgende Möglichkeiten der Kooperation wahr? Bitte kreuzen Sie das Zutreffende an.

Eltern nehmen folgende Möglichkeiten wahr:	Stimme nicht zu	Stimme eher nicht zu	Stimme eher zu	Stimme zu	Weiß nicht
(A) Mitarbeit in Projekten, AGs, Kursen im außerunterrichtlichen Angebot.					
(B) Mitarbeit bei der Kooperation mit externen Einrichtungen.					
(C) Sammlung von Spenden für die Schule.					
(D) Mitarbeit im Förderverein.					

I3. Die folgenden Aussagen beziehen sich darauf, wie Sie im Allgemeinen das Verhalten der Lehrkräfte an Ihrer Schule im Unterricht und gegenüber den Schüler:innen wahrnehmen, beurteilen und einschätzen. Bitte kreuzen Sie das Zutreffende an.

	Stimme nicht zu	Stimme eher nicht zu	Stimme eher zu	Stimme zu	Weiß nicht
(A) Viele Kolleg:innen achten die Meinung der Schüler:innen auch dann, wenn diese nicht ganz ausgegoren ist.					
(B) Die Kolleg:innen ermuntern die Schüler:innen häufig, die eigenen Gedanken zu äußern, auch wenn sie falsch sein könnten.					
(C) Den meisten Kolleg:innen ist es wichtiger, dass die Schüler:innen gute Ideen haben, als dass sie viel wissen.					
(D) Vielen Lehrkräften fällt es schwer, sich mit den Meinungen der Schüler:innen ernsthaft auseinanderzusetzen.					

I4. Wie beurteilen Sie folgende Aussagen über das Verhältnis zwischen Schüler:innen und Lehrkräften? Bitte kreuzen Sie das Zutreffende an.

	Stimme nicht zu	Stimme eher nicht zu	Stimme eher zu	Stimme zu	Weiß nicht
(A) Schüler:innen und Lehrkräfte kommen meistens gut miteinander aus.					
(B) Den meisten Lehrkräften ist es wichtig, dass die Schüler:innen sich wohlfühlen.					
(C) Die meisten Lehrkräfte interessieren sich für das, was die Schüler:innen zu sagen haben.					
(D) Wenn ein:e Schüler:in zusätzliche Hilfe braucht, bekommt er/sie sie von der Lehrkraft.					
(E) Die Lehrkräfte behandeln die Schüler:innen fair.					

Ansichtsexemplar

I5. Nun geht es darum, wie Sie Ihre Schüler:innen sowie deren Umgang untereinander einschätzen. Inwiefern stimmen Sie den folgenden Aussagen zu?

**Unsere Schüler:innen**	Stimme nicht zu	Stimme eher nicht zu	Stimme eher zu	Stimme zu	Weiß nicht

(A) ... erkennen einander als gleichberechtigt an.					
(B) ... sind in der Lage, ihre Meinungen zu begründen.					
(C) ... erkennen an, dass es zu einer Sache mehrere Meinungen geben kann.					
(D) ... sind im Umgang miteinander fair und tolerant.					
(E) ... sind in der Lage, bei gemeinsamen Entscheidungen verschiedene Meinungen anzuhören.					
(F) ... übernehmen Verantwortung, wenn sie eine Gelegenheit dazu erkennen.					
(G) ... sind dem Leben gegenüber positiv eingestellt.					
(H) ... sind selbstsicher in Gruppen.					
(I) ... sind bereit, Normen, die für den Unterricht gelten, einzuhalten.					
(J) ... sind in der Lage, Kritik anzunehmen.					
(K) … sind in der Lage, Kritik auf faire Weise ausdrücken.					
(L) ... können sich in die Sichtweise anderer hineinversetzen.					
(M) ... können einander zuhören.					
(N) ... können ihre Bedürfnisse und Gefühle einander mitteilen.					
(O) ... beherrschen grundlegende Konfliktregeln.					
(P) ... können damit umgehen, dass andere von ihnen verschieden sind.					
(Q) ... sind teamfähig.					
(R) ... verfügen über ein altersangemessenes Gerechtigkeitsverständnis.					

Ansichtsexemplar

Abschnitt J: Fragen zu Sustainable Development Goals

In diesem Abschnitt werden Ihnen Fragen zu den Sustainable Development Goals (SDGs) und deren Umsetzung an Ihrer Schule gestellt.

J1. Manche Schulen leisten gezielt Beiträge zu den SDGs. Inwiefern griff Ihrer Meinung nach Ihre Schule im letzten Jahr die einzelnen SDGs mit Projekten oder Maßnahmen auf?

	Gar nicht	Wenig	Etwas	Sehr	Weiß nicht
SDG 1: Keine Armut					
SDG 2: Kein Hunger					
SDG 3: Gesundheit und Wohlergehen					
SDG 4: Inklusive, gerechte und hochwertige Bildung					
SDG 5: Geschlechter-Gerechtigkeit					
SDG 6: Sauberes Wasser und Sanitäre Einrichtungen					
SDG 7: Bezahlbare und saubere Energie					
SDG 8: Menschenwürdige Arbeit und Wirtschaftswachstum					
SDG 9: Industrie, Innovation und Infrastruktur					
SDG 10: Weniger Ungleichheiten					
SDG 11: Nachhaltige Städte und Gemeinden					
SDG 12: Nachhaltiger Konsum und nachhaltige Produktion					
SDG 13: Maßnahmen zum Klimaschutz					
SDG 14: Nachhaltiger Umgang mit Meeren, Seen und Flüssen					
SDG 15: Nachhaltiger Umgang mit Fauna und Flora					
SDG 16: Frieden, Gerechtigkeit und starke Institutionen					
SDG 17: Partnerschaften zur Erreichung der Ziele					

Ansichtsexemplar

J2. Hat Ihre Schule die SDGs oder Bildung für nachhaltige Entwicklung teilweise oder vollständig in ihr (schriftliches) Leitbild integriert?

☐ Ja, vollständig

☐ Ja, teilweise

☐ Nein, aber wir denken darüber nach

☐ Nein, gar nicht

☐ Weiß nicht

J3. Die SDGs sind nicht nur für Schulen ein neues und vielleicht umstrittenes Thema: Inwiefern stimmen Sie den folgenden Aussagen zu den Menschen in Ihrer Kommune zu?

	Stimme nicht zu	Stimme eher nicht zu	Stimme eher zu	Stimme zu	Weiß nicht
(A) Die meisten denken, dass die SDGs eher ideologisch als sinnvoll sind.					
(B) Die meisten denken, dass Schulen aktiv SDGs verfolgen sollten.					

(C) Die meisten denken, dass Schulen für die Erreichung von SDGs mit verantwortlich sind.					
(D) Die meisten denken, dass Schulen <u>kaum</u> etwas zur Erreichung von SDGs beitragen können.					

J4. Es gibt verschiedene Faktoren, die das Bewusstsein der Kinder und Jugendlichen für nachhaltige Entwicklungen beeinflussen. Bitte geben Sie an, welchen Faktor Sie als wichtiger erachten. Dazu kann der Schieberegler nach links oder rechts verschoben werden.

(A1) Eigene Professionskompetenz bzgl. Fachwissen		(A2) Eigene Professionskompetenz bzgl. Lernprozessen
(B1) Jahrgangs-übergreifendes Lernen		(B2) Klassenspezifische Projekte
(C1) Außerschulische Akteur:innen sollten eingebunden werden		(C2) Innerschulische Umsetzung ist ausreichend
(D1) Möglichkeiten, aktiv für die SDGs zu handeln		(D2) Erkenntnisse über die SGDs gewinnen
(E1) Fachliche Kompetenzen von Kindern und Jugendlichen		(E2) Überfachliche Kompetenzen von Kindern und Jugendlichen
(F1) Freie Gestaltungs-räume als fester Bestandteil der schulischen Bildung		(F2) Projektarbeit im Unterricht

Ansichtsexemplar

J5. Wenn Schulen für SDGs aktiv werden, binden sie dafür häufig andere Personengruppen und Organisationen ein. Bitte schätzen Sie ein, inwiefern Ihre Schule im letzten Jahr mit den folgenden Gruppen/Organisationen zur Verfolgung von SDGs zusammengearbeitet hat.

	Nie	Einmal	Gelegen tlich	Dauer-haft	Weiß nicht
(A) Lokale Unternehmen					
(B) Sozialversicherungsträger wie Krankenkassen, Unfallkassen					
(C) Jugendhilfe / Jugendamt					
(D) Behörden					
(E) Universitäten, Hochschulen					
(F) Schulen					
(G) Außerschulische BNE-Standorte					
(H) Umwelt- und Wohlfahrtsorganisationen					
(I) Zivilgesellschaftliche Initiativen					
(J) Vereine (z. B. Sportvereine)					
(K) Sonstige:					

J6. Auch auf lokaler Ebene kann es sinnvoll sein, zur Verfolgung der SDGs organisations- und sektorenübergreifend zusammenzuarbeiten. Inwiefern stimmen Sie den folgenden Aussagen über Ihre Kommune zu?

	Stimme nicht zu	Stimme eher nicht zu	Stimme eher zu	Stimme zu	Weiß nicht
(A) Es gibt ein lokales Netzwerk, das sich damit beschäftigt, wie die Kommune zu den SDGs beiträgt.					
(B) Lokale Organisationen nehmen an Runden Tischen o. Ähnlichem zu SDGs teil.					
(C) Lokale Organisationen koordinieren ihre SDG-Aktivitäten selten untereinander.					
(D) Lokale Organisationen arbeiten bezüglich der SDGs zusammen.					
(E) Lokale Organisationen teilen sich Ressourcen für ihre SDG-Aktivitäten.					
(F) Lokale Organisationen verknüpfen ihre Strategien zu den SDGs.					
(G) Lokale Organisationen beteiligen sich an einer kommunalen SDG-Strategie.					
(H) Lokale Organisationen tauschen für die kommunale SDG-Strategie Daten/ Informationen aus.					
(I) Alle Beteiligten haben eine klare Rolle bei der Umsetzung der kommunalen SDG-Strategie.					

Ansichtsexemplar

J7. Nun geht es darum, wie die SDGs an Ihrer Schule umgesetzt werden. Inwiefern stimmen Sie den folgenden Aussagen zu Ihrer Schule zu?

	Stimme nicht zu	Stimme eher nicht zu	Stimme eher zu	Stimme zu	Weiß nicht
(A) Es gibt an der Schule ein aktives Netzwerk an Personen, das sich mit der Verfolgung von SDGs befasst.					
(B) Der meiste Teil des pädagogischen Personals unserer Schule glaubt, dass sie wesentlich zur Erreichung von SDGs beitragen kann.					
(C) Die meisten Lehrkräfte wissen gut über die Aktivitäten der Schule zur Verfolgung von SDGs Bescheid.					

(D) Das Schulpersonal koordiniert nur <u>selten</u> seine Aktivitäten zur Verfolgung von SDGs.					
(E) Die Schule ist bereit, neue Ideen für die Verfolgung von SDGs auszuprobieren.					
(F) Das Personal arbeitet zusammen, um SDGs effektiv in der Schule und im Unterricht zu verfolgen.					
(G) Das Personal ist in seinen Routinen zu festgefahren, um SDGs zeitnah in seine Routinen zu integrieren.					
(H) Schule und Personal engagieren sich gezielt für SDGs in und außerhalb der Schule.					

J8. Haben sich Ihrer Meinung nach bei folgenden Aspekten in den letzten 2 Jahren Veränderungen ergeben?

	Deutlich verringert	Eher verringert	Nein	Eher erhöht	Deutlich erhöht	Weiß nicht
(A) Das Ausmaß der Aktivitäten an unserer Schule zu den SDGs.						
(B) Die Höhe der Ressourcen (z.B. Personal, Finanzen) für SDG-Maßnahmen an unserer Schule.						
(C) Die Lernkultur/ der Unterricht.						
(D) Die Partizipationsbereitschaft der Lehrkräfte.						
(E) Die Partizipationsbereitschaft der Schüler:innen.						

Ansichtsexemplar

Abschnitt K: Fragen zum FREI DAY

K0. Wird an Ihrer Schule das Lernformat FREI DAY umgesetzt?

☐ Ja

☐ Nein → weiter zu **Abschnitt L**

In diesem letzten Abschnitt werden Ihnen Fragen zum Lernformat FREI DAY von der Initiative Schule im Aufbruch gestellt.

K1. Haben Sie an Ihrer Schule ein Netzwerk oder Team, dass sich mit der Umsetzung des FREI DAY befasst?

☐ Ja

☐ Nein → weiter zu **Abschnitt L**

☐ Weiß nicht → weiter zu **Abschnitt L**

K2. Sind Ihrer Meinung nach alle Interessensgruppen der Schule (auch Minderheiten) an den Planungs- und Entscheidungsprozessen dieses FREI-DAY-Netzwerks/-Teams angemessen beteiligt?

☐ Ja, vollständig

☐ Ja, teilweise

☐ Nein, aber wir denken darüber nach

☐ Nein, gar nicht

☐ Weiß nicht

K3. Inwieweit waren die folgenden Personengruppen an der Einführung des FREI DAY an Ihrer Schule beteiligt?

	Nie	Eher selten	Eher häufig	Häufig	Weiß nicht
1. Die Schulleitung					
2. Einzelne Lehrkräfte auf freiwilliger Basis					
3. Schulsozialarbeiter:innen					
4. Weiteres pädagogisches Personal					
5. Eigens eingerichtetes „Schulentwicklungsteam" aus dem Kollegium					
6. Andere Gruppen oder Gremien des Kollegiums					
7. Das gesamte Kollegium					
8. Die Fachkonferenzen					
9. Die Schüler:innenvertretung					
10. Die Elternvertretretung					
11. Berater:in von Schule im Aufbruch					
12. Andere:r externe:r Berater:in, z.B. Schulentwicklungsberater:in					

K4. Bitte denken Sie an das letzte Jahr: Inwieweit wurde Ihrer Meinung nach die Umsetzung des FREI DAY durch folgende Faktoren negativ beeinflusst?

	Gar nicht	Wenig	Viel	Sehr viel	Weiß nicht
a. Mangelnde Zusammenarbeit der Beteiligten					
b. Mangelnde Übereinstimmung bei Zielen und Methoden					
c. Mangelnde Führung / Moderation					
d. Verlust beteiligter Schlüsselpersonen					
e. Nicht nachhaltige oder mangelnde finanzielle Ressourcen					

Ansichtsexemplar

f. Mangelnde personelle Ressourcen					
g. Mangelnde Unterstützung durch das Schulpersonal					
h. Widerstände beim Schulpersonal					
i. Mangelnde Unterstützung durch die Eltern					
j. Widerstände bei den Eltern					
k. Mangelnde Unterstützung durch die Schüler:innen					
l. Widerstände bei den Schüler:innen					

K5. An wie vielen Schultagen pro Monat sollte aus Ihrer Sicht nach dem FREI-DAY-Ansatz gearbeitet werden?

__ __ Tagen von 28 Schultagen / Monat

Ansichtsexemplar

K6. Inwieweit stimmen Sie den folgenden Aussagen zum FREI DAY an Ihrer Schule zu?

	Stimme nicht zu	Stimme eher nicht zu	Stimme eher zu	Stimme zu	Weiß nicht
(A) Die Arbeit am FREI DAY bedeutet eine zusätzliche Belastung für unsere Schule.					
(B) Der FREI DAY hat wichtige Impulse für unsere Schule gegeben.					
(C) Das Nachdenken über gemeinsame pädagogische Ziele hat den Unterricht der einzelnen Lehrkräfte verändert.					
(D) An unserer Schule gab es Unmut über die zusätzliche Arbeitsbelastung.					
(E) Die Verständigung auf gemeinsame Ziele ist für die Unterrichtsarbeit der einzelnen Lehrkräfte enorm wichtig.					
(F) Die Gestaltungsmöglichkeiten der einzelnen Lehrkräfte in Bezug auf ihren Unterricht sind durch den FREI DAY eingeschränkt.					

K7. Haben Sie einen Schlüsselmoment in Bezug auf den FREI DAY? Ein Erlebnis, das sie besonders nachhaltig berührt hat oder Ihnen ganz besonders im Gedächtnis geblieben ist?

Ansichtsexemplar

Abschnitt L: Weiterführende Kontaktdaten

Um das Geschehen an Ihrer Schule möglichst umfassend darstellen zu können, möchten wir systematisch Personen, die Ihre Schule gut einschätzen können, befragen. Gemeint sind hier nicht nur Personen, die an Ihrer Schule tätig sind, sondern ebenso Eltern, Schüler:innen oder auch Mitarbeitende der Kommune bzw. des Schulträgers.

Würden Sie uns bitte für zwei Personen die Kontaktdaten nennen?

Art. 14 der DSGVO erlaubt es wissenschaftlichen Studien, personenbezogene Daten über Dritte zu erfragen.

Alternativ können Sie sich auch erst absprechen und uns im Nachhinein Kontaktdaten zusenden. Nutzen Sie dafür bitte folgende E-Mail-Adresse: freiday@mh-hannover.de

L1. Kontaktdaten der ersten Person.

Name: ____________________________

Telefon: ____________________________

E-Mail-Adresse: ____________________________

L2. Bitte nennen Sie uns die Funktion der ersten Person.

☐ Lehrkraft

☐ Schulsozialarbeitskraft

☐ Schulpsychologische Fachkraft

☐ Schüler:innenvertretung

☐ Elternvertretung

☐ Mitglied des Schulfördervereins

☐ Mitarbeitende des Schulträgers

☐ Externe Kooperationspartner:in

☐ Andere Funktion, und zwar: _________________

☐ Weiß nicht

L3. Kontaktdaten von der zweiten Person.

Name: ____________________________

Telefon: ____________________________

E-Mail-Adresse: ____________________________

L4. Bitte nennen Sie uns die Funktion der zweiten Person.

☐ Lehrkraft

☐ Schulsozialarbeitskraft

☐ Schulpsychologische Fachkraft

☐ Schüler:innenvertretung

☐ Elternvertretung

☐ Mitglied des Schulfördervereins

(Fortsetzung Frage L4)

Ansichtsexemplar

☐ Mitarbeitende des Schulträgers
☐ Externe Kooperationspartner:in
☐ Andere Funktion, und zwar: _______________
☐ Weiß nicht

Abschluss

Vielen Dank für Ihre Teilnahme!
Wir möchten uns ganz herzlich für Ihre Mithilfe bedanken.
Ihre Antworten wurden gespeichert, Sie können das Browser-Fenster nun schließen.

Ansichtsexemplar
