# Supplementary material for: Study protocol for a quasi-experimental evaluation of the whole school approach FREI DAY
Source: Arch Public Health. 2026 Oct 13;84:222. doi: 10.1186/s13690-026-02088-7 (PMC13670517; doi:10.1186/s13690-026-02088-7)
Supplement: Supplementary file 11 [file 13690_2026_2088_MOESM7_ESM.pdf]

Supplement 7: Questionnaire for school board (school board interview)

Befragung des FREI DAY-Schulteams

Abschnitt 0: Datenschutzerklärung

01. Diese Befragung ist Teil der Evaluation des Lernformats "FREI DAY". Mit dieser Befragung erfassen wir, welche Faktoren für eine erfolgreiche Umsetzung des FREI DAY und für die Zusammenarbeit im FREI-DAY-Schulentwicklungsteam (im Folgenden FREI-DAY-Team genannt) relevant sind.

Insbesondere erfragen wir Ihre persönlichen Einschätzungen zur Zusammenarbeit, Teamfunktionalität, Einstellung und Koordination des FREI-DAY-Teams, zu hemmenden und fördernden Rahmenbedingungen an Ihrer Schule sowie zur Unterstützung durch die Schule im Aufbruch gGmbH.

Alle Mitglieder Ihres FREI-DAY-Teams werden jährlich innerhalb der nächsten drei Jahre befragt.

Zu Beginn möchten wir Ihnen die Studieninformationen und die Datenschutzerklärung zur Verfügung stellen. Diese finden Sie untenstehend als herunterladbare PDF-Dateien.

Die Teilnahme an der Befragung ist freiwillig. Es entstehen für Sie keine Nachteile, wenn Sie nicht daran teilnehmen. Bei Teilnahme drohen Ihnen keine Konsequenzen aufgrund Ihrer Angaben.

Akzeptieren Sie unsere Datenschutzbedingungen?

☐ Ich habe die datenschutzrechtlichen Bestimmungen gelesen und akzeptiere diese.
  → weiter zu **Frage A1**

☐ Ich stimme den datenschutzrechtlichen Bestimmungen nicht zu.
  → weiter zu **Frage 02**

Datenschutzerklärung herunterladen (PDF-Datei)
Studieninformation herunterladen (PDF-Datei)

02. Wenn Sie den datenschutzrechtlichen Bestimmungen nicht zustimmen, können Sie nicht an der Befragung teilnehmen.
Möchten Sie Ihre Angabe ändern?

☐ Ja → zurück zu **Frage 01**

☐ Nein → weiter zu **Seite 03**

03. Wenn Sie den datenschutzrechtlichen Bestimmungen nicht zustimmen, können Sie nicht an der Befragung teilnehmen.
Sie können das Browser-Fenster nun schließen.

Ansichtsexemplar

Abschnitt A: Einstiegsfragen

A1. Für unser Forschungsprojekt ist ganz entscheidend, dass wir erkennen können, ob sich Ihre persönlichen Einschätzungen zwischen den Befragungszeitpunkten ändern. Damit wir hierfür nicht auf Ihren persönlichen Namen angewiesen sind, bitten wir Sie, uns ein dreistelliges Pseudonym wie folgt mitzuteilen.

Erste Stelle: Erster Buchstabe des Vornamens Ihrer Mutter (z.B. Maria = M, Elisabeth = E).

Zweite Stelle: Erster Buchstabe des Vornamens Ihres Vaters (z.B. Max = M, Stefan = S).

Dritte Stelle: Der Tag des Geburtsdatums Ihrer Mutter (z.B. Geburtstag am 07. Mai = 07, am 12. Oktober = 12).

Pseudonym: ____ ____ ____ ____

A2. Bitte geben Sie den vollständigen Namen Ihrer Schule an, damit wir Ihre persönlichen Einschätzungen einer konkreten Schule zuordnen können.

Schule: ____________________________

Ansichtsexemplar

School Board Interviews (SBI)

Abschnitt B: Informationen zur Person

In diesem ersten Abschnitt werden Ihnen zunächst ein paar allgemeine Fragen zu Ihrer Person gestellt.

B1. Welchem Geschlecht fühlen Sie sich zugehörig?

☐ Männlich
☐ Weiblich
☐ Nicht-binär

B2. Wie alt sind Sie?

☐ Unter 25 Jahre
☐ 25 bis 35 Jahre
☐ 36 bis 45 Jahre
☐ Über 45 Jahre

B3. Welche Funktion haben Sie an Ihrer Schule?

☐ Schulleitung
☐ Lehrkraft
☐ Schulsozialarbeiter:in
☐ Schulpsycholog:in
☐ Sonstiges pädagogisches Schulpersonal
☐ Nicht-pädagogisches Schulpersonal
☐ Elternvertreter:innen
☐ Mitglied des Schulfördervereins
☐ Mitarbeitende des Schulträgers
☐ Andere Funktion, und zwar: _______________

Ansichtsexemplar

Abschnitt C: Allgemeine Fragen zum FREI DAY

C1. Wird an Ihrer Schule das Lernformat FREI DAY umgesetzt?

☐ Ja

☐ Nein → weiter zu **School Key Informant Interview**

C2. In welcher der acht FREI DAY-Phasen befindet sich Ihre Schule bei der Einführung bzw. Umsetzung des FREI DAY? Bitte kreuzen Sie die entsprechende Phase an.

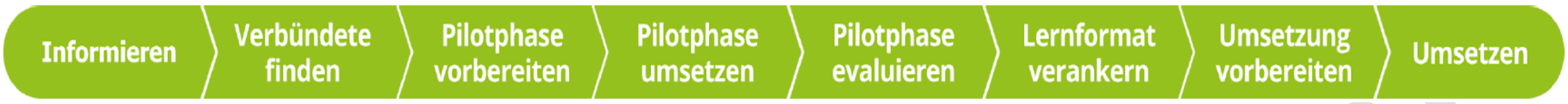

☐ Phase 1: Informieren	☐ Phase 2: Verbündete finden	☐ Phase 3: Pilotphase vorbereiten	☐ Phase 4: Pilotphase umsetzen	☐ Phase 5: Pilotphase evaluieren	☐ Phase 6: Lernformat verankern	☐ Phase 7: Umsetzung vorbereiten	☐ Phase 8: Umsetzen

☐ Weiß nicht

C3. Gibt es an Ihrer Schule ein FREI-DAY-Schulentwicklungsteam (auch FREI-DAY-Team genannt) und gehören Sie diesem FREI-DAY-Team an?

☐ Ja

☐ Nein → weiter zu **Frage C4**, ohne Item (E) und (G)

C4. Inwiefern treffen Ihrer Meinung nach die folgenden Aussagen zum FREI DAY und zum FREI-DAY-Team Ihrer Schule zu?

	Trifft nicht zu	Trifft eher nicht zu	Trifft eher zu	Trifft zu	Weiß nicht
(A) Der FREI DAY ist schwer umzusetzen.					
(B) Der FREI DAY ist mit den Werten und Überzeugungen unserer Schule vereinbar.					
(C) Der FREI DAY wird für unsere Schule mehr Ressourcen für die Auseinandersetzung mit den SDGs generieren.					
(D) Durch den FREI DAY wird die Kommunikation und Zusammenarbeit an unserer Schule und mit Einrichtungen in der Region verbessert.					
(E) Unser FREI-DAY-Team ist für die Durchführung des FREI DAY an unserer Schule hilfreich.					
(F) Ich stehe hinter dem Bildungsansatz des FREI DAY.					
(G) Unser FREI-DAY-Team greift wichtige Belange an unserer Schule auf, die sonst zu kurz kämen.					

Ansichtsexemplar

Abschnitt D: Fragen zum FREI-DAY-Team

D1. Wer nimmt regelmäßig am FREI-DAY-Team teil?

☐ Schüler:innen
☐ Eltern
☐ Lehrkräfte
☐ Schulsozialarbeiter:innen
☐ Schulpsycholog:innen
☐ Weitere pädagogische Fachkräfte
☐ Schulleitung
☐ andere: ________________________

D2. Wie häufig finden Treffen des FREI-DAY Teams statt?

☐ wöchentlich
☐ zweimal im Monat
☐ monatlich
☐ alle zwei Monate
☐ vierteljährlich
☐ andere: ________________________
☐ Weiß nicht

D3. Wie lange dauert ein Treffen des FREI-DAY-Teams üblicherweise?

__ __ Zeitstunden ☐ Weiß nicht

D4. Wie viele Zeitstunden verbringen Sie durchschnittlich mit der Vor-/Nachbereitung eines Treffens des FREI-DAY-Teams?

__ __ Zeitstunden ☐ Weiß nicht

D5. Wie viele Mitglieder sind in Ihrem FREI-DAY-Team?

__ __ Anzahl der Mitglieder ☐ Weiß nicht

D6. Im Folgenden geht es zunächst darum, wie weit der Aufbau eines wirksamen FREI-DAY-Teams an Ihrer Schule fortgeschritten ist.

	Nein	Nein, aber in Arbeit	Ja, teilweise	Ja	Weiß nicht
(A) Konnte sich das Team darüber einigen, wie es arbeiten und Entscheidungen treffen möchte?					
(B) Hat das Team klare Ziele und Grundsätze entwickelt?					
(C) Hat das Team einen Plan, wie es neue Mitglieder über die Vision, Aufgaben, Ziele					

Ansichtsexemplar

	Nein	Nein, aber in Arbeit	Ja, teilweise	Ja	Weiß nicht
und Arbeitsweise des FREI-DAY-Teams informiert?					
(D) Hat das Team seine Stärken und die der einzelnen Mitglieder identifiziert und nutzt diese?					
(E) Besitzt das Team bereits alle nötigen Kompetenzen, um den FREI DAY in der Schule zu verstetigen?					
(F) Hat das Team eine Strategie dafür, wie FREI-DAY-Projekte finanzielle Mittel erhalten?					
(G) Hat das Team einen detaillierten Plan, wie es die Effekte des FREI DAY vor Ort überprüft und bewertet?					

D7. Inwiefern treffen Ihrer Meinung nach die folgenden Aussagen zum FREI-DAY-Team an Ihrer Schule zu?

	Trifft nicht zu	Trifft eher nicht zu	Trifft eher zu	Trifft zu	Weiß nicht
(A) Die Mitglieder unseres Teams kommen zu unseren Treffen.					
(B) Alle Team-Mitglieder beteiligen sich an den Diskussionen, nicht nur einzelne.					
(C) Unsere Team-Mitglieder verstehen ihre Rolle bei der Einführung des FREI DAY an unserer Schule.					
(D) Unser Team ist durch ein Gefühl der Einheit und des Zusammenhalts geprägt.					
(E) Unser Team hatte eine schwere Phase, in der Konflikte gelöst werden mussten.					
(F) Unser Team nutzt die Fähigkeiten von allen Mitgliedern, nicht nur von einzelnen.					
(G) Es besteht kein starkes Zusammengehörigkeitsgefühl in unserem Team.					
(H) Es ist schwer, ein Gefühl von Vertrauen mit den anderen Mitgliedern unseres Teams aufzubauen.					
(I) Neue Team-Mitglieder werden in den FREI DAY eingeführt.					
(J) Die Einbindung neuer Mitglieder erfolgt strukturiert.					

Ansichtsexemplar

D8. Inwiefern treffen Ihrer Meinung nach die folgenden Aussagen über die Effektivität Ihres FREI-DAY-Teams zu?

	Trifft nicht zu	Trifft eher nicht zu	Trifft eher zu	Trifft zu	Weiß nicht
(A) Unser Team ist organisiert und effizient.					
(B) Alle Mitglieder unseres Teams arbeiten intensiv mit.					
(C) Unser Team ist sehr ergebnisorientiert.					
(D) In unserem Team streiten wir uns über Ziele und Inhalte.					
(E) Die „richtigen“ Personen sind am Team beteiligt (d. h. Personen mit den benötigten Fähigkeiten, Begabungen und außerschulischen Verbindungen).					
(F) Unser Team ist fähig, vom Reden zum Handeln überzugehen.					
(G) Unser Team verschwendet viel Zeit mit Unnötigem.					
(H) Unser Team kann <u>selten</u> etwas Konkretes in Bezug auf seine Anstrengungen vorweisen.					

Ansichtsexemplar

D9. Manche FREI-DAY-Teams müssen mit Konflikten umgehen (z. B. divergierende Ansichten, persönliche Fehden, Machtkämpfe). Wie viele Konflikte haben Sie in Ihrem FREI-DAY-Team wahrgenommen?

☐ viele
☐ einige
☐ wenige
☐ keine
☐ weiß nicht

D10. Ist die Zusammensetzung Ihres FREI-DAY-Teams stabil oder gibt es viele Mitgliederwechsel?

☐ instabil
☐ eher instabil
☐ eher stabil
☐ stabil
☐ weiß nicht

D11. Im Folgenden geht es uns um Ihre persönliche Bilanz aus Ihrer Mitarbeit im FREI-DAY-Team an Ihrer Schule. Inwiefern treffen die folgenden Aussagen auf Sie persönlich zu?

	Trifft nicht zu	Trifft eher nicht zu	Trifft eher zu	Trifft zu	Weiß nicht
(A) Meine organisatorischen Kompetenzen haben sich durch die Mitarbeit verbessert.					
(B) Meine kommunikativen Kompetenzen haben sich durch die Mitarbeit verbessert.					
(C) Meine Kompetenzen zur Konzeption oder Durchführung von FREI-DAY-Projekten haben sich durch die Mitarbeit verbessert.					
(D) Meine Möglichkeit, die Agenda unserer Schule mitzugestalten, hat sich durch die Mitarbeit verbessert.					
(E) Meine Ziele und Aktivitäten werden jetzt von anderen wahrgenommen und mehr unterstützt.					
(F) Mir gibt es ein gutes Gefühl, unsere Schule mit unserem Team zu stärken.					
(G) Aufgrund meiner Arbeit im Team bin ich jetzt mit anderen besser vernetzt.					
(H) Meine Arbeit im Team wird wahrgenommen und wertgeschätzt.					
(I) Durch meine Beteiligung im Team sind meine Arbeitsabläufe aufwendiger geworden.					
(J) Ich kann meiner Rolle im Team derzeit häufig nicht ausreichend gerecht werden.					
(K) Ich fühle mich durch meinen Einsatz im Team häufiger gestresst.					
(L) Meine Arbeit im Team beeinträchtigt meine Freizeit.					

Ansichtsexemplar

Abschnitt E: Fragen zur Lernbegleitung

E1. Sind Sie als Lernbegleiter:in beim FREI DAY aktiv?

☐ Ja

☐ Nein → weiter mit **Frage E7**

E1.1 Haben Sie an einer Fortbildung für Lernbegleiter:innen teilgenommen?

☐ Nein

☐ Ja, im Laufe der letzten 12 Monate

☐ Ja, vor längerer Zeit

E1.2 Wie viele Stunden haben Sie für die Fortbildung benötigt (inklusive Vor- und Nachbereitung)?

_____ Zeitstunden

E1.3 Wie lange sind Sie schon als Lernbegleiter:in tätig?

____ Jahre

E2. Inwieweit treffen folgende Aussagen auf Ihre Rolle als Lernbegleiter:in zu?

	Trifft nicht zu	Trifft eher nicht zu	Trifft eher zu	Trifft zu	Weiß nicht
(A) Mir gibt es ein gutes Gefühl, unsere Schule durch die Begleitung unserer FREI-DAY-Projekte zu stärken.					
(B) In der Rolle fühle ich mich wohl.					
(C) Die Rolle sehe ich am ehesten als Ergänzung zu meiner sonstigen Lehrkraftrolle.					
(D) Die Rolle wünschte ich mir in allen Unterrichtsstunden einnehmen zu dürfen.					
(E) Ich kann meiner Rolle derzeit häufig nicht ausreichend gerecht werden.					
(F) Durch meine Rolle sind meine Arbeitsabläufe aufwendiger geworden.					
(G) Ich fühle mich durch meine Rolle häufig zu sehr gestresst.					
(H) Meine Rolle beeinträchtigt meine Freizeit.					
(I) Ich bin motiviert, die Schüler:innen in der Umsetzung des FREI DAY zu begleiten.					

Ansichtsexemplar

E3. Wie viele Stunden pro Woche sind Sie als Lernbegleiter:in aktiv mit der Betreuung der Schüler:innen beschäftigt?

__ __ Schulstunden

E3.1 In welchen Klassenstufen findet der FREI DAY an Ihrer Schule statt?

☐ Klassenstufe 1

☐ Klassenstufe 2

☐ Klassenstufe 3

☐ Klassenstufe 4

☐ Klassenstufe 5

☐ Klassenstufe 6

☐ Klassenstufe 7

☐ Klassenstufe 8

☐ Klassenstufe 9

☐ Klassenstufe 10

☐ Klassenstufe 11

☐ Klassenstufe 12

☐ Klassenstufe 13

☐ Andere ________________________

Ansichtsexemplar

E3.2 Welche Schulfächer werden durch den FREI DAY an Ihrer Schule ersetzt? Bitte geben Sie in der jeweiligen Zelle die entsprechende Anzahl der Schulstunden für das Schulfach und die entsprechende Klassenstufe an, auf die zugunsten des FREI DAY verzichtet wird.

	**Klassenstufe**													
**Schulfächer**	1	2	3	4	5	6	7	8	9	10	11	12	13	Andere
Mathematik														
Deutsch														
Fremd-sprachen														
Physik, Chemie, Biologie														
Geografie, Geschichte, Sozialkunde, Politik														
Wirtschaft oder Technik														
Kunst oder Musik														
Religion oder Ethik														
Sport														
Sonstige Fächer														

Ansichtsexemplar

E4. Wie viele Stunden pro Woche benötigen Sie für vor-/nachbereitende Aufgaben der Lernbegleitung?

__ __ Zeitstunden

E5. Um wie viele Stunden wurde Ihr reguläres Lehrdeputat für Ihre Aufgabe als Lernbegleiter:in reduziert?

__ __ Schulstunden

E6. Hat sich ihr wöchentlicher Arbeitsaufwand durch die Aufgabe als Lernbegleiter:in im Vergleich zu Ihrer Lehrtätigkeit vor der Einführung von FREI DAY erhöht oder reduziert (inklusive Vor- und Nachbereitung)?

☐ Reduziert, und zwar um __ __ Zeitstunden

☐ Erhöht, und zwar um __ __ Zeitstunden

☐ Keine Veränderung

☐ Weiß nicht

E7. Haben Sie vor, zukünftig als Lernbegleiter:in aktiv zu sein?

☐ Ja

☐ Nein → weiter mit **Frage F1**

E8. Inwieweit treffen folgende Aussagen zu den Erwartungen an die Rolle als Lernbegleiter:in auf Sie zu?

	Trifft nicht zu	Trifft eher nicht zu	Trifft eher zu	Trifft zu	Weiß nicht
(A) Mir wird es ein gutes Gefühl geben, unsere Schule durch die Begleitung unserer FREI-DAY-Projekte zu stärken.					
(B) In der Rolle werde ich mich wohlfühlen.					
(C) Die neue Rolle sehe ich am ehesten als Ergänzung zu meiner sonstigen Lehrkraftrolle.					
(D) Durch meine neue Rolle werden meine Arbeitsabläufe aufwendiger.					
(E) Ich werde mich durch meine neue Rolle gestresster fühlen.					
(F) Meine neue Rolle wird meine Freizeit in einem bestimmten Maße beeinträchtigen.					
(G) Ich werde motiviert sein, die Schüler:innen in der Umsetzung des FREI DAY zu begleiten.					

Ansichtsexemplar

Abschnitt F: Fragen zur Umsetzung des FREI DAY

F1. Ihrer Einschätzung nach, wie stark erfährt die Umsetzung des FREI DAY an Ihrer Schule Unterstützung oder Widerstand durch...

Schüler:innen:

Großer Widerstand ——————— Hohe Unterstützung

Eltern:

Großer Widerstand ——————— Hohe Unterstützung

Lehrkräfte und pädagogisches Personal:

Großer Widerstand ——————— Hohe Unterstützung

Kommune:

Großer Widerstand ——————— Hohe Unterstützung

Schulaufsicht:

Großer Widerstand ——————— Hohe Unterstützung

F2. In Schulen können unterschiedliche Rahmenbedingungen für die Einführung des FREI DAY bestehen. Wenn Sie an die letzten 6 Monate denken, inwieweit haben folgende Rahmenbedingungen an Ihrer Schule ein Problem dargestellt?

	Großes Problem	Eher großes Problem	Eher geringes Problem	Kein Problem	Weiß nicht
(A) Das Management der Schulleitung.					
(B) Das Management des FREI-DAY-Teams.					
(C) Lokale Schlüsselpersonen hatten ein mangelhaftes Verständnis vom FREI DAY.					
(D) Konflikte zwischen den Beteiligten an der Umsetzung des FREI DAY.					
(E) Mitglieder des FREI-DAY-Teams identifizieren sich <u>nicht</u> genug mit dem FREI DAY bzw. setzen sich <u>nicht</u> genug dafür ein.					
(F) Die „richtigen“ Personen für unser FREI-DAY-Team zu finden – d.h. Personen mit den nötigen Fähigkeiten, Begabungen oder (politischen) Verbindungen.					

Ansichtsexemplar

	Großes Problem	Eher großes Problem	Eher geringes Problem	Kein Problem	Weiß nicht
(G) Unterstützung von kommunalen Schlüsselpersonen (z. B. Bürgermeister:innen) zu erhalten.					
(H) Unterstützung von lokalen Einrichtungen (z. B. Unternehmen) für die Umsetzung von FREI-DAY-Projekten zu bekommen.					
(I) Beratung und Unterstützung von „Schule im Aufbruch" ist nicht hilfreich oder kommt zu spät.					
(J) Die acht Phasen zur Implementation des FREI DAY umzusetzen.					
(K) Die nötigen Ressourcen zu akquirieren (z. B. Geld oder Zeit für Treffen).					
(L) Alle Schülergruppen für ihre Projekte im Rahmen des FREI DAY zu motivieren.					
(M) Beteiligung seitens der lokalen Einrichtungen und Bevölkerung.					
(N) Personen oder Einrichtungen ließen sich nicht für die Zusammenarbeit begeistern.					

F3. Gab es die folgenden Aktivitäten bei der konkreten Einführung des FREI DAY an Ihrer Schule? Falls ja, geben Sie bitte an, ob Sie beteiligt waren!

	Ja, die Aktivität gab es.	Nein, die Aktivität gab es nicht.	Ich war beteiligt.	Ich war nicht beteiligt.
1. Kollegiumsinterne Diskussionsrunden				
2. Sichtung von Materialien zur Qualität von Schule und Unterricht				
3. Durchführung von bzw. Teilnahme des Kollegiums an Fortbildungsveranstaltungen				
4. Erarbeitung von Teilen des Schulkonzepts in Arbeitsgruppen				
5. Bildung einer Konzept- oder Steuergruppe				
6. Besuche anderer Schulen mit vergleichbaren Angeboten				
7. Elternbefragungen				
8. Schüler:innenbefragungen				

Ansichtsexemplar

	Ja, die Aktivität gab es.	Nein, die Aktivität gab es nicht.	Ich war beteiligt.	Ich war nicht beteiligt.
9. Gespräche mit außerschulischen Kooperationspartnern				
10. Erörterungen mit dem Schulträger				

F4. Inwiefern haben sich nach Ihrer subjektiven Einschätzung in den folgenden Aspekten Veränderungen durch den FREI DAY ergeben?

	verschlechtert	eher verschlechtert	keine Änderung	eher verbessert	verbessert	Weiß nicht
(A) Abstimmung von Nachhaltigkeits- und Gesundheitsaktivitäten an der Schule.						
(B) Die Zusammenarbeit mit außerschulischen Partnern zur Erreichung der SDGs.						
(C) Das Wohlbefinden der Schüler:innen.						
(D) Das Wohlbefinden der Lehrkräfte.						
(E) Die Fähigkeit der Schüler:innen, kollektiv an der Erreichung von den SDGs zu arbeiten.						
(F) Die Erreichung der curricularen Lernziele der Schüler:innen in den regulären Schulfächern insgesamt.						
(G) Die Erreichung der curricularen Lernziele der Schüler:innen in den Schulfächern, die wegen dem FREI DAY ausgefallen sind.						
(H) Der Stellenwert von Projekten an unserer Schule, die nicht der						

Ansichtsexemplar

	verschlechtert	eher ver-schlechtert	keine Änder-ung	eher verbessert	verbessert	Weiß nicht
Erreichung der SDGs dienen.						
(I) Das Ansehen der Schule in der Kommune.						
(J) Bekanntheit der FREI DAY-Projekte zur Erreichung der SDGs in der Kommune.						
(K) Der Umfang der finanziellen Mittel für SDGs an der Schule.						
(L) Die lokale Agenda (z.B. Schule, Kommune, Behörden) bezüglich der Umsetzung der SDGs.						

Ansichtsexemplar

F5. Wie viel Einfluss hat Ihrer Meinung nach der FREI DAY an Ihrer Schule auf die Umsetzung der SDGs in Ihrer Kommune durch folgende Personengruppen/Einrichtungen?

	keinen	wenig	etwas	viel	Weiß nicht
(A) Kommunalpolitik (z.B. Stadtrat, Landrat)					
(B) Ämter und Behörden (z.B. Verkehrsamt, Jugendamt)					
(C) Schulen und Kindergärten					
(D) Unternehmen (z.B. Läden, Restaurants)					
(E) Vereine (z.B. Sportverein, Schützenverein)					
Sonstige:					

F6. Inwiefern beeinflusst der FREI DAY die Gestaltung Ihrer Kommune?

- ☐ überhaupt nicht
- ☐ ein wenig
- ☐ zu einem guten Teil
- ☐ erheblich
- ☐ Weiß nicht

Abschnitt G: Fragen zur Beratung und Begleitung

Die nächsten Fragen beziehen sich auf die Unterstützung, Beratung und Begleitung durch die Schule im Aufbruch gGmbH.

G1. Wie viel Beratung und Unterstützung haben Sie durch „Schule im Aufbruch" erhalten?

☐ gar keine → weiter zu **Frage H1**

☐ eher wenig

☐ eher viel

☐ viel

☐ Weiß nicht

(eher wenig – Weiß nicht:) weiter zu **Frage G2**

G2. Wie zufrieden oder unzufrieden sind Sie mit der Unterstützung durch „Schule im Aufbruch"?

☐ unzufrieden

☐ eher unzufrieden

☐ eher zufrieden

☐ zufrieden

☐ Weiß nicht

G3. Wie oft greifen Sie auf Materialien von „Schule im Aufbruch“ zurück?

☐ Nie

☐ Eher selten

☐ Eher häufig

☐ Häufig

☐ Weiß nicht

Ansichtsexemplar

Abschnitt L: Weiterführende Kontaktdaten

Um das Geschehen an Ihrer Schule möglichst umfassend darstellen zu können, möchten wir systematisch Personen, die Ihre Schule gut einschätzen können, befragen. Gemeint sind hier nicht nur Personen, die an Ihrer Schule tätig sind, sondern ebenso Eltern, Schülerinnen und Schüler oder auch Mitarbeitende der Kommune bzw. des Schulträgers.

Würden Sie uns bitte für zwei Personen die Kontaktdaten nennen?

Art. 14 der DSGVO erlaubt es wissenschaftlichen Studien, personenbezogene Daten über Dritte zu erfragen.

Alternativ können Sie sich auch erst absprechen und uns im Nachhinein Kontaktdaten zusenden. Nutzen Sie dafür bitte folgende E-Mail-Adresse: freiday@mh-hannover.de

L1. Kontaktdaten der ersten Person.

Name: __________________________
Telefon: __________________________
E-Mail-Adresse: __________________________

L2. Bitte nennen Sie uns die Funktion der ersten Person.

☐ Lehrkraft
☐ Schulsozialarbeitskraft
☐ Schulpsychologische Fachkraft
☐ Schüler:innenvertretung
☐ Elternvertretung
☐ Mitglied des Schulfördervereins
☐ Mitarbeitende des Schulträgers
☐ Externe Kooperationspartner:in
☐ Andere Funktion, und zwar: _______________
☐ Weiß nicht

L3. Kontaktdaten von der zweiten Person.

Name: __________________________
Telefon: __________________________
E-Mail-Adresse: __________________________

L4. Bitte nennen Sie uns die Funktion der zweiten Person.

☐ Lehrkraft
☐ Schulsozialarbeitskraft
☐ Schulpsychologische Fachkraft
☐ Schüler:innenvertretung
☐ Elternvertretung
☐ Mitglied des Schulfördervereins

(Fortsetzung Frage L4)

Ansichtsexemplar

☐ Mitarbeitende des Schulträgers

☐ Externe Kooperationspartner:in

☐ Andere Funktion, und zwar: ______________

☐ Weiß nicht

Abschluss

Vielen Dank für Ihre Teilnahme!

Wir möchten uns ganz herzlich für Ihre Mithilfe bedanken.

Ihre Antworten wurden gespeichert, Sie können das Browser-Fenster nun schließen.

Ansichtsexemplar
