# Supplementary material for: Study protocol for a quasi-experimental evaluation of the whole school approach FREI DAY
Source: Arch Public Health. 2026 Oct 13;84:222. doi: 10.1186/s13690-026-02088-7 (PMC13670517; doi:10.1186/s13690-026-02088-7)
Supplement: Supplementary file 12 [file 13690_2026_2088_MOESM8_ESM.pdf]

Supplement 8: Milestones and Benschmarks-Tool for FREI DAY coordinators

Milestones and Benchmarks-Tool

Phase 1 – Informieren								
	Inwieweit trifft die Aussage zur Umsetzung der Phase zu?				Wie schwierig war/ist die Umsetzung?			
	Trifft zu	Trifft eher zu	Trifft eher nicht zu	Trifft nicht zu	Nicht schwierig	Eher nicht schwierig	Eher schwierig	Schwierig
Das Lernformat FREI DAY ist klar (Methoden, Ziele usw.).								
Der Prozess zur Einführung des FREI DAY ist klar.								
Praxisbeispiele zur Umsetzung des FREI DAY sind bekannt.								
Die Unterstützungsangebote von *Schule im Aufbruch* zur Einführung und Umsetzung des FREI DAY sind bekannt.								

Phase 2 – Verbündete finden								
	Inwieweit trifft die Aussage zur Umsetzung der Phase zu?				Wie schwierig war/ist die Umsetzung?			
	Trifft zu	Trifft eher zu	Trifft eher nicht zu	Trifft nicht zu	Nicht schwierig	Eher nicht schwierig	Eher schwierig	Schwierig
Verbündete (Kolleg/-innen, Eltern, Schüler/-innen) für die Einführung des FREI DAY sind an der Schule gefunden.								
Die Schulleitung ist mit eingebunden und überzeugt.								
Erwartungen und Ziele für die Zusammenarbeit sind gemeinsam geklärt.								

Ein Zeitplan zur Einführung des FREI DAY ist gemeinsam erstellt.								
Schule im Aufbruch ist über die Einführung unseres FREI DAY informiert.								
Unsere Schule ist mit anderen FREI DAY Schulen vernetzt.								
Es ist entschieden, den FREI DAY entweder als Pilotversuch zu starten oder schulweit einzuführen.								
Die Zustimmung der Schulleitung liegt vor.								

Wenn FREI DAY als Pilotversuch gestartet werden soll: Weiter mit Phase 3 „Pilotphase vorbereiten".

Wenn FREI DAY in der ganzen Schule umgesetzt werden soll: Weiter mit Phase 6 „Lernformat verankern".

Phase 3 – Pilotphase vorbereiten								
	Inwieweit trifft die Aussage zur Umsetzung der Phase zu?				Wie schwierig war/ist die Umsetzung?			
	Trifft zu	Trifft eher zu	Trifft eher nicht zu	Trifft nicht zu	Nicht schwierig	Eher nicht schwierig	Eher schwierig	Schwierig
Das Kollegium ist über den Pilotversuch informiert.								
Zeitraum und beteiligte Klassen(stufen) für den Pilotversuch sind festgelegt.								
Die Schüler/-innen der Pilotklassen und deren Eltern sind informiert.								
Rollen und Aufgaben der am Pilotversuch beteiligten Lehrkräfte sind festgelegt.								
Der Fortbildungsbedarf für die beteiligten Kolleg/-innen ist identifiziert.								

Angebote, die den Fortbildungsbedarf zum FREI DAY im Kollegium decken, sind geschaffen.								
Externe Kooperationspartner/-innen sind identifiziert.								
Die Hinführung der Schüler/-innen zu den SDGs und zur eigenständigen Projektarbeit ist geplant.								

Phase 4 – Pilotphase umsetzen								
	Inwieweit trifft die Aussage zur Umsetzung der Phase zu?				Wie schwierig war/ist die Umsetzung?			
	Trifft zu	Trifft eher zu	Trifft eher nicht zu	Trifft nicht zu	Nicht schwierig	Eher nicht schwierig	Eher schwierig	Schwierig
Der Pilotversuch ist dokumentiert (Foto, Video).								
Die Lehrkräfte tauschen sich über ihre Erfahrungen aus (kollegiale Fallberatung, Lösungsansätze)								
Der Pilotversuch ist abgeschlossen.								
Gelingensbedingungen und Erfolgsfaktoren des FREI DAY sind identifiziert.								
Die Schulgemeinschaft erkennt das Potential des FREI DAY.								

Phase 5 – Pilotphase evaluieren								
	Inwieweit trifft die Aussage zur Umsetzung der Phase zu?				Wie schwierig war/ist die Umsetzung?			
	Trifft zu	Trifft eher zu	Trifft eher nicht zu	Trifft nicht zu	Nicht schwierig	Eher nicht schwierig	Eher schwierig	Schwierig
Es finden regelmäßige Reflexionsgespräche mit den Schüler/-innen statt.								

Der Pilotversuch ist gemeinsam mit allen Beteiligten evaluiert (Gruppengespräche, World Café, Umfrage).								
Nächste Schritte sind anhand der Evaluation abgeleitet.								
Erfahrungen zum FREI DAY sind gesammelt und der Schulgemeinschaft zugänglich.								
Die Entscheidung als Schulgemeinschaft den FREI DAY weiterzuführen ist getroffen.								
Offene FREI DAY Bausteine sind erarbeitet und überarbeitet.								
Die nächste Evaluation ist terminiert.								

Phase 6 – Lernformat verankern								
	Inwieweit trifft die Aussage zur Umsetzung der Phase zu?				Wie schwierig war/ist die Umsetzung?			
	Trifft zu	Trifft eher zu	Trifft eher nicht zu	Trifft nicht zu	Nicht schwierig	Eher nicht schwierig	Eher schwierig	Schwierig
Das Konzept des FREI DAY ist an die Gegebenheiten der eigenen Schule angepasst.								
Schüler/-innen, Eltern und Kolleg/-innen sind aktiv an der Konzeption des FREI DAY beteiligt.								
Vorschläge für die FREI DAY Stundentafel sind erarbeitet.								
Das Konzeptpapier (Visionen, Ziele, Lernformate, Einführungsphase) ist verschriftlicht.								
Informations- und Austauschformate sind durchgeführt (z.B. Elternabende, Kaffeepausen im Lehrerzimmer, öffentliche Ausstellung von Projektideen, Hospitationen).								
Fragen und Bedenken der Schulgemeinschaft zum FREI DAY sind geklärt.								
Eine 2/3-Mehrheit der Schulgemeinschaft zur Einführung des FREI DAY ist gewonnen.								

Die Einführung des FREI DAY ist durch die Schulkonferenz beschlossen.								

Phase 7 – Umsetzung vorbereiten								
	Inwieweit trifft die Aussage zur Umsetzung der Phase zu?				Wie schwierig war/ist die Umsetzung?			
	Trifft zu	Trifft eher zu	Trifft eher nicht zu	Trifft nicht zu	Nicht schwierig	Eher nicht schwierig	Eher schwierig	Schwierig
Ein Leitfaden für das Kollegium zur Begleitung des FREI DAY (Rolle, Aufgaben etc.) ist entwickelt und verschriftlicht.								
Der Fortbildungsbedarf der Lehrkräfte ist ermittelt.								
Die Fortbildungen für das Kollegium sind erfolgt.								
Kontakte zu Akteur/-innen der außerschulischen Bildungslandschaft sind aufgebaut.								
Zusammenarbeitsmöglichkeiten mit außerschulischen Akteur/-innen sind vereinbart.								
Die (Groß-)Eltern sind aktiviert und in den FREI DAY (insbesondere als Unterstützungsstrukturen) eingebunden.								
Das Kick-Off zur Einführung des FREI DAY ist geplant (Termin, Ablauf).								
Die Schüler/-innen sind methodisch und inhaltlich auf ihre Projektarbeit im FREI DAY vorbereitet.								

Phase 8 – Umsetzen								
	Inwieweit trifft die Aussage zur Umsetzung der Phase zu?				Wie schwierig war/ist die Umsetzung?			
	Trifft zu	Trifft eher zu	Trifft eher nicht zu	Trifft nicht zu	Nicht schwierig	Eher nicht schwierig	Eher schwierig	Schwierig
Der FREI DAY ist von der Schulgemeinschaft und den außerschulischen Akteur/-innen gemeinsam gestartet.								
Räume, Zeiten und Methoden sind geschaffen, die Schüler/-innen dabei unterstützen, eigene Projektideen zu den SDGs zu entwickeln.								
Es bestehen fortlaufende Feedbackmöglichkeiten für alle Beteiligten, um Erfahrungen gemeinsam zu reflektieren und Verbesserungen zu erarbeiten.								
Regelmäßige Reflexionsgespräche mit allen Schüler/-innen-Teams zum gemeinsamen Projektprozess und zu individuellen Lernerfahrungen finden statt.								
