# Supplementary material for: Study protocol for a quasi-experimental evaluation of the whole school approach FREI DAY
Source: Arch Public Health. 2026 Oct 13;84:222. doi: 10.1186/s13690-026-02088-7 (PMC13670517; doi:10.1186/s13690-026-02088-7)
Supplement: Supplementary file 13 [file 13690_2026_2088_MOESM9_ESM.pdf]

Supplement 9: Guide for focus groups in elementary schools

Leitfaden Fokusgruppendiskussion

Zeit ca. 1h	Fragestellungen	Zusätzliche Impulse	Material
4 Min.	**1. Einstieg**<br>• Vorstellung von mir und dem Projekt<br>• Hinweise für den Ablauf<br>• Hinweise auf Anonymität und Aufzeichnung der Fokusgruppe<br>• *Vorstellungsrunde* der Schüler:innen (Name, Alter, Klasse)<br><br>*Wie läuft der FREI DAY an eurer Schule ab?*<br><br>*Seit wann nehmt ihr am FREI DAY teil?*<br><br>*Welches Projekt führt ihr durch?*<br><br>*An welche Nachhaltigkeitsziele (SDGs) knüpfen eure Projekte an?*	Abbildung aller SDGs	Abb.
12 Min.	**2. Partizipativer Einstieg**<br><br>**Reflexive Fotografie**<br>Den Schüler:innen wird folgende Aufgabegestellt:<br>**Was macht für dich den FREI DAY aus?**		iPads des IDN

	Bitte macht ein Foto von dem, was für euch den FREI DAY ausmacht. Ihr könnt zum Beispiel Orte, Gegenstände oder Menschen fotografieren. Falls ihr einzelne Menschen fotografiert, fragt diese bitte vorher, ob die Aufnahme für sie in Ordnung ist. Wenn das, was ihr fotografieren möchtet, nicht auf dem Schulgelände erreichbar ist, könnt ihr auch ein Bild aus dem Internet raussuchen. Achtet jedoch darauf, sensibel mit den Fotos anderer umzugehen.		
10 Min.	**3. Reflexionsfragen zu den aufgenommenen Fotos**<br><br>*Bevor ihr euch gegenseitig eure Bilder präsentiert, überlegt jede:r für sich eine Begründung für die Auswahl des Motivs.*<br><br>*Zeigt bitte euer Bild und begründet, warum ihr euch für dieses Motiv entschieden habt.*<br><br>*Welche Gefühle oder Erlebnisse verbindet ihr mit dem Motiv?*	*Sind eure Gefühle im Zusammenhang mit dem Motiv eher positiv, neutral oder eher negativ?*	
10 Min.	**4. Bewertung des FREI DAY**<br><br>*Was unterscheidet für euch den FREI DAY vom normalen Schulunterricht?*<br><br>*Wie gefällt euch der FREI DAY? Bewertet diesen auf einer Skala von 1 (sehr gut) bis 6 (ungenügend)*<br><br>*Was hat sich durch den FREI DAY für euch verändert?*	*Was genau ist anders?*<br><br>*Was fällt euch schwer/ leicht beim FREI DAY?*<br><br>*Geht ihr lieber in die Schule?*	

		Gibt es für euch weniger Stress im Schulalltag?	
7 Min.	**5. Lernklima in der Gruppe/Lernbegleitung**<br><br>*Wie gefällt euch das Arbeiten in einer Projektgruppe?*<br><br>*Wie geht ihr mit schwierigen Situationen bei der Projektarbeit um?*<br><br>*Wie gut fühlt ihr euch von den Erwachsenen bei eurem Projekt unterstützt und warum? Bewertet auf einer Skala von 1 (sehr gut) bis 6 (ungenügend).*	*Was gefällt euch besonders gut/nicht so gut?*<br><br>*Gibt es auch mal Streit oder Meinungsverschiedenheiten? Was macht ihr dann?*<br><br>*Was machen die Lehrkräfte gut/ nicht gut im Rahmen des FREI DAY?*<br>*Hat sich durch den FREI DAY das Verhältnis zu den Lehrkräften verändert? Falls ja, wie begründet ihr das?*	
12 Min.	**6. Transformatives Handeln**<br><br>*Wie sehr berücksichtigen eure Projekte Aspekte der Nachhaltigkeit?*<br><br>*Wie verändern die Projekte eurer Umfeld in der Schule und zu Hause?*<br><br>*Hat sich durch den FREI DAY verändert, wie ihr auf die Zukunft schaut? Was traut ihr euch jetzt mehr/weniger zu?*	*Welche Nachhaltigkeitsziele (SDGs) verknüpft ihr mit euren Projekten?*<br><br>*Was hat euch konkret bewegt, etwas zu ändern? Gab es besondere Momente, die euch darin bestärkt haben?*	

5 Min	**7. Abschluss**<br><br>*Am Anfang dieser Diskussion habt ihr Fotos gemacht, die zeigen sollten, was für euch den FREI DAY ausmacht. Würdet ihr jetzt, nachdem wir ausführlicher über den FREI DAY gesprochen haben, ein anderes Motiv wählen?*<br><br>Blitzlicht: *Was nehmt ihr aus unserer Diskussionsrunde mit?*	*Falls ja, warum?*	

ZIELE FÜR NACHHALTIGE ENTWICKLUNG

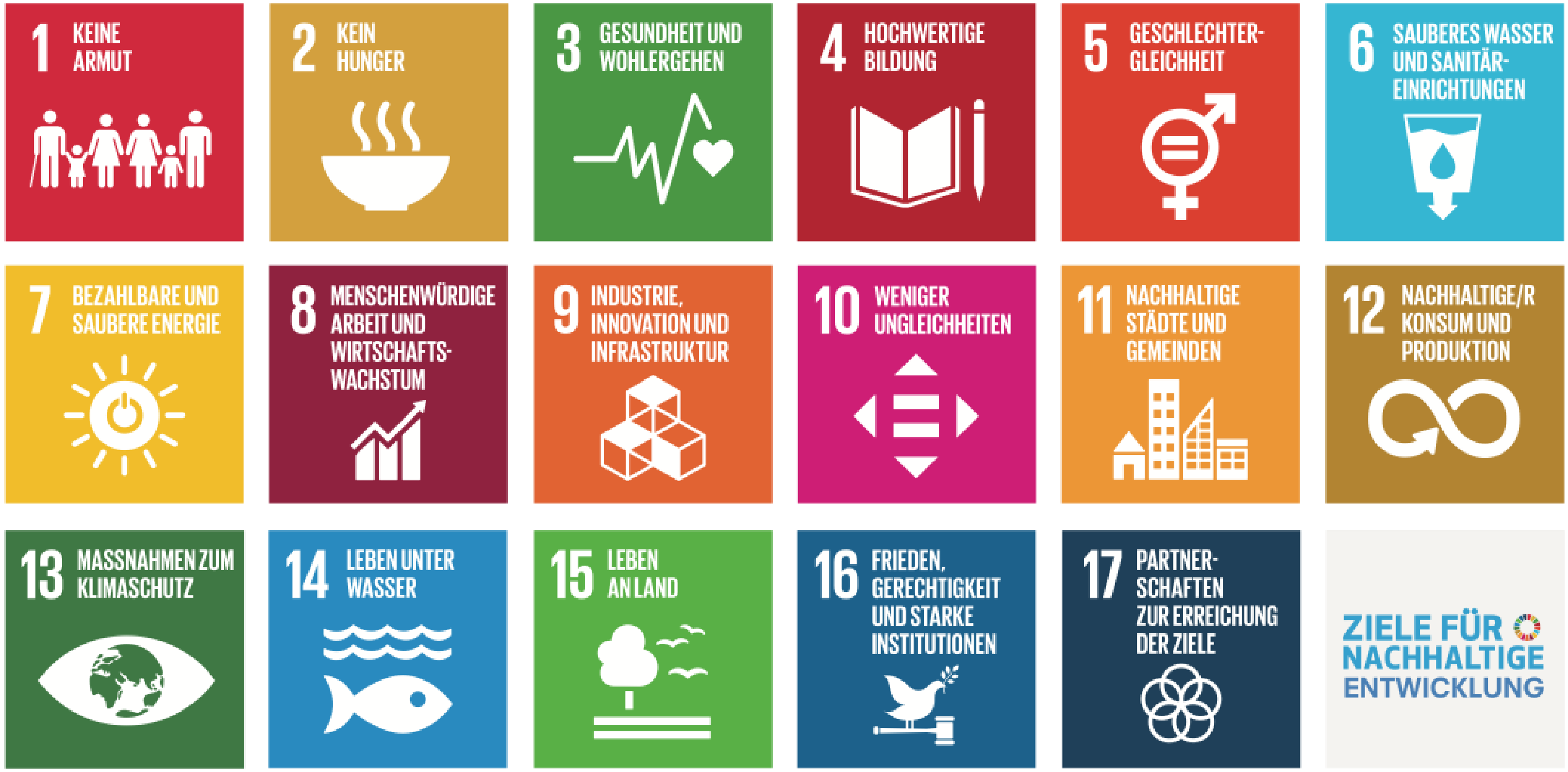

Developed in collaboration with TROLLBÄCK+COMPANY | TheGlobalGoals@trollback.com | +1.212.529.1010
For queries on usage, contact: dpicampaigns@un.org | Non official translation made by UNRIC Brussels (September 2015)

Medizinische Hochschule Hannover

Leibniz Universität Hannover

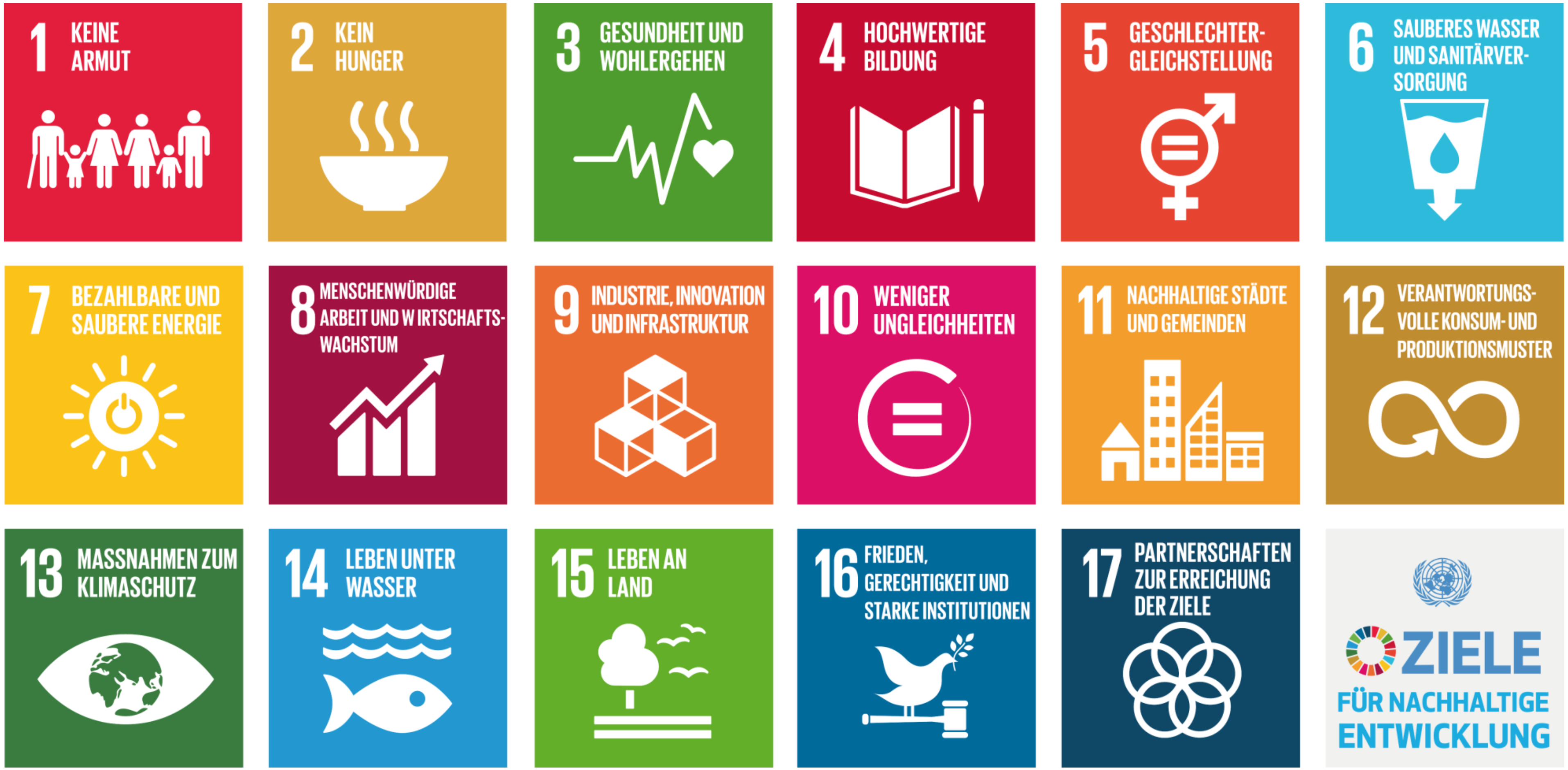

Medizinische Hochschule Hannover

Leibniz Universität Hannover
